# Tuning Scaffold Properties of New 1,4-Substituted Pyrrolo[3,2-c]quinoline Derivatives Endowed with Anticancer Potential, New Biological and In Silico Insights

**DOI:** 10.3390/biom15121718

**Published:** 2025-12-10

**Authors:** Francesco Mingoia, Caterina Di Sano, Claudia D’Anna, Marco Fazzari, Alessia Bono, Gabriele La Monica, Annamaria Martorana, Antonino Lauria

**Affiliations:** 1 Istituto per lo Studio dei Materiali Nanostrutturati (ISMN), Consiglio Nazionale delle Ricerche (CNR), Via U. La Malfa 153, 90146 Palermo, Italy; 2 Istituto di Farmacologia Traslazionale (IFT), Consiglio Nazionale delle Ricerche (CNR), Via U. La Malfa 153, 90146 Palermo, Italy; caterina.disano@ift.cnr.it (C.D.S.); claudia.danna@ift.cnr.it (C.D.); 3 Department of Pharmacology and Chemical Biology, University of Pittsburgh, Pittsburgh, PA 15261, USA; maf167@pitt.edu; 4 Dipartimento di Scienze e Tecnologie Biologiche Chimiche e Farmaceutiche “STEBICEF”, University of Palermo, Viale delle Scienze, Ed. 17, 90128 Palermo, Italy; alessia.bono01@unipa.it (A.B.); gabriele.lamonica01@unipa.it (G.L.M.); annamaria.martorana@unipa.it (A.M.); 5 Fondazione Umberto Veronesi (FUV), via Solferino 19, 20121 Milano, Italy; 6 National Biodiversity Future Center (NBFC), Piazza Marina 61, 90133 Palermo, Italy

**Keywords:** pyrrolo[3,2-c]quinoline, anticancer, benzo[1,3]dioxole-5-yl, NCI, clonogenic test, scratch test, MCF-7, HeLa

## Abstract

A new series of angular tricyclic pyrrolo[3,2-c]quinoline derivatives (PQs) was designed and synthesized to further explore the previously promising antiproliferative activity exhibited by the 4-benzodioxole-substituted hit **7d**. Accordingly, several structural modifications mainly focused on the benzodioxole moiety were introduced, allowing us to gain new insights into the activity and biological profile. NCI antiproliferative screening (SRB colorimetric assay), together with MTS-based assay against six other tumour cell lines, enabled us a deeper understanding of the selectivity and potency patterns. This led to the identification of a new promising hit, compound **7p**, which exhibited cytotoxic activity in the low micromolar range against MCF-7 and HeLa cells. Further biological evaluations, including apoptosis induction, clonogenic, and scratch tests, provided additional biological insights into the anticancer potential of these compounds, supporting the subsequent lead optimization process for more potent anticancer activity. The integrated in silico docking results evidenced a clear multi-target profile, as testified by the broad anticancer activity, and suggest a good potential for rational polypharmacology.

## 1. Introduction

In the last decade, scientific interest in the angular tricyclic pyrrolo[3,2-c]quinoline (PQ) scaffold has grown significantly, as highlighted by the increasing number of reports on the subject in various applicative sectors [1,2]. The combination of the five-membered electron-rich pyrrole ring and the basic quinoline core structure gives this tricyclic framework a significant ability to interact with a variety of biological targets, as also supported by recent studies exploring pyrrolo-fused heterocycles as promising anticancer agents [3]. This makes the PQ system particularly versatile for multi-target biological applications, with its activity tunable through appropriate choice of structural modifications.

Building on our previous exploration of the chemical space around positions 1–4 of the PQ scaffold and considering that the introduction of a benzodioxole (piperonyl) moiety at position 4 significantly enhanced the antiproliferative activity against five tumour panels compared to a phenyl substituent at the same position [4], we designed a new series of PQ derivatives. In this study, structural modifications were mainly focused on the benzodioxole fragment, as detailed below. To further demonstrate the versatility and broad potential of the PQ framework, we employed the previously established synthetic route that allows functionalization at three key positions [5]. Moreover, the relevance of quinoline-based hybrids in anticancer drug discovery has been reinforced by recent advances showing their potent antiproliferative profiles and their progression toward clinical evaluation [6].

**Figure 1:**
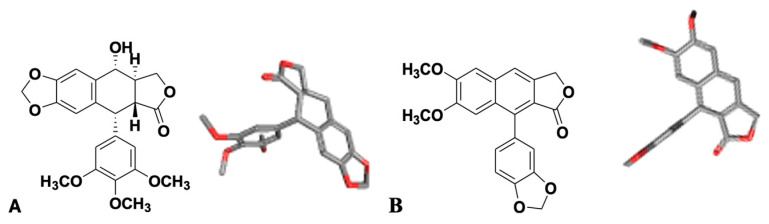
(**A**) Podophyllotoxin; (**B**) Justicidin B.

As is well known, the benzodioxole moiety plays a pivotal role in the remarkable antiproliferative activity of podophyllotoxin (PPT, Figure 1), a natural lignan first isolated from Podophyllum species. PPT exerts its anticancer effects by interfering with microtubule assembly during mitosis, thereby blocking cell division [7]. However, due to its severe side effects, PPT was soon abandoned as a therapeutic agent. Nevertheless, it served as a valuable lead compound for drug optimization, leading to the development of more potent and less toxic derivatives. This medicinal chemistry effort resulted in the synthesis of three clinically used anticancer drugs: etoposide, teniposide, and the water-soluble prodrug etoposide phosphate [7]. Unlike PPT, these agents act through a distinct mechanism of action, namely the inhibition of DNA topoisomerase II [8]. Similarly, the benzodioxole moiety is a key structural feature of justicidin B (Figure 1), another natural lignan endowed with intriguing pharmacological properties, including pronounced antiproliferative activity [9,10,11,12,13].

In this context, a new series of PQ derivatives containing the benzodioxole motif has been designed, synthesized, and evaluated for their antiproliferative activity both through the NCI screening programme (SRB colorimetric tests) and complementary extended assays (MTS). Additional biological assays were also performed to further characterize their anticancer potential and obtain insights into their mechanism of action.

## 2. Materials and Methods

### 2.1. Chemistry: Experimental Procedures for the Synthesis and Characterization of PQ Compounds

**Scheme 1:**
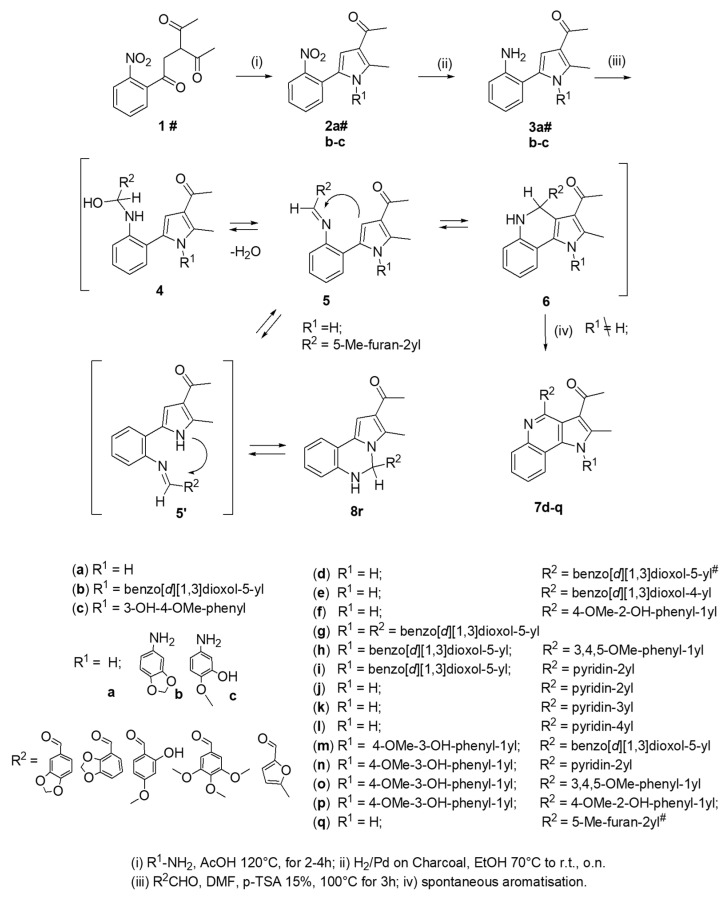
Synthetic route of new PQ derivatives **7d**–**q** and tricyclic derivative **8r**. # [4,5].

Reagents and reactants were purchased from commercial suppliers (Aldrich) and used without further purification. DMF was distilled under vacuum prior to treatment by CaH_2_. Analytical thin-layer chromatography was performed on Merck precoated silica gel (60F254) plates and column chromatography on Merck silica gel 230–400 mesh (ASTM). Melting points were determined with a Sanyo-Gallenkamp capillary apparatus (Sanyo-Gallenkamp, Loughborough, UK) and are uncorrected. IR spectra were recorded using a NaCl cell holder in bromoform with Bruker Alpha FT-IR (Bruker Europe, Ettlingen, Germany). ^13^C NMR spectra were recorded on a Bruker AC 250 spectrometer (Bruker Europe, Ettlingen, Germany) operating in FT mode in DMSO_-d6_ solutions at 250.13 and 62.89 MHz, respectively. ^1^H and ^13^C chemical shift values are given in ppm relative to the solvent peak and DMSO_d6_ (centred at 39.50 ppm downfield from TMS), respectively. Coupling constant values are expressed in Hz. ^13^C chemical shift values were measured from proton fully decoupled spectra. Signal assignments were made based on known substituent effects and one-bond multiplicities (indicated in parentheses) determined by DEPT-135. Mass spectra (EI) were collected on a GC-MS-QP5050A Shimadzu mass spectrometer (Shimadzu Corporation, Kyoto, Japan) with an ionization energy (EI) of 70 eV. Elemental analyses were within ±0.4% of the theoretical values. Yields of compounds refer to purified products and are not optimized. All compound codes mentioned in this section refer to the structures depicted in Scheme 1.

Triketone **1** was prepared according to the previously reported procedure [5,14].

#### 2.1.1. General Procedure for the Preparation of 3-Acetyl-2-methyl-1-R^1^-5-(2-nitro-phenyl)-pyrrole **2a**–**c**

To a solution of diketone **1** (2.63 g, 10 mmol) in acetic acid (50 mL), the suitable amine: amonium acetate 0.77 g (if R1 = H) or aryl-amine (if R1 = benzo[d][1,3]dioxol-5-yl 1.37 g; or if R1 = 3-hydroxy-4-methoxy-phenyl 1.39 g (10 mmol), was added. The mixture was heated under reflux for 1–2 h until the reagents disappeared (TLC monitoring). After cooling, the resulting solution was poured into crushed ice. The so-formed solid was filtered off, dried, and purified by chromatography (eluant petroleum ether (40–60 °C) ethyl acetate 7:3).

(**2a**) 3-Acetyl-2-methyl-1-H-5-(2-nitro-phenyl)-pyrrole was prepared according to previously reported procedure [4,5,15].

(**2b**) 3-acetyl-1-(1-benzo[d][1,3]dioxol-5-yl)-2-methyl-5-(2-nitro-phenyl)-1H-pyrrole.

Recrystallization from EtOH yielded 70% of yellow crystals; mp: 185–186 °C; IR: 1651 (CO), 1525, 1348 (NO_2_) cm^−1^; ^1^H NMR (300 MHz, CDCl_3_) δ 7.76 (1H, d, *J* = 8.0 Hz, H-3″), 7.49 (1H, t, *J* = 7.5 Hz, H-5″), 7.38 (1H, t, *J* = 7.7 Hz, H-4″), 7.31 (1H, d, *J* = 7.6 Hz, H-6″), 6.69 (1H, d, *J* = 7.8 Hz, H-7′), 6.60 (1H, d, J = 0.6 Hz, H-4′), 6.55 (1H, s, H-4), 6.52 (1H, dd, *J* = 1.0, 1.9, H-6), 5.59 (2H, s, CH_2_), 2.44 (3H, s, COCH_3_), 2.42 (3H, s, COCH_3_); ^13^C NMR (75 MHz, CDCl_3_) ppm 194.86 (s), 149.13 (s), 147.91 (s), 147.52 (s), 138.04 (s), 133.27 (d), 132.28 (d), 129.91 (d), 128.27 (d), 128.45 (s), 127.17 (s), 124.6 (d), 121. 96 (d), 121.53 (s), 110.88 (d), 108.98 (d),108.04 (d), 101.84 (t), 28.62 (q), 12.98 (q); *m*/*z* (EI): 364 (24.11 M^+^), 334 (17.13), 305 (24.58), 276 (10.43), 247 (10.47), 228 (36.65), 186 (100.00), 162 (30.42), 95 (22.36), 65 (25.54%). Anal. Calculated for C_20_H_16_N_2_O_5_, Mol. Wt.: 364.35: C, 65.93; H, 4.43; N, 7.69; Found: C, 65.72; H, 4.56; N, 7.95.

(**2c**) 3-Acetyl-1-(3-hydroxy-4-methoxy-phenyl)-2-methyl-5-(2-nitro-phenyl)-1H-pyrrole.

Recrystallization from EtOH yielded 75% of yellow crystals; mp: 188–189 °C; IR: 3180 (OH), 1650 (CO), 1510, 1344 (NO_2_) cm^−1^; ^1^H NMR (300 MHz, CDCl_3_) δ 7.74 (dd, *J* = 8.1, 1.3 Hz, 1H), 7.48 (td, *J* = 7.5, 1.4 Hz, 1H), 7.36 (td, *J* = 7.5, 1.4 Hz, 1H), 7.32 (dd, *J* = 7.6, 1.4 Hz, 1H), 6.75 (d, *J* = 8.5 Hz, 1H), 6.64 (d, *J* = 2.4 Hz, 1H), 6.61 (s, 1H), 6.59 (dd, *J* = 8.5, 2.4 Hz, 1H), 5.88 (s, 1H), 3.87 (s, 3H), 2.46 (s, 3H), 2.42 (s, 3H). ^13^C NMR (75 MHz, CDCl_3_) ppm 195.02 (s), 149.13 (s), 146.47 (s), 145.72 (s), 138.17 (s), 133.28 (d), 132.22 (d), 129.51 (d), 128.57 (s), 128.43 (s), 127.29 (s), 124.01 (d), 121.40 (s), 120.08 (d), 114.54 (d), 110.87 (d), 110.32 (d), 55.88 (q), 28.62 (q), 13.03 (q); *m*/*z* (EI): 366 (21.65, M^+^), 336 (17.24), 307 (25.08), 292 (12.04), 263 (10.67), 228 (26.54), 204 (29.24), 186 (100.00), 150 (24.01), 130 (19.03), 102 (13.85), 77 (14.81), 43 (1.16%). Anal. Calculated for C_20_H_18_N_2_O_5_, Mol. Wt.: 366.37: C, 65.57; H, 4.95; N, 7.65; Found: C, 65.70; H, 4.92; N, 7.40.

#### 2.1.2. General Procedure for the Preparation of 3-Acetyl-2-methyl-1-R1-5-(2-aminophenyl)-pyrrole **3a**–**c**

Nitro compounds: **2a** 2.44 g 10 mmol or **2b** 2.5 g 6.8 mmol or **2c** 2.5 g 6.8 mmol were dissolved in ethanol (200 mL) by heating in a Parr bottle (500 mL), added with a catalytic amount of Pd/C (10%). After vacuum, the bottle was pressurized with hydrogen (4 atm) and reduced overnight. Removal of the catalyst by filtration and evaporation of the solvent under reduced pressure leaves a residue which was purified chromatographically with silica gel (eluent Petroleum-Ether 40-60/EtOAc, 6:4).

(**3a**) 3-acetyl-5-(2-amino-phenyl)-2-methyl-1H-pyrrole [5].

(**3b**) 3-acetyl-[5-(2-amino-phenyl)-1-benzo[d][1,3]dioxol-5-yl-2-methyl-1H-pyrrole.

Recrystallization from EtOH yielded 84% of white crystals; mp: 182–183 °C; IR: 3439, 3349, 1649, 1502, 1488, 1411, 1248, 1211, 1037 cm^−1^; ^1^H NMR (300 MHz, CDCl_3_) δ 7.03 (td, *J* = 7.7, 1.6 Hz, 1H), 6.81 (dd, *J* = 7.4, 1.7 Hz, 1H), 6.71 (dd, *J* = 7.7, 0.9 Hz, 1H), 6.65 (s, 1H), 6.64 (dd, *J* = 7.8, 1.2 Hz, 1H), 6.60 (s, 1H), 6.59 (dd, *J* = 4.1, 1.2 Hz, 1H), 6.55 (dd, *J* = 7.5, 1.1 Hz, 1H), 5.97 (s, 2H, CH_2_), 3.87 (s, 2H, NH_2_), 2.47 (s, 3H), 2.43 (s, 3H). ^13^C NMR (75 MHz, CDCl_3_) ppm 195.27, 147.80, 147.45, 145.69, 137.33, 131.98, 130.96, 130.08, 129.22, 121.60, 121.23, 117.76, 117.62, 115.10, 110.70, 108.91, 107.98, 101.87, 28.81, 13.24. *m*/*z* (EI): 334 (45.30 M^+^), 274 (11.87), 137 (12.50), 115 (12.63), 103 (9.59), 90 (12.12), 77.10 (10.30), 65 (39.01), 43 (100%). Anal. Calculated for C_20_H_18_N_2_O_3_, Mol. Wt.: 334.37: C, 71.84; H, 5.43; N, 8.38; Found C, 71.62; H, 5.39; N, 8.43.

(**3c**) 3-acetyl-[5-(2-amino-phenyl)-1-(3-hydroxy-4-methoxy-phenyl)-2-methyl-1H-pyrrole.

Recrystallization from EtOH yielded 79% of light brown crystals; mp: 237–238 °C; IR: 3412, 3328, 3050, 2966, 2918, 1643, 1510, 1279, 1223, 1099, 1025, 811, 760 cm^−1^; ^1^H NMR (300 MHz, CDCl_3_) δ 7.02 (1H, ddd, *J* = 7.5, 7.7, 1.4 Hz, H-4″), 6.82 (1H, dd, *J* = 7.6, 1.5 Hz, H-6″), 6.73 (1H, d, *J* = 12.1 Hz, H-3′), 6.72 (1H, d, *J* = 1.0 Hz, H-6′), 6.65 (1H, s, H-4), 6.63 (1H, dd, *J* = 9.9,2.23 Hz, H-3″), 6.59 (1H, dd, *J* = 9.6,2.83 Hz, H-4′), 6.55 (1H, ddd, *J* = 7.6, 6.4, 1.4 Hz, H-5″), 5.68 (s, 1H, OH), 3.87 (3H + 2H, s, OCH_3_+ NH_2_), 2.43 (3H, s, COCH_3_), 2.34 (3H, s, CH_3_); ^13^C NMR (DMSO) ppm 192.94 (s), 146.29 (s), 146.10 (s), 145.11 (s), 134.62 (s), 130.40 (s), 129.18 (s), 128.80 (s), 127.47 (d), 119.43 (s), 117.77 (d), 115.24 (s x2), 114.22 (d), 113.29 (d), 110.28 (d), 109.20 (d), 54.43 (q), 27.55 (q), 11.76 (q); *m*/*z* (EI): 336 (28.59 M^+^), 321 (12.11), 293 (6.27), 276 (4.80), 219 (2.51), 197 (3.55), 168 (4.26), 139 (7.52), 102 (10.23), 52 (25.92), 43 (100%). Anal. Calculated for C_20_H_20_N_2_O_3_, Mol. Wt.: 336.38: C, 71.41; H, 5.99; N, 8.33; Found: C, 71.15; H, 5.93; N, 8.41.

#### 2.1.3. General Procedure for the Preparation of PQs (**7d**–**q**)

To a solution of (2-amino-phenyl)-pyrrole **3a**–**c** (**3a** 129 mg, **3b** 200 mg, **3c** 202 mg 0.60 mmol), in dry DMF (5 mL), the suitable aldehyde (0.65 mmol) and a catalytic amount of p-TsOH (15 mol%) were added. Initially, the mixture was stirred at r.t. for 10 min and after heated at 100 °C for 3 h. When the reaction was judged complete (TLC monitoring), the mixture was allowed to reach room temperature. Evaporation of the solvent under reduced pressure gave rise to a dark residue, which was dissolved in dichloromethane (30 mL) and washed with 3 × 10 mL of 5% aqueous NaHCO_3_ solution. The organic extracts dried with MgSO_4_ and evaporated in vacuo afforded a dark solid which was purified by column chromatography (eluant DCM/EtOAc 9:1 or EtPet 40-60/EtOAc 7:3).

(**7d**) 3-acetyl-4-(benzo[d][1,3]dioxol-5-yl)-2-methyl-1H-pyrrolo[3,2-c]quinoline [4].

(**7e**) 3-acetyl-1-(4-benzo[d][1,3]dioxol-4-yl)-2-methyl-1H-pyrrolo[3,2-c]quinoline.

Recrystallization from EtOH yielded 60% of light brown powder; mp: 275–277 °C; IR: 3238, 1735, 1620, 1507, 1464, 1447, 1251, 1070, 956, 764 cm^−1^; 1HNMR (200 MHz, DMSO) δ 12.83 (s, 1H), 8.41 (s, 1H), 8.05 (s, 1H), 7.66 (s, 2H), 7.26 (s, 1H), 7.01 (s, 2H), 5.89 (s, 2H), 2.63 (s, 3H), 1.99 (s, 3H), ^13^C NMR (50 MHz, DMSO) ppm 195.22, 148.91, 146.97, 144.93, 143.01, 138.87, 134.98, 129.28, 127.21, 126.20, 124.06, 122.53, 121.64, 120.86, 118.37, 116.73, 116.06, 108.43, 100.83, 30.94, 13.55. Anal. Calculated for C_21_H_16_N_2_O_3_, Mol. Wt.: 344.36: C, 73.24; H, 4.68; N, 8.13; Found: C, 73.47; H, 4.75; N, 8.01.

(**7f**) 3-acetyl-(4-(2-hydroxy-4-methoxy-phenyl-1yl)-2-methyl-1H-pyrrolo[3,2-c]quinoline.

Recrystallization from EtOH yielded 95% of orange powder; mp: 100–102 °C; IR: 3401, 1661, 1564, 1535, 1464, 1415, 1369, 1252, 1220, 764, 742 cm^−1^; 1H NMR (200 MHz, DMSO) δ 12.90 (1H, s, NH), 11.45 (1H, bs, OH), 8.41 (dd, *J* = 2.5, 5.7 Hz, 1H), 8.04 (dd, *J* = 3.7, 5.0 Hz, 1H), 7.67 (dd, *J* = 4.2, 7.8 Hz, 2H), 7.07 (dd, *J* = 2.1, 7.5 Hz, 2H), 6.85 (t, *J* = 7.8 Hz, 1H), 3.85 (s, 3H), 2.57 (s, 3H), 1.78 (s, 3H). 13C NMR (50 MHz, DMSO) ppm 196.37, 171.91, 152.53, 148.26, 145.85, 141.87, 135.49, 128.33, 128.31, 127.45, 126.16, 125.87, 121.90, 120.87, 118.42, 116.17, 116.10, 112.82, 55.76, 31.34, 12.96. Anal. Calculated for C_21_H_18_N_2_O_3_, Mol. Wt.: 346.38: C, 72.82; H, 5.24; N, 8.09; Found: C, 72.55; H, 5.22; N, 8.11.

(**7g**) 3-acetyl-1,4-(benzo[d][1,3]dioxol-5-yl)-2-methyl-1H-pyrrolo[3,2-c]quinoline.

Recrystallization from EtOH yielded 80% of orange powder; mp: 166–167 °C; IR: 2897, 1663, 1502, 1488, 1454, 1387, 1369, 1246, 1218, 1038, 936 cm^−1^; ^1^H NMR (300 MHz, CDCl_3_) δ 8.20 (1H, dd, *J* = 8.3, 1.4 Hz, H-6), 7.55 (1H, ddd, *J* = 8.4, 6.8, 1.5 Hz, H-8), 7.43 (1H, d, *J* = 1.7 Hz, H-4″), 7.27 (1H, dd, *J* = 7.6, 2.6 Hz, H-6″), 7.25 (1H, ddd, *J* = 7.6, 7.1, 1.5 Hz, H-7), 7.17 (1H, dd, *J* = 8.6, 1.4 Hz, H-9), 7.06 (1H, d, *J* = 8.2 Hz, H-7″), 6.94 (1H, d, *J* = 2.9 Hz, H-7′), 6.91 (1H, dd, *J* = 8.0, 2.8 Hz, H-6′), 6.87 (1H, d, *J* = 2.0 Hz, H-4′), 6.21 (2H, dd, *J* = 14.7, 1.2 Hz, -CH_2_′), 6.06 (2H, s, CH_2_′), 2.32 (3H, s, COCH_3_), 1.84 (3H, s, CH_3_); ^13^C NMR (75 MHz, CDCl_3_) ppm 199.66 (s),153.66 (s), 153.52(s), 149.05(s), 148.87 (s), 148.34 (s), 144.81(s), 143.62(s), 139.40 (s), 136.22 (s), 136.11(s), 131.74(s), 130.14 (d), 126.9 (d), 125.57 (d), 125.40 (d), 122.87 (d), 122.17 (d), 120.40 (d), 118.92 (s), 116.39 (s), 109.17 (d), 109.03 (d), 108.62 (d), 102.35 (t), 101.29 (t), 31.95 (q), 11.70 (q). Anal. Calculated for C_28_H_20_N_2_O_5_, Mol. Wt.: 464.47: C, 72.41; H, 4.34; N, 6.03; Found: C, 72.25; H, 4.39; N, 5.85.

(**7h**) 3-acetyl-4-(benzo[d][1,3]dioxol-5-yl)-1-(3-hydroxy-4-methoxy-phenyl-1yl)-2-methyl-1H-pyrrolo[3,2-c]quinoline.

Recrystallization from EtOH yielded 74% of yellow crystals; mp: 296–297 °C; IR: 3650-2860, 1666, 1509, 1489, 1447, 1390, 1277, 1245, 1224, 1033, 760 cm^−1^; ^1^H NMR (300 MHz, DMSO) δ 9.80 (1H, s, OH), 8.04 (1H, dd, *J* = 8.2 Hz, H-6), 7.54 (1H, ddd, *J* = 7.8, 7.2 Hz, H-8), 7.28 (1H, d, *J* = 1.5 Hz, H-4′), 7.27 (1H, dd, *J* = 15.5 Hz, H-9), 7.21 (1H, d, *J* = 8.6 Hz, H-7″), 7.16 (1H, dd, *J* = 6.9, 1.6 Hz, H-6″), 7.05 (1H, ddd, *J* = 8.4, 8.0 Hz, H-7), 7.04 (1H, d, *J* = 8.0 Hz, H-3′), 6.98 (1H, dd, *J* = 8.3, 2.3 Hz, H-4′), 6.91 (1H, d, *J* = 2.3 Hz, H-6′), 6.13 (2H, s, CH_2_), 3.92 (3H, s, OCH_3_), 2.20 (3H, s, CH_3_), 1.76 (3H, s, CH_3_); ^13^C NMR (75 MHz, DMSO) ppm 197.80 (s), 152.96 (s), 148.97 (s), 147.91 (s), 147.81 (s), 147.76 (s), 144.12 (s), 139.83 (s), 135.98 (s), 135.26 (s), 130.18 (s), 129.72 (d), 126.91 (s), 125.47 (d), 122.51 (d), 120.25 (d), 119.12 (d), 117.85 (s), 116.10 (s), 115.69 (s), 115.09 (d), 112.79 (d), 108.66 (d), 108.37 (d), 101.45 (t), 55.70 (q), 31.68 (q), 11.30 (q). Anal. Calculated for C_28_H_22_N_2_O, Mol. Wt.: 466.48: C, 72.09; H, 4.75; N, 6.01; Found: C, 72.27; H, 4.85; N, 5.86.

(**7i**) 3-acetyl-1-(benzo[d][1,3]dioxol-5-yl)-2-methyl-4-(pyridin-2-yl)-1H-pyrrolo[3,2-c]quinoline.

Recrystallization from EtOH yielded 52% of pale yellow powder; mp:209–210 °C; IR: 2918, 1670, 1557, 1503, 1490, 1451, 1430, 1412, 1390, 1371, 1351, 1286, 1248, 1214, 1171, 110, 1071, 1038, 761, 747 cm^−1^; ^1^H NMR (200 MHz, CDCl_3_) δ 8.68 (dd, *J* = 4.8, 0.8 Hz, 1H), 8.41 (d, *J* = 7.9 Hz, 1H), 8.22 (d, *J* = 7.9 Hz, 1H), 7.92 (td, *J* = 7.7, 1.8 Hz, 1H), 7.54 (ddd, *J* = 8.4, 6.6, 1.7 Hz, 1H), 7.41–7.29 (m, 1H), 7.28–7.21 (m, 1H), 7.18 (dd, *J* = 8.5, 1.2 Hz, 1H), 7.03 (d, *J* = 8.1 Hz, 1H), 6.90 (dd, *J* = 8.1, 2.0 Hz, 1H), 6.85 (d, *J* = 1.9 Hz, 1H), 6.18 (dd, *J* = 9.7, 1.3 Hz, 2H), 2.34 (s, 3H), 1.96 (s, 3H). ^13^C NMR (50 MHz, CDCl_3_) ppm 198.36, 158.61, 151.62, 149.06, 148.86, 148.38, 144.54, 139.33, 137.30, 136.64, 131.93, 130.45, 126.71, 125.97, 123.87, 123.23, 122.34, 120.52, 119.10, 117.12, 115.64, 109.34, 109.19, 102.37, 32.16, 11.67. Anal. Calculated for C_26_H_19_N_3_O_3_, Mol. Wt.: 421.45: C, 74.10; H, 4.54; N, 9.97; Found: C, 74.01; H, 4.65; N, 9.81.

(**7j**) 3-acetyl-2-methyl-4-(pyridin-2-yl)-1H-pyrrolo[3,2-c]quinoline.

Recrystallization from EtOH yielded 80% of light yellow powder; mp: 228–229 °C; IR: 3471, 1631, 1597, 1560, 1509, 1487, 1467, 1433, 1384, 1374, 787, 766 cm^−1^; ^1^H NMR (200 MHz, DMSO) δ 12.74 (s, 1H, NH), 8.57 (d, *J* = 4.4 Hz, 1H), 8.41 (dd, *J* = 6.5, 3.1 Hz, 1H), 8.30 (d, *J* = 7.9 Hz, 1H), 8.10 (dd, *J* = 6.6, 3.0 Hz, 1H), 8.01 (td, *J* = 7.8, 1.7 Hz, 1H), 7.68 (t, *J* = 3.4 Hz, 1H), 7.63 (t, *J* = 3.4 Hz, 1H), 7.47 (ddd, *J* = 7.4, 4.7, 0.9 Hz, 1H), 2.55 (s, 3H), 1.82 (s, 3H). ^13^C NMR (50 MHz, DMSO) ppm 196.68 s, 158.30 s, 151.40 s, 147.96 d, 142.75 s, 137.24 s, 137.21 d, 135.43 s, 129.46 d, 127.17 d, 126.39 d, 124.02 d, 123.00 d, 120.87 d, 118.63 s, 116.70 s, 115.52 s, 31.73 q, 12.63 q. Anal. Calculated for C_19_H_15_N_3_O, Mol. Wt.: 301.34: C, 75.73; H, 5.02; N, 13.94; Found: C, 75.93; H, 5.15; N, 14.16.

(**7k**) 3-acetyl-2-methyl-4-(pyridin-3-yl)-1H-pyrrolo[3,2-c]quinoline.

Recrystallization from EtOH yielded 60% of orange-brown powder; mp: 159–160 °C; IR: 3440, 1645, 1595, 1504, 1437, 1178, 774 cm^−1^; ^1^H NMR (200 MHz, DMSO) δ 12.97 (s, 1H, NH), 8.80 (d, *J* = 1.6 Hz, 1H), 8.65 (dd, *J* = 4.7, 1.3 Hz, 1H), 8.42 (dd, *J* = 6.5, 3.0 Hz, 1H), 8.11 (dd, *J* = 6.1, 2.5 Hz, 1H), 8.04 (dt, *J* = 7.9, 2.0 Hz, 1H), 7.69 (t, *J* = 3.5 Hz, 1H), 7.64 (t, *J* = 3.9 Hz, 1H), 7.52 (dd, *J* = 7.6, 5.1 Hz, 1H), 2.64 (s, 3H), 1.88 (s, 3H). ^13^C NMR (50 MHz, DMSO) ppm 195.62, 150.92, 149.28, 148.97, 143.22, 139.87, 137.67, 135.69, 135.43, 129.33, 127.38, 126.33, 123.47, 120.90, 117.44, 116.16, 116.01, 31.34, 13.43. Anal. Calculated for C_19_H_15_N_3_O, Mol. Wt.: 301.34: C, 75.73; H, 5.02; N, 13.94; Found: C, 75.57; H, 5.15; N, 14.16.

(**7l**) 3-acetyl-2-methyl-4-(pyridin-4-yl)-1H-pyrrolo[3,2-c]quinoline.

Recrystallization from EtOH yielded 50% of light yellow powder; mp: >280 °C; IR: 3434, 1645, 1608, 1469, 1437, 1417, 1382, 1365, 1065, 980, 795 cm^−1^; ^1^H NMR (200 MHz, DMSO) δ 12.99 (s, 1H), 8.68 (dd, *J* = 4.4, 1.5 Hz, 2H), 8.43 (dd, *J* = 6.4, 3.2 Hz, 1H), 8.10 (dd, *J* = 6.4, 3.1 Hz, 1H), 7.68 (dq, *J* = 6.9, 3.6 Hz, 2H), 7.60 (dd, *J* = 4.4, 1.6 Hz, 2H), 2.64 (s, 3H), 1.91 (s, 3H). ^13^C NMR (50 MHz, DMSO) ppm 195.54, 151.24, 149.58 x2, 148.97, 142.98, 139.86, 135.46, 129.37, 127.37, 126.53, 123.01 x2, 120.84, 117.25, 116.10, 99.49, 31.47, 13.17. Anal. Calculated for C_19_H_15_N_3_O, Mol. Wt.: 301.34: C, 75.73; H, 5.02; N, 13.94; Found: C, 75.57; H, 5.16; N, 14.11.

(**7m**) 1-acetyl-1-(benzo[d][1,3]dioxol-5-yl)-2-methyl-4-(3,4,5-trimethoxy-phenyl)-1H-pyrrolo[3,2-c]quinoline.

Recrystallization from EtOH yielded 60% of white powder; mp: 178–180 °C; IR: 2937, 1664, 15,871,491, 1462, 1388, 1248, 1236, 1175, 763 cm^−1^; ^1^H NMR (300 MHz, DMSO) δ 8.10 (1H, dd, *J* = 8.3, 1.3 Hz, H-6), 7.58 (1H, ddd, *J* = 8.3, 6.9, 1.3 Hz, H-8), 7.33 (1H, ddd, *J* = 8.5, 7.0, 1.3 Hz, H-7), 7.29 (1H, d, *J* = 2.0 Hz, H-4′), 7.23 (1H, d, *J* = 8.2 Hz, H-7′), 7.13 (1H, dd, *J* = 8.5, 1.5 Hz, H-9), 7.09 (1H, dd, *J* = 8.2, 2.0 Hz, H-6′), 7.02 (2H, s, H-2″, H-6″), 6.27 (2H, d, *J* = 15 Hz, CH_2_), 3.82 (6H, s, OCH_3_ x2), 3.75 (3H, s, OCH_3_), 2.21 (3H, s, COCH_3_), 1.69 (3H, s, CH_3_); ^13^C NMR (75 MHz, DMSO) ppm 198.77 (s), 153.20 (s), 153.09 (s), 148.71 (s), 148.57 (s), 144.15 (s), 139.56 (s), 138.19 (s), 137.17 (s), 135.30 (s), 131.22 (s), 129.88 (s), 129.85 (d), 126.96 (d), 125.68 (d), 122.31 (d), 120.23 (d), 118.08 (s), 116.16 (s), 115.75 (s), 109.34 (d), 109.24 (d), 105.76 (d x2), 102.44 (t), 60.29 (q), 55.80 (q x2), 31.62 (q), 11.37 (q). Anal. Calculated for C_30_H_26_N_2_O_6_, Mol. Wt.: 510.54: C, 70.58; H, 5.13; N, 5.49; Found: C, 70.39; H, 5.05; N, 5.32.

(**7n**) 1-acetyl-1-(3-hydroxy-4-methoxy-phenyl-1yl)-2-methyl-4-(pyridin-2-yl)-1H-pyrrolo[3,2-c]quinoline.

Recrystallization from EtOH yielded 45% of pale yellow powder, mp:249–250 °C; IR: 3524, 2961, 1686, 1597, 1557, 1513, 1463, 1279, 1247, 1219, 1196, 1114, 810, 769, 753 cm^−1^; ^1^H NMR (200 MHz, CDCl_3_) δ 8.66 (d, *J* = 4.8 Hz, 1H), 8.38 (d, *J* = 7.4 Hz, 1H), 8.23 (d, *J* = 8.4 Hz, 1H), 7.91 (td, *J* = 7.6, 1.2 Hz, 1H), 7.52 (t, *J* = 7.5 Hz, 1H), 7.41–7.24 (m, 2H), 7.20 (d, *J* = 6.9 Hz, 1H), 7.12 (d, *J* = 7.9 Hz, 1H), 7.04 (d, *J* = 8.4 Hz, 1H), 6.94 (d, *J* = 2.2 Hz, 1H), 6.87 (dd, *J* = 8.4, 2.1 Hz, 1H), 4.02 (s, 3H), 2.27 (s, 3H), 1.95 (s, 3H). ^13^C NMR (50 MHz, CDCl_3_) ppm 198.77, 158.52, 151.59, 148.39, 147.97, 147.08, 144.37, 139.62, 137.28, 136.61, 131.32, 130.11, 126.78, 125.91, 123.84, 123.30, 120.66, 120.17, 118.85, 117.17, 115.62, 114.98, 111.37, 56.15, 32.06, 11.80. Anal. Calculated for C_26_H_21_N_3_O_3_, Mol. Wt.: 423.46: C, 73.74; H, 5.00; N, 9.92; Found: C, 73.91; H, 5.23; N, 10.04.

(**7o**) 1-acetyl-1-(3-hydroxy-4-methoxy-phenyl)-2-methyl-4-(3,4,5-trimethoxy-phenyl)-1H-pyrrolo[3,2-c]quinoline.

Recrystallization from EtOH yielded 75% of white powder; mp: 149–150 °C; IR: 3518, 2934, 1662, 1588, 1511, 1496, 1282, 1238, 1029, 762 cm^−1^; ^1^H NMR (300 MHz, DMSO) δ 9.80 (1H, bs OH), 8.08 (1H, dd, *J* = 8.4, 1.4 Hz, H-6), 7.55 (1H, ddd, *J* = 8.2, 6.7, 1.5 Hz, H-7), 7.29 (1H, ddd, *J* = 8.1, 6.6, 8.1 Hz, H-8), 7.21 (1H, d, *J* = 8.5 Hz, H-3′), 7.08 (1H, dd, *J* = 8.4, 1.4 Hz, H-9), 7.02 (2H, s, H-2″, H-5″), 6.98 (1H, dd, *J* = 8.4, 2.4 Hz, H-4′), 6.91 (1H, d, *J* = 2.4 Hz, H-6′), 3.92 (3H, s, OCH_3_), 3.81 (6H, s, OCH_3_), 3.74 (3H, s, OCH_3_), 2.18 (3H, s, COCH_3_), 1.68 (3H, s, CH_3_); ^13^C NMR (63 MHz, DMSO) ppm 198.65 (s), 153.25 (s), 153.20 (s), 149.06 (s), 147.93 (d), 144.16 (s), 139.52 (s), 138.26 (s), 137.26 (s), 135.22 (s), 130.26 (s), 129.84 (d), 126.94 (d), 125.59 (d), 120.30 (d), 119.13 (d), 118.04 (s), 116.24 (s), 115.74 (s), 115.15 (d), 112.87 (d), 105.87 (d), 60.26 (q), 55.99 (q x2), 31.31 (q), 18.26 (q), 10.98 (q). Anal. Calculated for C_30_H_28_N_2_O_6_, Mol. Wt.: 512.55: C, 70.30; H, 5.51; N, 5.47; Found: C, 70.05; H, 5.65; N, 5.62.

(**7p**) 1-acetyl-(1-(3-hydroxy-4-methoxy-phenyl)-4-(2-hydroxy-4-methoxy-phenyl)-2-methyl-1H-pyrrolo[3,2-c]quinoline.

Recrystallization from EtOH yielded 63% of light yellow-brown powder; mp: 257–259 °C; IR: 3479, 3354, 1662, 1643, 1597, 1560, 1508, 1461, 1440, 1260, 1224, 1047, 1021, 746 cm^−1^; ^1^H NMR (200 MHz, CDCl_3_) δ 8.04 (dd, *J* = 8.6, 0.8 Hz, 1H), 7.53 (ddd, *J* = 8.4, 6.8, 1.5 Hz, 1H), 7.32 (dd, *J* = 7.7, 1.7 Hz, 1H), 7.21 (ddd, *J* = 8.1, 6.8, 1.2 Hz, 1H), 7.14–7.09 (m, 1H), 7.07 (d, *J* = 2.7 Hz, 1H), 7.00 (d, *J* = 2.4 Hz, 1H), 6.97–6.90 (m, 2H), 6.88 (d, *J* = 3.5 Hz, 1H), 4.06 (s, 3H), 3.99 (s, 3H), 2.32 (s, 3H), 1.93 (s, 3H). ^13^C NMR (50 MHz, CDCl_3_) ppm 199.63, 153.12, 149.17, 148.04, 147.42, 147.15, 142.40, 140.94, 137.01, 131.07, 128.58, 127.46, 125.86, 123.24, 121.73, 120.78, 120.10, 119.09, 118.86, 116.56, 115.37, 114.78, 112.91, 111.47, 99.99, 56.11, 32.59, 11.77. Anal. Calculated for C_28_H_24_N_2_O_5_, Mol. Wt.: 468.50: C, 71.78; H, 5.16; N, 5.98; Found: C, 71.92; H, 5.07; N, 6.16.

(**7q**) 3-acetyl-2-methyl-4-(5-methyl-furan-2-yl)-1H-pyrrolo[3,2-c]quinoline mp 118–119 °C, spectroscopical data in accordance with literature [5].

(**8r**) 2-acetyl-3-methyl-5-(5-methyl-furan-2-yl)-5,6-dihydro-pyrrolo[1,2-c]quinazoline.

Recrystallization from EtOH yielded 65% of orange crystals; mp: 152–154 °C; IR: 3297, 2924, 1641, 1613, 1593, 1511, 1325, 1242, 1190, 1070, 947, 797, 747, cm^−1^; ^1^H NMR (200 MHz, CDCl_3_) δ 7.46 (d, *J* = 7.3Hz, 1H), 7.05–6.90 (m, 2H), 6.85 (d, *J* = 7.8 Hz, 1H), 6.72 (t, *J* = 7.3 Hz, 1H), 6.63 (s, 1H), 5.87 (s, 1H), 5.68 (s, 1H), 2.55 (s, 3H), 2.37 (s, 3H), 2.13 (s, 3H); ^13^C NMR (50 MHz, DMSO) ppm 194.11, 151.69, 151.18, 138.81, 132.45, 127.21, 125.87, 121.91, 121.47, 118.79, 115.87, 115.03, 107.74, 106.33, 103.78, 59.98, 28.58, 13.24, 10.79. Anal. Calculated for C_19_H_18_N_2_O_2_, Mol. Wt.: 306.36: C, 74.49; H, 5.92; N, 9.14; Found: C, 74.61; H, 5.95; N, 9.29.

All spectra are available in Supplementary Material Figures S12–S65.

### 2.2. Biology

#### 2.2.1. Cell Cultures

MCF7 (epithelial cells isolated from the breast tissue—ATCC) and HeLa (epithelial cells isolated from a cervical carcinoma) cells line, were cultured in Eagle’s minimum essential medium (MEM) supplemented with heat-deactivated (56 °C, 30 min) 10% FBS, streptomycin and penicillin, 1% nonessential amino acids and 2 mM L-glutamine (all from Euroclone), as adherent monolayers in a humidified ambient (5% air CO_2_) at 37 °C.

#### 2.2.2. Cell Treatments

Reached confluence, MCF7 and HeLa cells were treated with four different concentrations of **7p** (1, 3, 10, and 30 μM) for 24 h. At least three replicates were performed for each experiment.

#### 2.2.3. Clonogenic Assay

After treatments, the cells were harvested and, to form colonies in 1–3 weeks, they were seeded at a density of 50 cells/cm^2^, in a six-well plate. For the entire period necessary for the formation of colonies, the cells were maintained in fresh medium at 37 °C in an atmosphere containing 5% CO_2_. Then plates were fixed in 100% methanol and later stained with 0.5% crystal violet in 20% methanol. Thus, the plates were air-dried. Colonies were considered as cell aggregates with at least 50 cells. The colonies were photographed using a digital camera and counted with countPHICS (count and Plot Histograms of Colony Size) software, a macro written for ImageJ (version 1.49v, Java 1.8.0_45, Wayen Rasband, U.S. National Institutes of Health, Bethesda, MD, USA; website: http://rsb.info.nih.gov/ij/download.html (accessed on 1 June 2025)) [16]. Data were expressed as colonies number.

#### 2.2.4. Wound Healing Test

MCF-7 and HeLa cells were grown on a 6-well plate until confluence. In each well, three circular wounds were performed using a 200 µL pipette tip to measure cell migration following the creation of a wound in the confluent cell monolayer. To remove debris, wells were washed with PBS, and after one hour, cells were treated with **7p** (1, 3, 10 and 30 μM). Images were obtained using a digital camera connected to an inverted phase-contrast optical microscope (Axiocam 208 color, Zeiss, Boston, MA, USA) at 0, 24, 48, and 72 h after wound creation. ImageJ programme was used to measure the area of the remaining wound size and wound closure rates. The results were expressed as a percentage of area reduction at time points 24, 48, and 72 h compared to time point 0 h. Three replicates were performed for each experiment.

#### 2.2.5. Statistical Analysis

Determination of statistical significance was performed using analysis of variance (ANOVA) corrected with Fisher’s test. A value of *p* < 0.05 was regarded as statistically significant.

#### 2.2.6. MTS Assays

Cell viability was evaluated by CellTiter 96 Aqueous One Solution Cell Proliferation Assay (PROMEGA, Madison, WI, USA) according to the manufacturer’s instructions. The cells were plated in 96-well plates and were treated for 24 h with different concentrations of stimuli. The absorbance was read at 490 nm on the Microplate reader (Spark Tecan Plate Reader (Tecan Group Ltd., Männedorf, Switzerland). Results were expressed as a percentage relative to the not-treated (NT) sample. The experiments were repeated three times, and the data were represented as the mean of quadruplicate wells ± SD.

### 2.3. Computational Studies

The preparation of ligands and proteins for in silico studies was carried out following the subsequent rigorous, detailed procedures.

#### 2.3.1. Ligand Preparation

The ligands were prepared using the LigPrep tool (Release 2017-1; Schrödinger LLC, New York, NY, USA) within the Schrödinger Maestro Suite [17]. Each ligand underwent exhaustive tautomer and stereoisomer generation at a pH of 7.0 ± 0.4, employing default settings and the Epik ionization method [18]. Following this, the Optimized Potentials for Liquid Simulations (OPLS 2005) force field was employed to minimize the energy status of the ligands [19].

#### 2.3.2. Protein Preparation

Crystal structures of Tubulin, Topoisomerase II, HSP90, IGF1R, PIK3CA, CDK6, HDAC2, and HDAC8 (PDB codes 1SA0 [20], 3QX3 [21], 2FWY [22], 2OJ9 [23], 8EXL [24], 5L27 [25], 4LXZ [26], and 3SFF [27], respectively) were retrieved from the Protein Data Bank [28,29]. Using the Protein Preparation Wizard within the Schrödinger software suite, default settings were applied to prepare these structures [30]. The protocol included the assignment of bond orders, retention of Het groups, removal of crystallographic water molecules, and adjustment of protonation states of heteroatoms using Epik, at a biologically relevant pH (7.0 ± 0.4). Hydrogen bond networks were then optimized, followed by a restrained energy minimization using the OPLS 2005 force field, with a root mean square deviation (RMSD) cutoff for atomic displacement set to 0.3 Å [19].

#### 2.3.3. Docking Validation

Molecular docking studies were carried out using the Glide module within the Schrödinger Suite. Receptor grids were generated by centring the grid boxes on the co-crystallized ligands. The enclosing grid box was defined as a cube with side lengths of 26 Å and a grid point spacing of 2.89 Å. Employing the Extra Precision (XP) mode as the scoring function, 3D conformers were docked into the receptor model. A post-docking minimization step was executed for each ligand conformer, generating a maximum of two docking poses and a total of five poses per ligand conformer. Notably, the docking protocol successfully redocked the original ligands within the receptor-binding pockets with an RMSD  < 0.51 Å. The Induced Fit Docking (IFD) simulation was conducted using the Schrödinger IFD protocol, a highly accurate and reliable method that accounts for the flexibility of both ligand and receptor. The resulting IFD poses were ranked based on the IFD score, calculated as: IFD score = 1.0 × Glide Gscore + 0.05 × Prime Energy, which integrates both protein–ligand interaction energy and the overall system energy.

The IFD scores are reported as negative values, with more negative scores indicating more favourable predicted binding affinities. The IFD score integrates both the ligand binding energy and the energetic contribution associated with receptor side-chain rearrangements that occur upon ligand accommodation in the binding site.

## 3. Results

### 3.1. Chemistry

The synthesis of the 1,4-substituted-pyrrole[3,2-b]quinoline derivatives (PQs) was carried out via an optimized chemical pathway, previously developed by our research group [4,5]. This chemical approach involves commercially available aryl or heteroaryl amines that undergo a formal Paal-Knorr cyclo-condensation with an easily two steps accessible o-nitrophenyl-triketone **1**. The reaction affords various R^1^ N-linked o-nitro-phenylpyrroles **2a**–**c** (Scheme 1).

Catalytic hydrogenation over Pd/C led to the key intermediates o-amino-phenylpyrroles, **3a**–**c**. Subsequent treatment of these latter with substituted aromatic or hetero-aromatic aldehydes (R^2^) by heating in DMF with 15% p-TSA, permitted the isolation of diversely 1-4 functionalized PQs **7d**–**q**.

The reaction proceeds via a related Pictet-Spengler mechanism involving a sequential intramolecular hetero annulation of internal imines, followed by a spontaneous tricycle aromatization. This versatile metal-free synthetic pathway enables the isolation of a library of new PQs featuring modifications at two or three chemical diversity points (1-3-4 substitution) and allowing a fine-tuning of their biological and physiochemical properties.

Our investigations focused on structural modifications of the promising derivative **7d** (formerly **4g** [4]), which previously showed activity against five tumour cell lines. The goal was to further explore the structure–activity relationship (SAR) and gain deeper insights into the structurally crucial features decisive for the interaction with biological entities.

Herein, fourteen new PQ derivatives (**7e**–**p**) were synthesized following the reaction conditions as reported previously by us and adapting the reaction conditions to the new reactants (Scheme 1).

All the new compounds and related intermediates have been characterized by means of spectroscopic data, ^1^H NMR, ^13^C NMR, Dept including IR, GC-MS fragmentation pattern, and elemental analysis. All the spectral data agreed with the proposed structures.

According to previously reported data [4], the structures of the cyclised derivatives **7d**–**q** were readily confirmed by comparison with their amino precursors **3a**–**c**. Indeed, the disappearance of the amino proton signal in the range d 3.9–5.6 ppm, together with the contextual loss of the pyrrole proton signal around d 6.6 ppm, clearly confirmed the success of the ring closure. Under these reaction conditions, no signals corresponding to intermediates **4** or **5** were detected. Additionally, the absence of both the NH and -CH proton signals (as those observed for species **6** shown in the square brackets), excluded the presence of the di-hydro-pyrroloquinoline form, indicating that only the fully aromatic PQ ring system has been obtained.

**Scheme 2:**
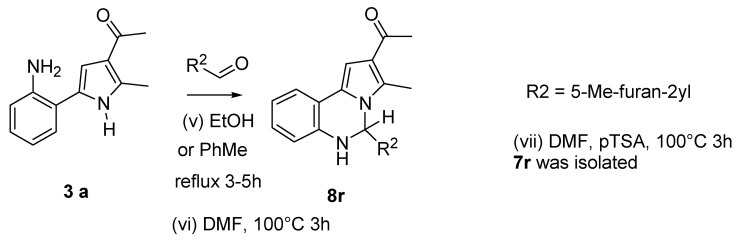
Alternative cyclisation pathway was observed under EtOH or PhMe as a solvent reaction.

Interestingly, when hetero-aromatic aldehydes were used under the same experimental reaction conditions (DMF,15% p-TSA) and R^1^ = H, a competitive cyclization pathway also took place. This was the case with the 5-Me-furan-aldehyde, wherein the di-hydro quinazoline angular tricycle **8r** was also isolated to a minor extent (Scheme 2).

It is worth noting that when the reaction was carried out in EtOH and R1 = H, the competing cyclization pathway took place consistently alongside the formation of the PQ framework. In this case, another tricyclic pyrrole containing (2-Acetyl-3-methyl-5-R^2^-5,6-dihydro-pyrrolo[1,2-c]quinazoline **8r** of biological interest [31] was also formed. Under these conditions, the use of EtOH as a solvent appeared to divert the reaction from the previously regiospecific profile [5]. Moreover, the resulting di-hydro species so formed do not spontaneously undergo complete aromatization (air presence), as observed for the formation of the fully aromatic PQ ring system.

By using hetero-aromatic aldehydes such as 5-Me-furan-aldehyde under the identical experimental reaction conditions as specified in Scheme 1, the alternative tricycle **8r** was also isolated.

Thus, a different regioselective chemical pathway is observed depending on the nature of the aldehyde (aromatic and hetero-aromatic) and the nature of the solvent in use.

### 3.2. Biological Studies

As recently reported by us, derivative **7d** bearing the benzo[1-3]dioxol-5yl (also named piperonyl) group at R^2^ position of the PQ skeleton, and R^1^ = H, revealed as the most effective compound among the first PQ series [4]. It exhibited significant antiproliferative activity (GI_50_ ≈ 10 μM) towards five tumour cell lines belonging to five different cancer panels (Leukemia, CNS, Melanoma, Renal, Breast cancers).

All these achievements have sparked our interest to pursue further investigations in order to gain additional insights into the attracting activity profile shown by **7d**. Pointing to the activity optimization, a new series of PQ derivatives was designed, synthesized, and preliminary tested as antiproliferative by SRB-based colorimetric NCI testing and additional MTS.

The selected ones, together with the related “one dose mean graph” (Supplementary Material Figures S1–S9), are listed below.

#### 3.2.1. NCI Antiproliferative Assays

The NCI assay is a well-consolidated assay protocol in the research of anticancer compounds, which provides antiproliferative data against at least 60 human cancer cell lines. The new compounds of type 7 were assayed in the one-dose screen [32]. As shown in Table 1, the rotation of the benzodioxole moiety from the 5-yl to 4-yl (**7d** vs. **7e**) disrupted the activity in almost all the cell lines, except for the UO31 cells, where the activity was retained. Instead, the dioxole methylene ring opening, as in **7f**, permitted a near recovery of the **7d** activity.

The antiproliferative activity is referred to at a concentration of 10 μM (see NCI protocol [27]). References: Doxorubicin and Podophyllotoxin are listed in Supplementary Materials Figures S10 and S11.

To better rationalize the structure–activity relationships within this series, compounds **7d** and **7f** were profiled using SwissADME [33]. Both analogues show highly comparable global physicochemical properties, with consensus logP values of 3.77 (**7d**) and 3.52 (**7f**), and similar solubility classes (“moderately soluble” by ESOL and Ali models). The calculated polar surface areas (64.21 Å^2^ for **7d** and 75.21 Å^2^ for **7f**) and H-bonding patterns indicate only a modest increase in polarity upon ring opening. Importantly, both compounds retain high predicted GI absorption, no violations of Lipinski, Ghose, Veber, Egan, or Muegge filters, no PAINS, and display overlapping CYP inhibition profiles.

Overall, these data indicate that the restoration of an activity level in **7f** comparable to that of **7d** is consistent with their closely matched ADME profiles, as no major differences in lipophilicity, solubility, or permeability are observed between the two compounds. This suggests that the ring-opened phenolic scaffold of **7f** does not introduce detrimental physicochemical liabilities. The ADME profiles of compounds **7d** and **7f** are summarized in Table 2, while the full SwissADME output is provided in Supplementary Table S1.

A previous series of PQ derivatives tested by NCI demonstrated that, among all the substitutions made on position 4 of the PQ skeleton, the benzodioxole-5yl group resulted as the most performant in the low micromolar range (10 μM), acting against 5 tumour cell lines belonging to diverse tumour panels. Based on these promising outcomes, we started deeper investigations by focusing on modifications mainly on the benzodioxole portion, with the aim of better understanding the selectivity and potency profile. We focused on the benzodioxole group because it was the only group (located on position 4 of the PQ skeleton) able to act significantly against five tumour cell lines.

#### 3.2.2. MTS Antiproliferative Assays

**Figure 2:**
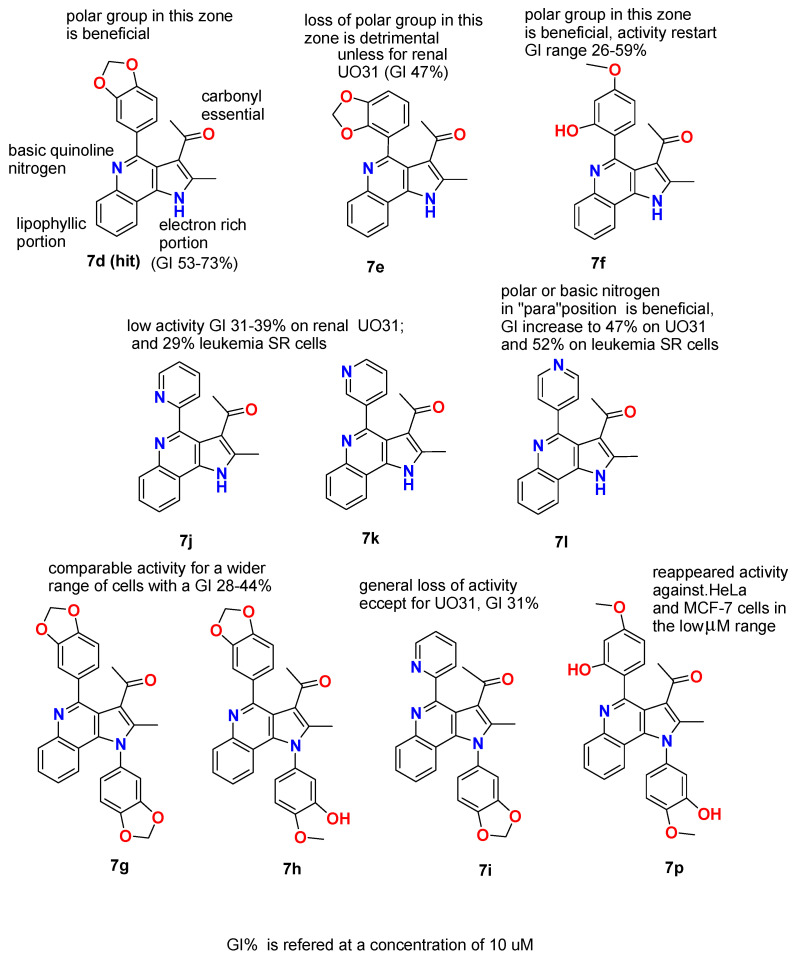
Simplified overview of the SAR studies. GI% refers to inhibition percent at 10 μM (NCI data). Red color refers to oxygen atoms, blue to Nitrogens.

From the eight derivatives selected by NCI, only a few were of interest as antiproliferative agents. This is the case of derivative **7f** that exhibited a similar pattern of selectivity and potency to **7d**. In our optimization efforts, while remaining moderate, from the NCI in vitro data so far obtained, the leukemia cancer panel and renal one retained sensitivity toward most of the derivatives. Better interesting achievements appeared with the MTS testing performed on compounds discarded by NCI selection, such as **7m**–**p**. Indeed, these latter, together with the already NCI tested **7g**–**i**, were also submitted to in vitro MTS assays. Thus, a comparative investigation was performed as reported in Figure 2 against an additional five tumour cell lines not included in the NCI panel (CaCo2; 16HBE; HeLa; H292; LAN5) and one included (MCF-7). From these antiproliferative tests, derivative **7p** was the most effective, especially in MCF-7 and HeLa cells (Table 3). Therefore, it was submitted to deeper biological assays such as clonogenic assays and scratch tests. Interestingly, derivative **7m,** bearing the two main functional groups of crucial significance in podophyllotoxin structure, namely the 1-(benzodioxole-5yl) and the 4-(3,4,5-trimetoxy-phenyl-1yl), was also tested as an apoptotic inducer.

#### 3.2.3. Clonogenic Assay on HeLa and MCF-7 Cells

**Figure 3:**
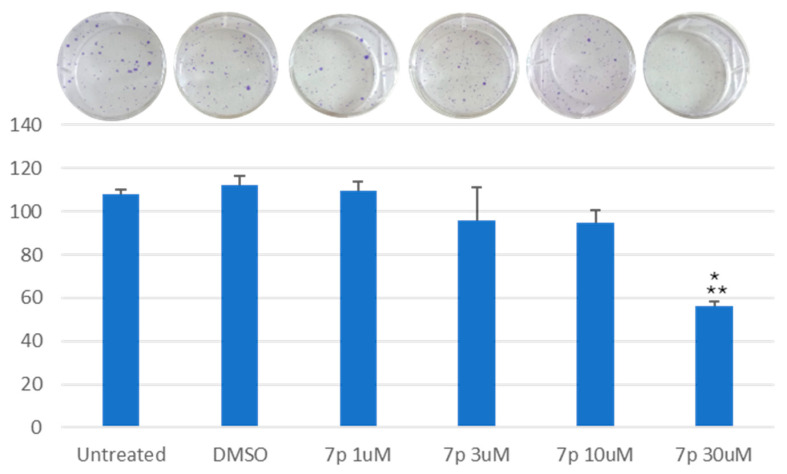
Effect of **7p** on colony formation ability in the HeLa cell line. HeLa cell line was cultured for 24 h with **7p** (1, 3, 10, and 30 μM) and colony-forming ability was assessed by clonogenic assay. Results are expressed as mean ± SD (*n* = 3). The comparison between different experimental conditions was evaluated by ANOVA corrected with Fisher’s test. * *p* < 0.05 was accepted as statistically significant. “*” is vs. Untreated; “**” is vs. DMSO.

**Figure 4:**
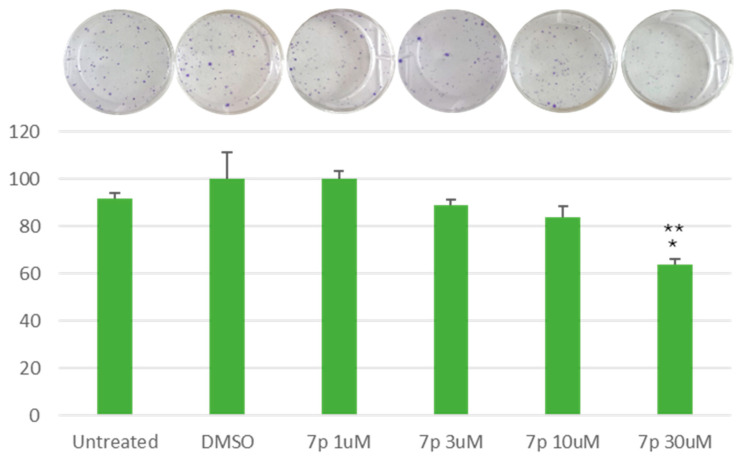
Effect of **7p** on colony formation ability in the MCF7 cell line. MCF7 cell line was cultured for 24 h with **7p** (1, 3, 10, and 30 μM), and colony-forming ability was assessed by clonogenic assay. Results are expressed as mean ± SD (*n* = 3). The comparison between different experimental conditions was evaluated by ANOVA corrected with Fisher’s test. * *p* < 0.05 was accepted as statistically significant. “*” is vs. Untreated; “**” is vs. DMSO. In the above performed clonogenic tests, either in MCF-7 and HeLa cells, a significant reduction in colony number was observed upon exposure to **7p** at 30 μM concentration.

The appreciable selectivity exhibited by derivative **7p** against MCF-7 and HeLa cells (Figure 3 and Figure 4) prompted us to further investigate the behaviour of colony formation ability. It is well known that this type of cell test is carried out to compare the ability of cells to survive the injury caused by the test agent. The retention of the proliferative capacity and formation of progeny colonies is evaluated by observing the aptitude of a single cell to grow and restart a colony.

The use of the crystal violet assay permits to visualization of the effect of cell survival or growth inhibition by test molecules [34]. The procedure tests every cell in the population for its ability to undergo unlimited divisions. It is recognized that only a fraction of seeded cells retains the capacity to produce colonies [35,36].

#### 3.2.4. Scratch Test on HeLa and MCF-7 Cells

To further explore the migration inhibition properties of the after the treatment with derivative **7p**, the cell invasion capability was evaluated on HeLa and MCF-7 cells. The scratch-wound assay is commonly used to measure basic cell migration parameters such as speed, persistence, and polarity [37], and it represents a useful method to follow cell proliferation and migration, giving information on the mechanisms of cancer growth and metastasis.

**Figure 5:**
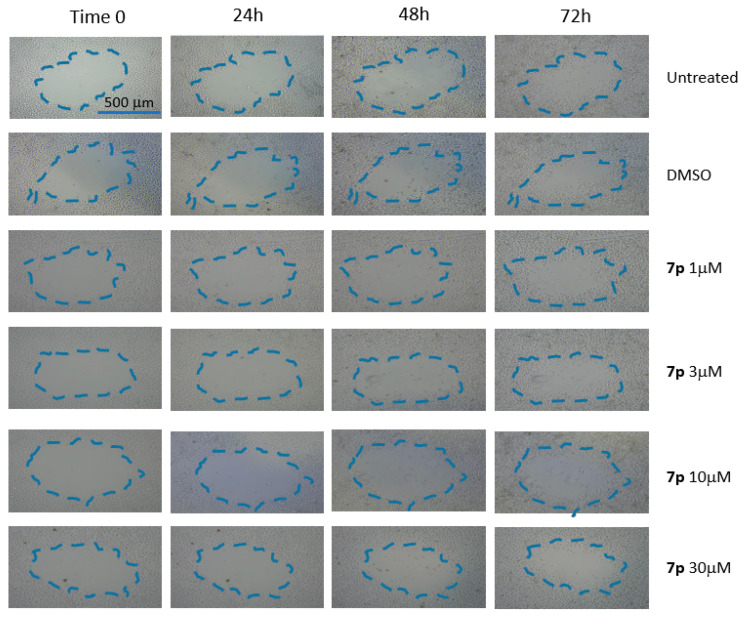
Scratch cell assay (HeLa) after treatment of **7p** at different concentrations at 24, 48, and 72 h. All experiments were performed in triplicate. The magnification is 4×.

**Figure 6:**
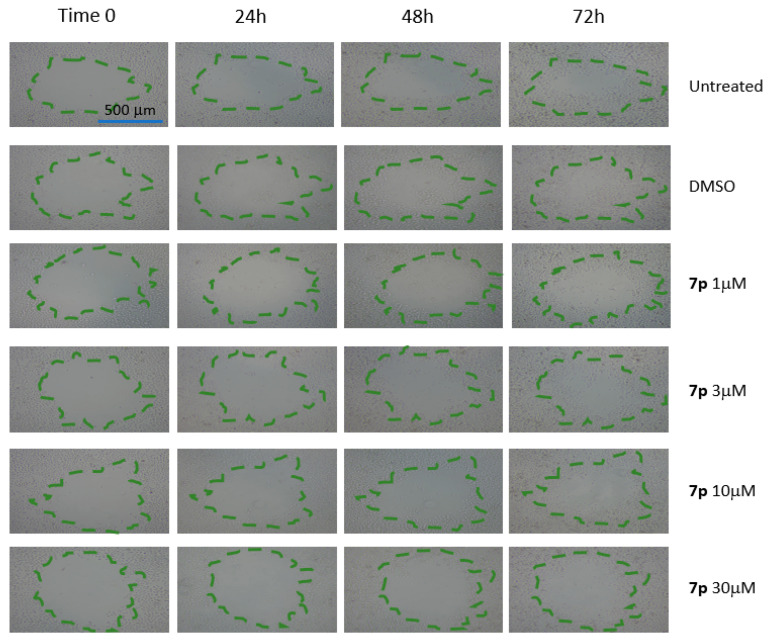
Scratch cell assay MCF-7 after treatment with **7p** at different concentrations at 24, 48, and 72 h. All experiments were performed in triplicate. The magnification is 4×.

**Figure 7:**
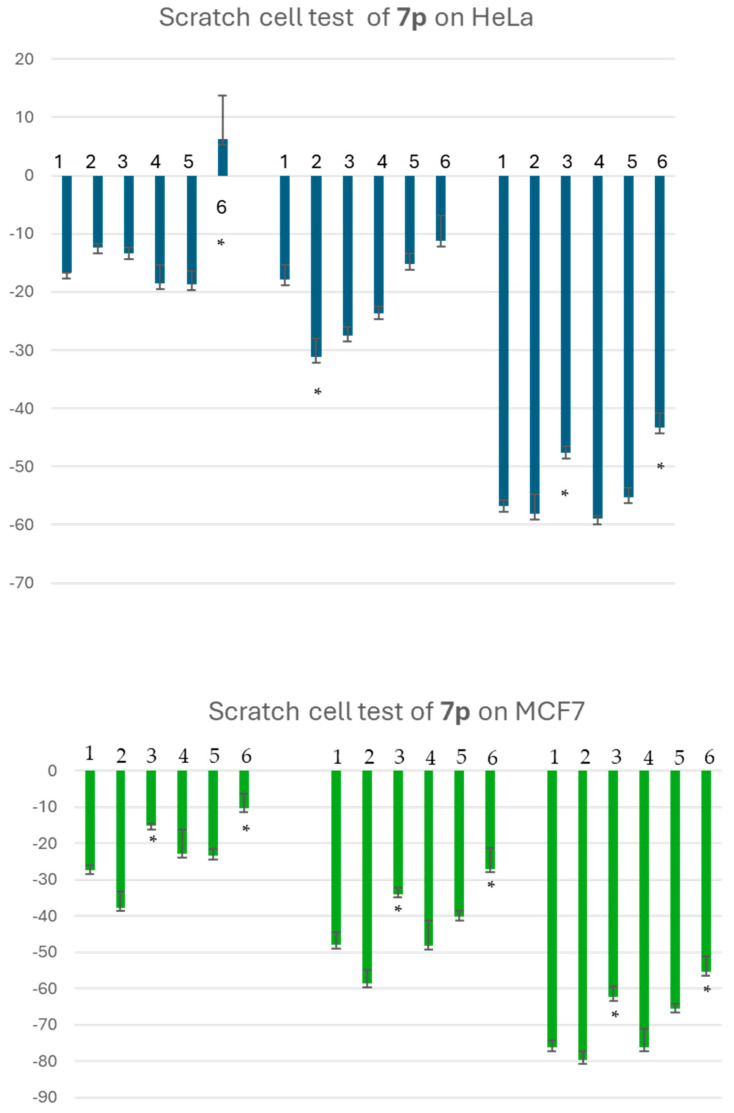
Comparison of the scratch cell test between HeLa and MCF-7 cells. Effect after treatment of **7p** at different concentrations (24, 48, and 72 h). HeLa and MCF7 cells were seeded (separately) on a 6-well plate and cultured to confluence. A scratch was made in each well using a 200-μL pipette tip. Cells were stimulated with **7p** for 24, 48, and 72 h. The results were expressed as a percentage of area reduction at 24 h, 48 h, and 72 h, compared to time point 0 h. The comparison between different experimental conditions was evaluated by ANOVA corrected with Fisher’s test. * *p* < 0.05 was accepted as statistically significant.

In more detail, the surface area formed after the scratch showed a dose and time-dependent percentage of area reduction (Figure 5 and Figure 6). The effect of derivative **7p** is more evident on HeLa with respect to MCF-7 cells (Figure 7). Thus, these results confirm the inhibition of cell mobility (invasion) of the compound, which is associated with a potential role as an anti-angiogenic effect [38,39].

#### 3.2.5. Apoptosis Induction of Derivative **7m** on HeLa Cells

Molecules capable of selectively inducing apoptosis are of great interest in anticancer therapy. Numerous studies have shown that the loss of the ability of cells to undergo apoptosis represents a fundamental moment in neoplastic transformation and that many of the antitumour chemotherapeutic drugs act by modulating this biological process.

**Figure 8:**
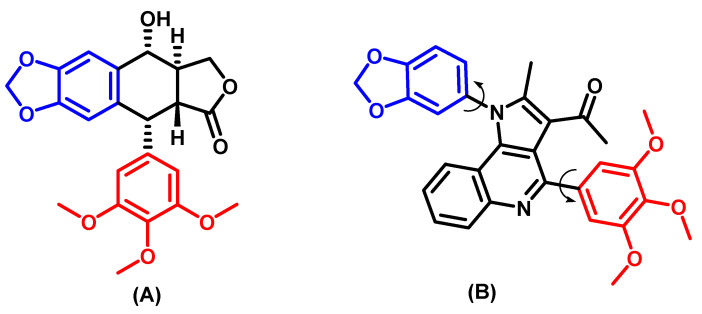
(**A**) Podophyllotoxin (left) (**B**) PQ skeleton containing two crucial moieties of Podophyllotoxin (red: trimethoxy-phenyl, blue: benzodioxole moiety). The two colors blue (benzodioxole) and red (trimethoxy-phenyl) refer to the two portions of podophyllotoxin that were inserted on the pyrrolo[3,2-c]quinoline scaffold.

**Figure 9:**
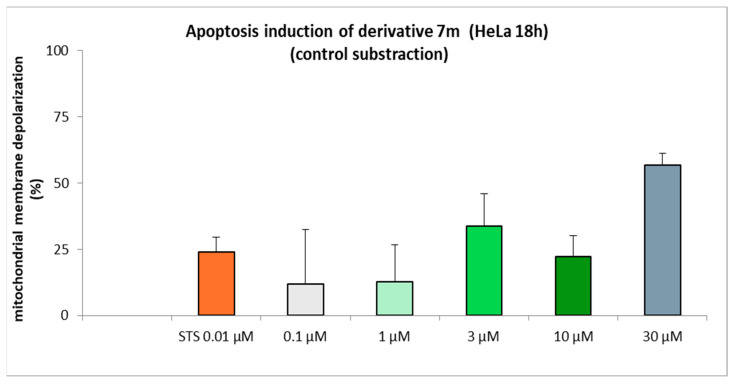
Apoptosis induction was evaluated with the TMRM-based test of derivative **7m** on HeLa (18 h). Staurosporine (STS) is used as a reference. Arrows refer to the possibility of the group to rotate along the bond axis.

Because of the particular structural analogy of derivative **7m** with podophyllotoxin (Figure 8), both containing the crucial trimethoxy phenyl group and the piperonyl one, we were particularly interested to evaluate the effect of the two crucial groups (freely rotating) in the entirely planar tricyclic PQ on the apoptosis induction. For this purpose, we followed the mitochondrial membrane depolarization protocol [40,41], whose results are reported in Figure 9. The assay was conducted according to previously reported procedures [42,43].

The various mechanisms through which these molecules induce apoptosis remain largely unknown. Some studies have indicated that the wild-type p53 tumour suppressor gene, the Fas/Fas binding system, and caspase activation may play an important role in drug-induced apoptosis [44,45].

### 3.3. In Silico Studies: Induced Fit Docking Investigations

The development of novel anticancer agents increasingly relies on a rational design approach that integrates experimental and computational methods to elucidate structure–activity relationships and identify potential molecular targets.

To gain deeper insights into their potential mode of action, computational docking studies can provide valuable information by predicting the binding affinity and interaction profiles of such compounds with biologically relevant protein targets. This in silico approach allows the identification of key protein–ligand interactions that may contribute to the observed antiproliferative effects, thereby complementing experimental findings and guiding further optimization of the chemical scaffold.

#### 3.3.1. Assessment of Podophyllotoxin-like Mechanisms of Compound **7m** Through Molecular Docking

To further investigate whether compound **7m** might share mechanistic features with podophyllotoxin, an in silico docking study was performed on two representative targets of this natural product, tubulin (PDB code: 1SA0 [20]) and topoisomerase II (PDB code: 3QX3 [21]). Table 4 presents IFD scores and docking scores for **7m** and reference ligands.

On tubulin, compound **7m** exhibited a docking score of −11.813 and an IFD score of −1753.29, values more favourable than those obtained for podophyllotoxin (−11.331/−1749.08) and the co-crystallized ligand (−11.152/−1749.12).

**Figure 10:**
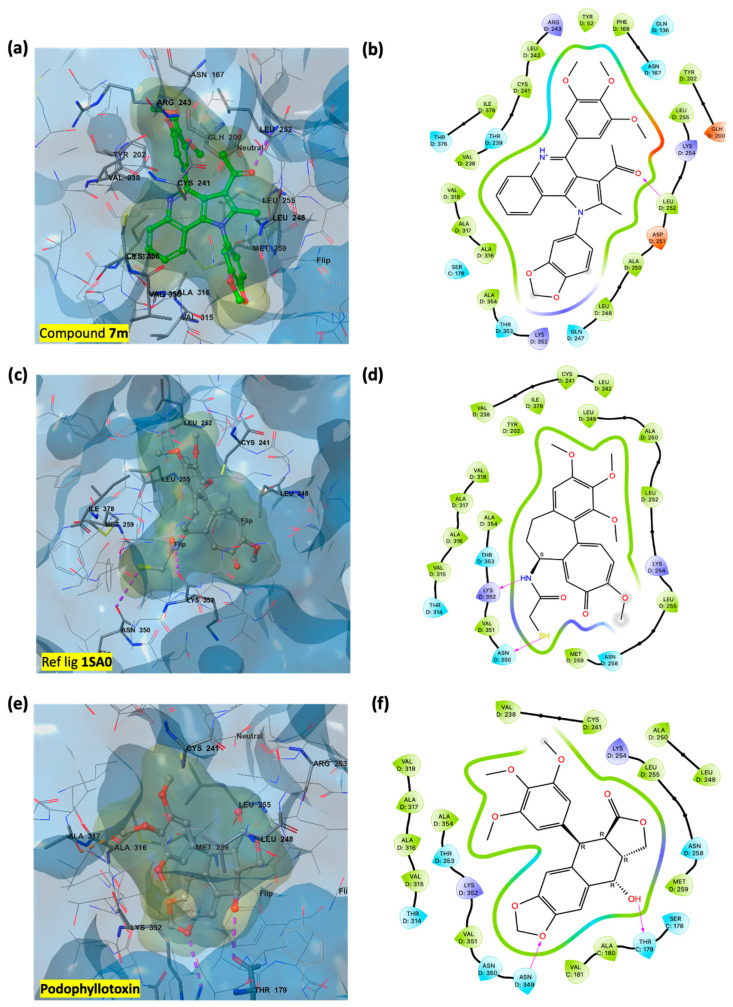
(**a**) 3D binding pose of compound **7m** in complex with tubulin (PDB code 1SA0); (**b**) ligand-protein interaction diagram for complex **7m**/tubulin; (**c**) 3D binding pose of ref lig (co-crystalized ligand) in complex with tubulin (PDB code 1SA0); (**d**) ligand-protein interaction diagram for complex ref lig (co-crystalized ligand)/tubulin; (**e**) 3D binding pose of podophyllotoxin in complex with tubulin (PDB code 1SA0); (**f**) ligand–protein interaction diagram for complex podophyllotoxin/tubulin. Protein and ref ligs carbon atoms are shown in grey, ligand carbon atoms in green, oxygen atoms in red, nitrogen atoms in blue, and sulfur atoms in yellow.

Figure 10a,c,e illustrates the 3D binding poses for compounds **7m**, the co-crystalized ligand, and podophyllotoxin, while the corresponding ligand-interaction diagrams are reported in Figure 10b,d,e. These results revealed that **7m** binds within the binding site of tubulin with a moderate predicted affinity, establishing itself thanks to residues such as Tyr^202^, Val^238^, Cys^241^, Arg^243^, Leu^248^, Leu^252^, Leu^255^, Met^259^, Val^315^, Ala^316^, Asn^350^, Lys^352^, Val^355^, Cys^356^, and Ile^378^, key amino acids also involved in the stabilization of the podophyllotoxin and Ref Lig (the co-crystalized ligand) complexes.

For topoisomerase II, compound **7m** displayed a docking score of −8.028 and an IFD score of −1686.15, slightly more favourable than those of the co-crystallized ligand etoposide (−7.552/−1685.51) and significantly better than podophyllotoxin (−6.232/−1681.10).

**Figure 11:**
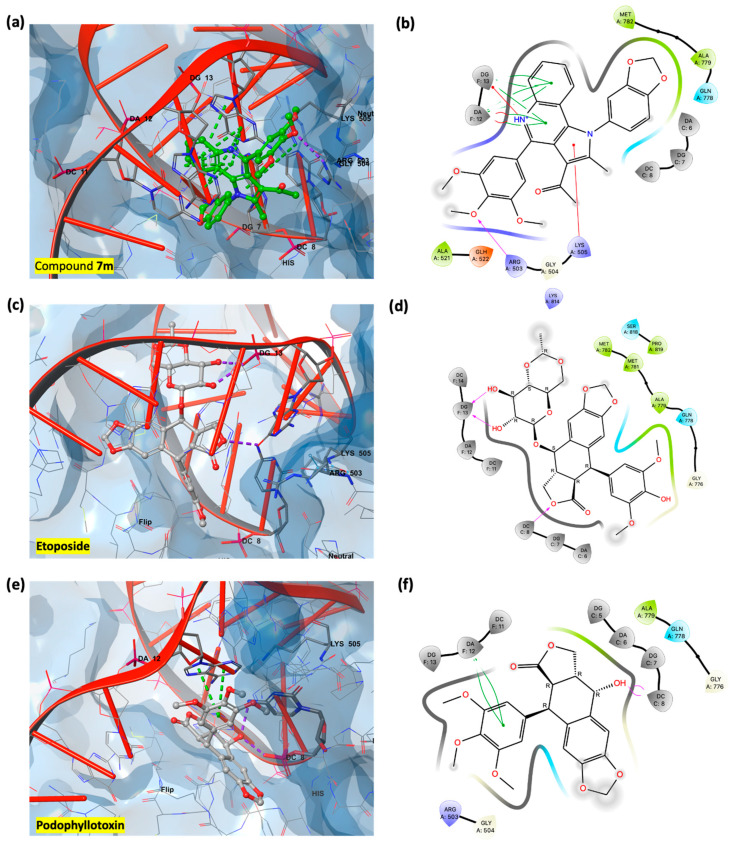
(**a**) A 3D binding pose of compound **7m** in complex with topoisomerase II (PDB code 3QX3); (**b**) ligand–protein interaction diagram for complex **7m**/topoisomerase II; (**c**) 3D binding pose of co-crystalized ligand (etoposide) in complex with topoisomerase II (PDB code 3QX3); (**d**) ligand-protein interaction diagram for complex co-crystalized ligand (etoposide)/topoisomerase II; (**e**) 3D binding pose of podophyllotoxin in complex with topoisomerase II (PDB code 3QX3); (**f**) ligand-protein interaction diagram for complex podophyllotoxin/topoisomerase II. Protein and ref ligs carbon atoms are shown in grey, ligand carbon atoms in green, oxygen atoms in red, nitrogen atoms in blue, and sulfur atoms in yellow.

Figure 11a,c,e shows the 3D binding poses of **7m**, co-crystalized ligand (etoposide), and podophyllotoxin, while the corresponding ligand-interaction diagrams are reported in Figure 11b,d,e. Our findings suggest that compound **7m** may establish a stable binding within the topoisomerase II catalytic pocket, supporting a possible contribution of topoisomerase inhibition to its antiproliferative activity. Specifically, **7m** forms interactions with key amino acids Arg^503^, Gly^504^, and Lys^505^, as well as with DNA bases DG7, DC8, DC11, DA12, and DG13, showing a higher number of contacts than both podophyllotoxin and etoposide.

Overall, the docking results and binding conformation obtained in both IFD studies support a mechanism of action comparable to that of podophyllotoxin, indicating that microtubule inhibition and topoisomerase II inhibition may contribute to the biological activity of compound **7m**.

#### 3.3.2. Rationalization of Antiproliferative Activity of PQ Derivatives Through Induced Fit Docking on Six Cancer-Associated Targets

To support and further rationalize the observed antiproliferative effects of the PQ derivatives, an in silico study was conducted by performing Induced Fit Docking (IFD) on a selected panel of six molecular targets (HSP90, IGF1R, PIK3CA, CDK6, HDAC2, and HDAC8, which PDB codes are: 2FWY [22], 2OJ9 [23], 8EXL [24], 5L27 [25], 4LXZ [26], and 3SFF [27], respectively) known to be commonly expressed in both MCF-7 and HeLa cancer cell lines [46,47,48,49,50]. These targets were chosen based on their well-documented overexpression and pivotal roles in proliferation, survival, and apoptosis pathways.

Specifically, HSP90 functions as a molecular chaperone stabilizing multiple oncogenic proteins; IGF1R and PIK3CA are central components of growth and survival signalling pathways frequently dysregulated in these cancer cells; CDK6 regulates cell cycle progression; and HDAC2 and HDAC8 modulate epigenetic regulation and gene expression linked to tumour proliferation and apoptosis [51,52,53,54,55,56,57]. Their inclusion in the docking study thus provides a biologically relevant framework for exploring the potential multitarget inhibitory effects of the PQ derivatives.

Derivatives **7d**–**q** and reference ligands were submitted to Schrödinger’s IFD workflow. In detail, to comprehensively evaluate ligand–target interactions, both standard docking and Induced Fit Docking (IFD) protocols were employed. Standard docking treats the receptor as rigid, allowing rapid screening of ligand binding, but may not fully account for conformational adjustments within the binding site. To address potential flexibility of the targets and better capture realistic binding modes, IFD was applied, permitting limited receptor flexibility in response to ligand binding. This combined approach balances computational efficiency with accuracy, providing reliable predictions of both binding poses and affinities for the investigated derivatives.

Significant binding affinities were observed for all the analyzed targets, key regulators involved in cell survival, proliferation, and apoptosis. IFD scores obtained for the PQ derivatives are expressed as negative values, reflecting the overall docking energy that accounts for both ligand–receptor interactions and conformational adaptations of the protein. Lower (more negative) IFD scores indicate a stronger predicted binding affinity.

Notably, compound **7o** showed excellent docking and IFD scores against PIK3CA (−10.417 and −2036.46, respectively), CDK6 (−10.381 and −568.00, respectively), and HDAC2 (−6.601 and −845.79, respectively). Similarly, compound **7i** bound strongly to HSP90 (docking score: −13.002; IFD score: −479.36), supporting its potential role in disrupting this chaperone, which is often overexpressed in tumours.

Moreover, derivative **7p**, which showed outstanding biological results in clonogenic and scratch assays, also demonstrated superior binding energies compared to reference compounds for HSP90 (docking score: −13.558; IFD score: −478.93), IGFR1 (docking score: −9.720; IFD score: −654.49), and HDAC2 (docking score: −6.678; IFD score: −844.96), and competitive results for CDK-6 (IFD score: −564.60), HDAC8 (IFD score: −742.76), and PIK3CA (IFD score: −2034.92), suggesting a possible synergistic mechanism via simultaneous inhibition of multiple pro-survival pathways.

The IFD Score, which integrates binding energy with receptor conformational changes, remained within expected ranges for favourable ligand-target interactions. Table 5 presents IFD scores and docking scores for all derivatives (**7d**–**q**) and reference ligands across the selected targets. These reference ligands are the co-crystallized compounds extracted from the PDB entries used in the docking studies for the selected targets.

**Figure 12:**
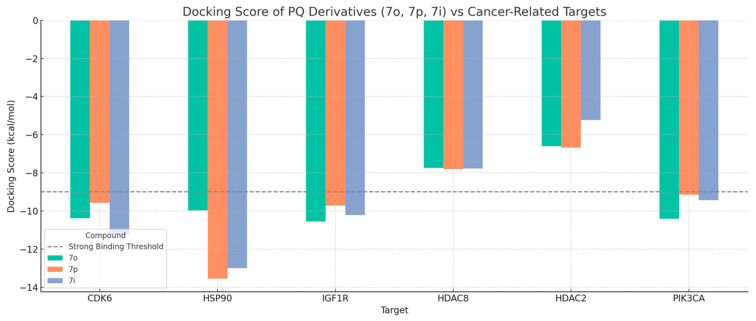
Docking scores of selected pyrrolo[3,2-c]quinoline (PQ) derivatives against key cancer-related targets. A docking score below −9 kcal/mol is generally considered indicative of strong binding affinity.

The observed correlation between lower IFD scores and higher antiproliferative activity, especially for **7p**, further supports the hypothesis that the anticancer activity may derive from multi-target interactions involving key oncogenic pathways. Figure 12 illustrates docking scores for these lead compounds, reinforcing the biological findings and suggesting that selected PQ derivatives act as potential multitarget inhibitors with antiproliferative and anti-migratory properties.

**Figure 13:**
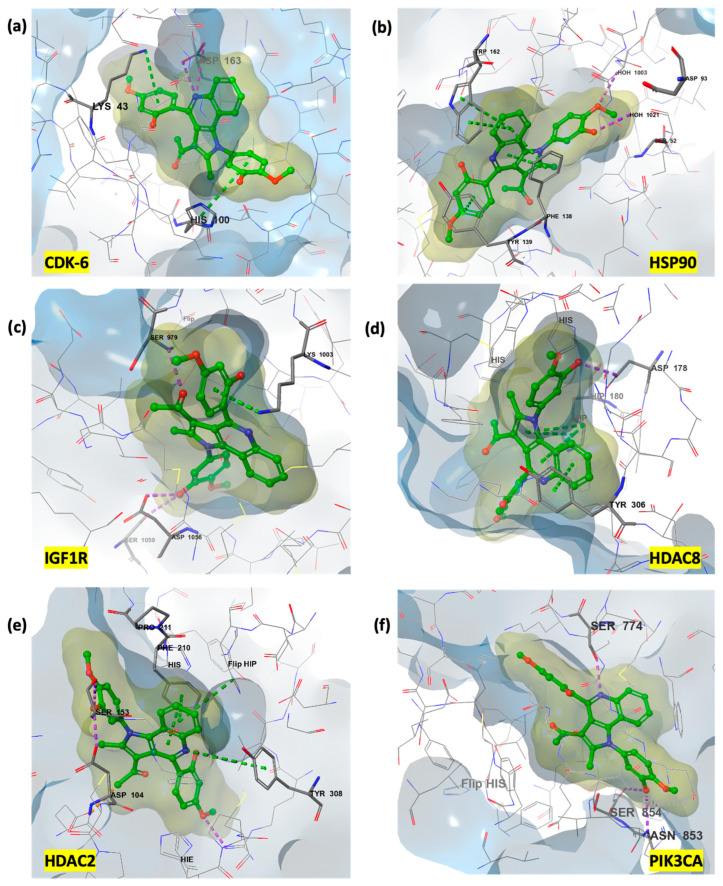
(**a**) A 3D binding pose of compound **7p** in complex with CDK-6 (PDB code 5L27); (**b**) 3D binding pose of compound **7p** in complex with HSP90 (PDB code 2FWY); (**c**) 3D binding pose of compound **7p** in complex with IGF1R (PDB code 2OJ9); (**d**) 3D binding pose of compound **7p** in complex with HDAC8 (PDB code 35FF); (**e**) 3D binding pose of compound **7p** in complex with HDAC2 (PDB code 4LXZ); (**f**) 3D binding pose of compound **7p** in complex with PIK3CA (PDB code 8EXL). Protein carbon atoms are shown in grey, ligands carbon atoms in green, oxygen atoms in red, nitrogen atoms in blue, and sulfur atoms in yellow.

**Figure 14:**
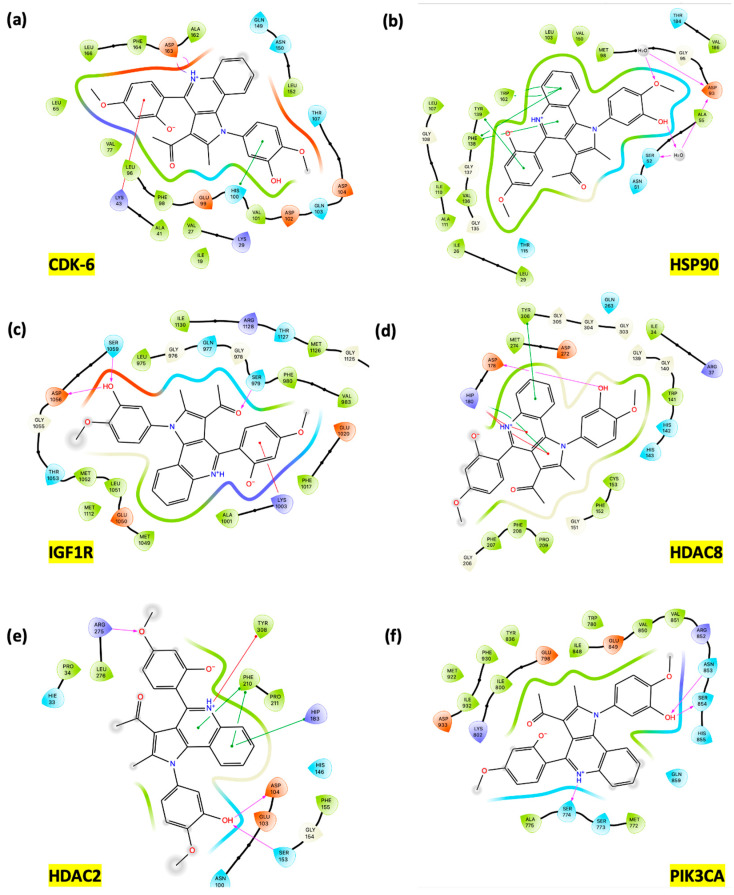
(**a**) A 2D protein–ligand interaction diagram of compound **7p** in complex with CDK-6 (PDB code 5L27); (**b**) 2D protein–ligand interaction diagram of compound **7p** in complex with HSP90 (PDB code 2FWY); (**c**) 2D protein–ligand interaction diagram of compound **7p** in complex with IGF1R (PDB code 2OJ9); (**d**) 2D protein–ligand interaction diagram of compound **7p** in complex with HDAC8 (PDB code 35FF); (**e**) 2D protein–ligand interaction diagram of compound **7p** in complex with HDAC2 (PDB code 4LXZ); (**f**) 2D protein–ligand interaction diagram of compound **7p** in complex with PIK3CA (PDB code 8EXL). Ligands carbon atoms are shown in grey, oxygen atoms in red, nitrogen atoms in blue, and sulfur atoms in yellow, while hydrogen bonds are labeled in purple and π–π stacking in green.

Finally, the best-ranked docking 3D poses of **7p** are depicted in Figure 13, while the corresponding 2D ligand–protein interaction diagrams are shown in Figure 14. Overall, the 3D and 2D representations confirm that compound **7p** is able to adapt its conformation efficiently to diverse protein environments while maintaining key polar and hydrophobic interactions.

Furthermore, 3D binding poses of compounds **7d**, **7h**, and **7i** within the **CDK6** active site are available in Supplementary Material Figure S66.

## 4. Discussion

The data still now obtained demonstrate that the angular tricycle pyrrolo[3-2,c]quinoline is a planar molecular scaffold of great interest for the development of new antiproliferative agents, and not only [58]. Starting from the outcomes furnished by the previous NCI screening, the SAR studies evidenced that the “piperonyl” (or benzodioxole) group in position 4 of the PQ scaffold was of crucial importance to impart antiproliferative activity in the 10 μM range. A note of relevance was the pattern of selectivity observed. Five tumour cell lines of diverse panels, including leukemia, CNS, renal, melanoma, and breast cancers [4], were targeted by this derivative, each one at the same dose. Further in-house additional MTS assays against the other six tumour cell lines (Figure 5), not included in the NCI panels, corroborated the observed antiproliferative activity accordingly, with better promising perspectives.

To enrich our previous SAR studies (Scheme 1), these attractive results engaged us to start a series of structural modifications, by acting mainly on moving, doubling, or modifying the crucial “piperonyl” functional group, synthesizing derivatives **7e**–**p**.

A first series of structural changes on **7d** e are shown in Figure 2 together with the pattern of antiproliferative activity (NCI “one dose mean graph”) at a concentration of 10 μM. Just a more angular rotation of the benzodioxole moiety in the PQ skeleton (moving from derivative **7d** to **7e**) led to a generalized decrease in activity, except for UO31 cells (renal cancer) in which the activity was maintained (GI% 50). Modifying the 1-benzodioxole moiety (**7d** to **7f**), namely “opening” the methylene dioxole portion, the activity reappears with a nearly identical pattern of selectivity of **7d**, suggesting a formal bio-isoterism between the two groups.

By doubling the benzodioxole moiety, in positions 1 and 4 of the PQ skeleton (**7g**), moderately leukemia and renal tumour cells retain sensitivity (GI_50_ 30%). Disrupting the methylene of the 1-benzodioxole moiety (**7h**), the trend remains almost unchanged with respect **7g**. Instead, replacing the 4-benzodioxole with a pyridin-2-yl group (**7i**), the antiproliferative activity flopped down consistently (Table 1).

Introducing a pyridin-2yl group in place of the benzodioxole of derivative **7d** (Table 1), we note a loss of activity. Interestingly, we observe a moderate increase in activity by distancing the nitrogen atoms. A longer distance between the two nitrogen (quinoline and pyridin) reveals a beneficial effect for the activity, favouring consistently and selectively the leukemia (SR) and the renal tumour cells (UO31) as for derivative **7l**, while an irrelevant effect for all the other tumour cell lines was noticed.

From a careful analysis of all the NCI one-dose mean graphs, the emerging pattern of selectivity and potency could suggest useful information on the probable mechanism of action. In our case, a few derivatives exhibited a similar selectivity pattern, often targeting the same tumour cell lines, differing only in efficacy. This peculiar behaviour could suggest a mechanism of action involving a common signalling pathway. This is the case of some derivatives of the PQ skeleton investigated until now. For example, comparing the trends of several derivatives, punctually the same tumour cell lines are involved (leukemia, melanoma, renal cancer) or in a particular case, only an entire panel is selectively concerned (ex. **7g**, **7h** targeting leukemia).

The additional MTS assays evidenced that derivatives **7g** and **7h** behave almost similarly in all the cell lines, except for H292. Markedly, the identical trend is observed in the NCI screen, where the leukemia panel was targeted moderately. Thus, the structural modification on the benzodioxole methylene (ring opening) does not change the antiproliferative profile (supporting and confirming once again, a formal bioisosterism between the piperonil group and the piperonyl dioxole cleaved group as above stated). The same pattern occurs for derivatives **7i** and **7n**.

Among all the tested compounds, derivative **7p** showed particular efficacy towards MCF-7 cells, inducing a total growth inhibition (TgI) below 10 μM. The two-ring opening of the 1-4-dioxole moieties improves the activity with respect to the unmodified benzodioxole groups (**7g**) selectively. Thus, a derivative **7p** (discarded by NCI) was, by our MTS assays, identified as a valuable hit acting with IC_50_ at the lowest μM concentration.

Except for CNS H292 cells, the two modified benzodioxole portions of **7p** derivative (grey line) are more sensitive against MCF-7 and HeLa cells (breast and ovarian cancer cells). Therefore, these two tumour cell lines were selected for the clonogenic and scratch cell test.

In the wound healing assay (Figure 7, Figure 8 and Figure 9), the limited variation in cell wound area with respect to the control demonstrates that derivative **7p** causes a significant inhibition of cell mobility on both cancer cell lines.

Because the assay is carried out either in a time and dose-dependent fashion, the results indicate a clear and significant inhibition of cell migratory effect. Moving to the clonogenic test (Figure 5 and Figure 6), where the treatment of **7p** reduces the colony formation (aptitude of the cell to restart in proliferation with lower aggressivity), was observed for the two cancer cell lines (MCF-7 and HeLa).

To support these findings, in silico studies were performed using Induced Fit Docking (IFD) against six cancer-related targets overexpressed in both cell lines. Remarkably, compound **7p** exhibited strong binding affinity towards HSP90 (IFDScore: −478.93), IGF1R (−654.49), and HDAC2 (−844.96), key proteins involved in cellular stress response, survival, and proliferation. The IFD results also showed favourable interactions with CDK6 (−564.6), HDAC8 (−742.76), and PIK3CA (−2034.92), suggesting a multi-target mechanism that may account for its antiproliferative, anti-migratory, and colony-reducing properties observed in vitro.

By matching the antiproliferative activity of derivative **7p** with its computational target profile, the tendency of a single cell to form colonies, and the reduced cell mobility, data indicate that all these effects account for its anticancer potential.

## 5. Conclusions

The investigation efforts so far performed testify how the PQ framework could be particularly versatile to impart activity, only with substitution at the 1-4 position. In our hands, once the benzodioxole group (SAR studies) was identified as crucial to address a significant antiproliferative activity in five diverse panels of tumour cells, suggesting a common signalling pathway. Thus, even if some derivatives are not particularly cytotoxic to exploit as antiproliferative, they retain other interesting properties, such as the antimetastatic ones, as detected with the scratch test. Rationally, the versatility of functionalities that could be introduced on the PQ skeleton, strengthened by the synthetic approach followed here, could lead to a large number of new compounds to be evaluated towards many biological effects in accordance with anticancer progression.

Importantly, the integration of in silico docking results further supports the biological findings. Compound **7p**, in particular, displayed strong affinity for key cancer-related targets such as HSP90, IGF1R, HDAC2, CDK6, HDAC8, and PIK3CA, all of which are involved in tumour proliferation, survival, and metastasis. These computational results underline a multi-target inhibitory profile, which likely contributes to the broad anticancer activity observed in vitro and suggests potential for rational polypharmacology.

The selectivity patterns observed for certain derivatives may be associated with the modulation of specific signalling pathways. Docking studies revealed strong interactions with CDK6, HDAC2, and HDAC8, which are key regulators of cell cycle progression and apoptosis. Additionally, interactions with HSP90 and PIK3CA suggest potential effects on growth and survival signalling pathways. Together, these results indicate that the observed selectivity could be related to coordinated modulation of cell cycle and apoptosis-related pathways, providing a biologically plausible rationale consistent with the in silico findings.

These preliminary data allow us to lay the foundations for the design of new derivatives and the optimization of molecules against a variety of cancers, depending on the appropriate choice of selected functionalization. Further biological studies are needed to gain further insights into the mechanism of action and will allow us to better understand the next investigative steps on tailored cellular targets. Attempts to enhance the activity have instead led to interesting aspects of modulation of selectivity towards tumour cells and the appearance of an effect to improve anticancer efficacy.

Although in vivo and specific mechanistic studies are still needed, these proposed results encourage planning further developments.

## Figures and Tables

**Table 1:** Representative Antiproliferative values of NCI one dose screening. Values GI < 24% were discarded. For the entire panels of the one-dose mean graphs data made at 10 μM, see Supplementary Materials. G%: Cell growth percent at 10 μM compared to untreated cells. GI%: Cell growth inhibition at 10 μM compared to untreated cells (As NCI protocols).

** 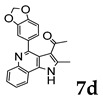 **			
**Panel**	**Cell line**	**G%**	**GI%**
Leukemia	SR	31.64	68.36
CNS	SNB75	46.47	53.53
Melanoma	MDA-MB-435	42.11	57.89
Renal	UO31	26.43	73.57
Breast	MCF-7	41.49	58.51
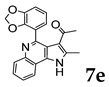			
**Panel**	**Cell line**	**G%**	**GI%**
Leukemia	SR	72.55	27.45
CNS	SNB75	75.72	24.38
Melanoma	MDA-MB-435	71.06	28.94
Renal	UO31	52.94	47.06
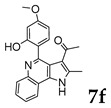			
**Panel**	**Cell line**	**G%**	**GI%**
Leukemia	SR	46.86	53.14
	K562	68.37	31.63
NSCLC	HOP62	69.72	30.28
	HOP92	69.45	30.55
CNS	SNB75	55.06	44.94
Melanoma	UACC62	54.36	45.64
Renal	UO31	40.72	59.28
Breast	MCF-7	73.48	26.52
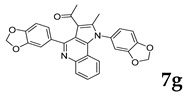			
**Panel**	**Cell line**	**G%**	**GI%**
Leukemia	SR	59.17	40.83
	K562	71.32	28.68
	CCRF-CEM	67.59	32.41
	MOLT-4	57.82	42.18
NSCLC	HOP62	69.82	30.18
CNS	SNB-75	65.24	34.76
Renal	A498	64.96	35.03
	UO31	55.38	44.62
Breast	T-47D	69.70	30.30
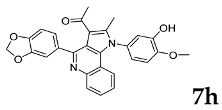			
**Cancer Panel**	**Cell line**	**G%**	**GI%**
Leukemia	SR	65.60	34.40
	K562	70.80	29.20
	CCRF-CEM	75.57	24.43
	MOLT-4	65.34	34.66
Colon	HCT-15	70.80	29.20
Renal	UO31	69.43	30.57
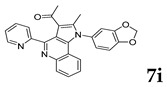			
**Cancer Panel**	**Cell line**	**G%**	**GI%**
Renal	UO31	72.34	27.66
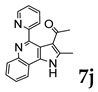			
**Cancer Panel**	**Cell line**	**G%**	**GI%**
Renal	UO31	68.98	31.02
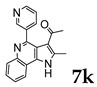			
**Cancer Panel**	**Cell line**	**G%**	**GI%**
Leukemia	SR	70.23	29.77
Renal	UO31	60.63	39.37
Breast	T-47D	7265	27.35
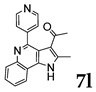			
**Cancer Panel**	**Cell line**	**G%**	**GI%**
Leukemia	SR	47.98	52.02
	K562	75.04	24.96
NSCLC	NCI-H522	73.75	26.25
Melanoma	UACC62	71.94	28.06
Renal	UO31	52.21	47.79

G%: Cell growth percent at 10 μM compared to untreated cells. GI%: Cell growth inhibition at 10 μM compared to untreated cells (As NCI protocols).

**Table 2:** Key calculated physicochemical and ADME parameters for compounds **7d** and **7f** (SwissADME).

Compound	MW	Consensus Log P	TPSA	GI Absorption	L, G, V, E, and M Violations *	PAINS
**7d**	344.36	3.77	64.21	High	0	0
**7f**	346.38	3.52	75.21	High	0	0

* L, G, V, E, and M violations stands for Lipinski, Ghose, Veber, Egan, or Muegge violations.

**Table 3:** Antiproliferative activity (MTS) GI_50_ (μM) ^§^ of derivatives **7g ^$^**–**i ^$^**, **m**–**p**.

Tumour Cell	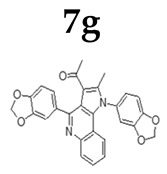	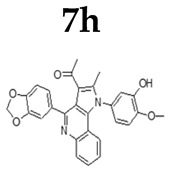	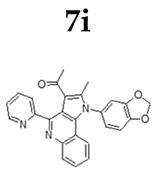	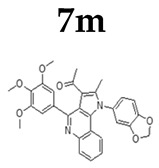	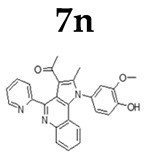	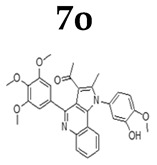	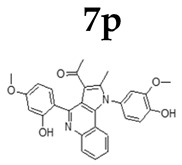
LAN5	17.8	16.6	77.4	25.2	90.8	27.2	33.6
H292	91.3	38.2	67.2	55	92.4	40.4	22.4
16HBE	22.2	17.9	52	16.5	59.4	22.3	34.2
HeLa	75.3	38.5	40.3	38.2	46.7	71.3	16.4
MCF7	10.6	15.7	16.7	18.4	17.5	13.6	2.8
Caco2	20.2	16.6	58.5	28.2	50.7	15.8	25.3

^§^ GI_50_ (μM) is the concentration expressed in micromolar to inhibit the proliferation of 50% of tumour cells with respect to the untreated cells taken as reference. Data are means +/− SEM (Standard Error of the Mean) of triplicate determination. **^$^** Refers to compounds also tested in the NCI 60 tumour cell line (SRD) and here tested against other tumour cell lines not included in the NCI tumour panels.

**Table 4:** Docking score and IFD score values (Kcal/mol) for compound **7m** against Tubulin and Topoisomerase.

Tubulin (1SA0)			Topoisomerase II (3QX3)		
Cpd *	Dock *	IFD *	Cpd *	Dock *	IFD *
**7m**	−11.813	−1753.29	**7m**	−8.028	−1686.15
**Lig co-cryst**	−11.152	−1749.12	**Etoposide (Lig co-cryst)**	−7.552	−1685.51
**Podophyllotoxin**	−11.331	−1749.08	**Podophyllotoxin**	−6.232	−1681.10

* Cpd: Compound; * Dock: Docking score; * IFD: Induced Fit Docking score.

**Table 5:** Docking score and IFD score values (Kcal/mol) for compounds **7d**-**q** against the analyzed targets.

**CDK6**			**HSP90**			**IGF1R**		
Cpd *	Dock *	IFD *	Cpd *	Dock *	IFD *	Cpd *	Dock *	IFD *
**7o**	−10.381	−568	**7i**	−13.002	−479.36	**7m**	−11.967	−658.69
**7l**	−10.814	−567.42	**7p**	−13.558	−478.93	**7n**	−10.186	−658.08
**7n**	−9.875	−567.04	**Ref lig**	−11.662	−477.17	**7o**	−10.562	−656.07
**7i**	−11.246	−567.02	**7j**	−10.554	−476.58	**7h**	−10.664	−655.34
**7h**	−11.04	−566.38	**7l**	−10.476	−475.48	**7p**	−9.72	−654.49
**Ref lig**	−11.934	−566.26	**7n**	−9.924	−475.17	**7j**	−10.456	−654.43
**7j**	−10.296	−565.65	**7h**	−10.793	−475.1	**7i**	−10.208	−653.94
**7p**	−9.579	−564.6	**7f**	−10.432	−474.97	**7k**	−10.536	−653.63
**7k**	−9.093	−564.1	**7d**	−10.25	−474.74	**7f**	−9.157	−653.45
**7g**	−10.38	−563.37	**7q**	−10.007	−474.54	**7g**	−9.461	−653.44
**7d**	−10.323	−563.11	**7k**	−10.11	−473.91	**7d**	−10.782	−653.33
**7e**	−9.747	−562.89	**7g**	−10.566	−473.07	**Ref lig**	−9.929	−652.72
**7q**	−8.264	−562.73	**7o**	−9.967	−472.94	**7q**	−9.291	−652.35
**7m**	−8.32	−562.73	**7e**	−10.216	−472.22	**7l**	−8.84	−652.08
**7f**	−9.132	−562.63	**7m**	−8.986	−471.29	**7e**	−11.528	−652.04
**HDAC8**			**HDAC2**			**PIK3CA**		
Cpd *	Dock *	IFD *	Cpd *	Dock *	IFD *	Cpd *	Dock *	IFD *
**7j**	−9.346	−746.07	**7o**	−6.601	−845.79	**7o**	−10.417	−2036.46
**7l**	−8.985	−745.9	**7n**	−5.51	−845.61	**7n**	−8.961	−2035.25
**7d**	−10.333	−745.79	**7k**	−6.769	−845.28	**Ref lig**	−9.937	−2035.15
**7q**	−9.117	−744.87	**7j**	−6.221	−845.21	**7l**	−8.669	−2034.97
**Ref lig**	−10.116	−744.81	**7f**	−6.91	−845.17	**7i**	−9.43	−2034.95
**7e**	−8.741	−744.25	**7h**	−6.815	−845.08	**7m**	−9.031	−2034.93
**7n**	−7.742	−744.13	**7p**	−6.678	−844.96	**7p**	−9.146	−2034.92
**7k**	−8.324	−743.82	**7l**	−5.936	−844.42	**7h**	−9.319	−2034.65
**7m**	−8.171	−743.58	**7m**	−6.219	−843.99	**7f**	−8.439	−2033.96
**7i**	−7.766	−743.48	**7i**	−5.227	−843.69	**7g**	−10.58	−2033.72
**7p**	−7.804	−742.76	**7q**	−6.077	−843.68	**7j**	−6.799	−2033.45
**7f**	−7.143	−742.18	**7g**	−6.094	−842.73	**7k**	−8.445	−2033.15
**7h**	−7.763	−741.92	**7e**	−6.117	−842.5	**7q**	−8.695	−2032.86
**7g**	−8.199	−741.89	**7d**	−5.434	−842.45	**7d**	−8.233	−2032.48
**7o**	−7.749	−741.83	**Ref lig**	−4.997	−841.81	**7e**	−10.059	−2031.87

* Cpd: Compound; * Dock: Docking score; * IFD: Induced Fit Docking score.

## Data Availability

The original contributions presented in this study are included in the article/Supplementary Material. Further inquiries can be directed to the corresponding author(s).

## Supplementary Materials

The following supporting information can be downloaded at https://www.mdpi.com/article/10.3390/biom15121718/s1. Figure S1. One dose mean graph for compound **7d**. Figure S2. One dose mean graph for compound **7e**. Figure S3. One dose mean graph for compound **7f**. Figure S4. One dose mean graph for compound **7g**. Figure S5. One dose mean graph for compound **7h**. Figure S6. One dose mean graph for compound **7i**. Figure S7. One dose mean graph for compound **7j**. Figure S8. One dose mean graph for compound **7k**. Figure S9. One dose mean graph for compound **7l**. Figure S10. Doxorubicin (NCI testing) one dose at 10 μM (source NCI database). Figure S11. Podophyllotoxin (NCI testing) one dose at 10 μM (source NCI database). Figure S12. ^1^HNMR spectra for compound **7p**. Figure S13. Focused ^1^HNMR spectra for compound **7p**. Figure S14. ^13^CNMR spectra for compound **7p**. Figure S15. ^1^HNMR spectra for compound **7o**. Figure S16. Focused ^1^HNMR spectra for compound **7o**. Figure S17. ^13^CNMR spectra for compound **7o**. Figure S18. ^1^HNMR spectra for compound **7n**. Figure S19. Focused ^1^HNMR spectra for compound **7n**. Figure S20. ^13^CNMR spectra for compound **7n**. Figure S21. ^1^HNMR spectra for compound **7m**. Figure S22. Focused ^1^HNMR spectra for compound **7m**. Figure S23. ^13^CNMR spectra for compound **7m**. Figure S24. Focus on the most important spectra peaks for compound **7m** (153.20-153.09, 129.88-129.85, 60.29, 55.80). Figure S25. ^13^CNMR DEPT spectra for compound **7m**. Figure S26. Focus on the most important spectra peaks for compound **7m** (130.32, 127.42-126.14, 122.76, 120.69, 109.80-109.70, 106.22, 60.29, 55.80). Figure S27. ^1^HNMR spectra for compound **7l**. Figure S28. Focused ^1^HNMR spectra for compound **7l**. Figure S29. ^13^CNMR spectra for compound **7l**. Figure S30. ^1^HNMR spectra for compound **7k**. Figure S31. Focused ^1^HNMR spectra for compound **7k**. Figure S32. ^13^CNMR spectra for compound **7k**. Figure S33. ^1^HNMR spectra for compound **7j**. Figure S34. Focused ^1^HNMR spectra for compound **7j**. Figure S35. ^13^CNMR spectra for compound **7j**. Figure S36. ^1^HNMR spectra for compound **7i**. Figure S37. Focused ^1^HNMR spectra for compound **7i**. Figure S38. ^13^CNMR spectra for compound **7i**. Figure S39. ^1^HNMR spectra for compound **7h**. Figure S40. Focused ^1^HNMR spectra for compound **7h**. Figure S41. ^13^CNMR spectra for compound **7h**. Figure S42. ^1^HNMR spectra for compound **7g**. Figure S43. Focused ^1^HNMR spectra for compound **7g**. Figure S44. ^13^CNMR spectra for compound **7g**. Figure S45. ^1^HNMR spectra for compound **7f**. Figure S46. Focused ^1^HNMR spectra for compound **7f**. Figure S47. ^13^CNMR spectra for compound **7f**. Figure S48. ^1^HNMR spectra for compound **7e**. Figure S49. Focused ^1^HNMR spectra for compound **7e**. Figure S50. ^13^CNMR spectra for compound **7e**. Figure S51. ^1^HNMR spectra for compound **3c**. Figure S52. Focused ^1^HNMR spectra for compound **3c**. Figure S53. ^13^CNMR spectra for compound **3c**. Figure S54. ^1^HNMR spectra for compound **3b**. Figure S55. Focused ^1^HNMR spectra for compound **3b**. Figure S56. ^13^CNMR spectra for compound **3b**. Figure S57. ^1^HNMR spectra for compound **2c**. Figure S58. Focused ^1^HNMR spectra for compound **2c**. Figure S59. ^13^CNMR spectra for compound **2c**. Figure S60. ^1^HNMR spectra for compound **2b**. Figure S61. Focused ^1^HNMR spectra for compound **2b**. Figure S62. ^13^CNMR spectra for compound **2b**. Figure S63. ^1^HNMR spectra for compound **8r**. Figure S64. Focused ^1^HNMR spectra for compound **8e**. Figure S65. ^13^CNMR spectra for compound **8r**. Figure S66. 3D binding poses of compounds **7d**, **7h**, and **7i** within the **CDK6** active site. Table S1. Complete SwissADME output for compounds **7d** and **7f**, including calculated physicochemical properties, lipophilicity descriptors, solubility predictions, drug-likeness filters, medicinal chemistry alerts, and ADME-related predictions (GI absorption, BBB permeability, P-gp substrate status, and CYP inhibition profiles).

## Abbreviations

The following abbreviations are used in this manuscript:

CNS	Central Nervous System
GI_50_	Growth Inhibition 50%
G%	percentage of cell growth
HSP90	Heat Shock Protein 90
GI%	percentage of cell growth inhibition
IGF1R	Insulin-like Growth Factor 1 Receptor
IFD	Induced Fit Docking
NCI	National Cancer Institute
PQ	Pyrrolo[3,2-c]quinoline
PPT	Podophyllotoxin
SRB	Sulforhodamine B
TSA	Toluenesulfonic acid
STS	Staurosporine
MTS	Tetrazolium salt (colorimetric agent for biotest)
CDK6	Cyclin-dependent Kinase 6
HDAC2	Histone Deacetylase 2
HDAC8	Histone Deacetylase 8
PIK3CA	PhosphatidylInositol-4,5-bisphosphate 3-Kinase Catalytic subunit alpha
