# Supplementary material for: Tuning Scaffold Properties of New 1,4-Substituted Pyrrolo[3,2-c]quinoline Derivatives Endowed with Anticancer Potential, New Biological and In Silico Insights
Source: Biomolecules. 2025 Dec 10;15(12):1718. doi: 10.3390/biom15121718 (PMC12730935; doi:10.3390/biom15121718)
Supplement: Supplementary file 1 [file biomolecules-15-01718-s001.zip › biomolecules-3990396-supplementary.pdf]

Supplementary Material

Tuning scaffold properties of new 1,4-substituted pyrrolo[3,2-c]quinoline derivatives endowed with anticancer potential, new biological and *in silico* insights

Francesco Mingoia[1]*, Caterina Disano[2], Claudia D'Anna[2], Marco Fazzari[3], Alessia Bono[4,5], Gabriele La Monica[4], Annamaria Martorana[4], Antonino Lauria[4,6]*

[1]Istituto per lo Studio dei Materiali Nanostrutturati (ISMN), Consiglio Nazionale delle Ricerche (CNR), Via U. La Malfa 153, Palermo, 90146, Italy;
[2]Istituto di Farmacologia Traslazionale (IFT), Consiglio Nazionale delle Ricerche (CNR), Via U. La Malfa 153, Palermo, 90146, Italy;
[3]Department of Pharmacology and Chemical Biology, University of Pittsburgh, Pittsburgh, PA, 15261, USA;
[4]Dipartimento di Scienze e Tecnologie Biologiche Chimiche e Farmaceutiche "STEBICEF" University of Palermo, Viale delle Scienze, Ed. 17, Palermo, 90128, Italy;
[5]Fondazione Umberto Veronesi (FUV), via Solferino 19, 20121 Milano, Italy;
[6]National Biodiversity Future Center (NBFC), Piazza Marina 61, 90133 Palermo, Italy.

*Corresponding authors: Francesco Mingoia, francesco.mingoia@ismn.cnr.it; Antonino Lauria, antonino.lauria@unipa.it

As follow, we list the complete antiproliferative NCI screening relatively to all the panel/cell line tested (one dose mean graphs performed at 10µM.)

"The PI and all the authors wish to thank the National Cancer Institute Developmental Therapeutics Program (NCI/DTP) https://dtp.cancer.gov for providing screening data of compounds present in this manuscript. Specifically, NSC # number: NSC752654\1 **7d**; NSC798079\1 **7e**; NSC798081\1 **7f**; NSC798091\1 **7g**; NSC798090\1 **7h**; NSC798092\1 **7i**; NSC798084\1 **7j;** NSC798085\1 **7k**; NSC798086\1 7l. Below are the "one dose mean graph" performed by National Cancer Institute (NCI), USA (MD).

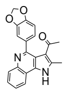

NSC752654\1 **7d (ex4g,** Eur. J. Med. Chem., 2023**)**

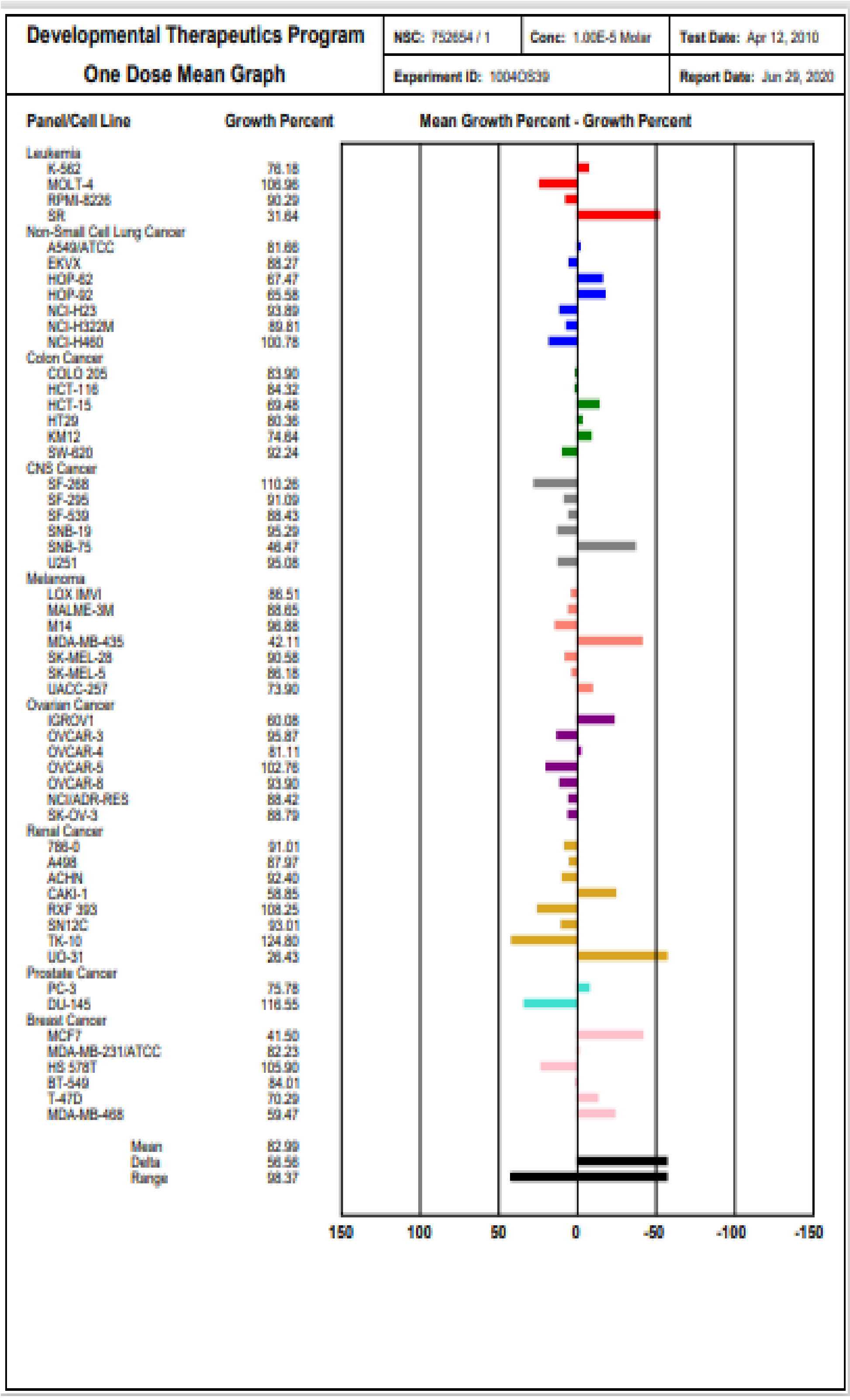

Figure S1. One dose mean graph for compound **7d**.

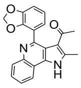 NSC798079\1 **7e**

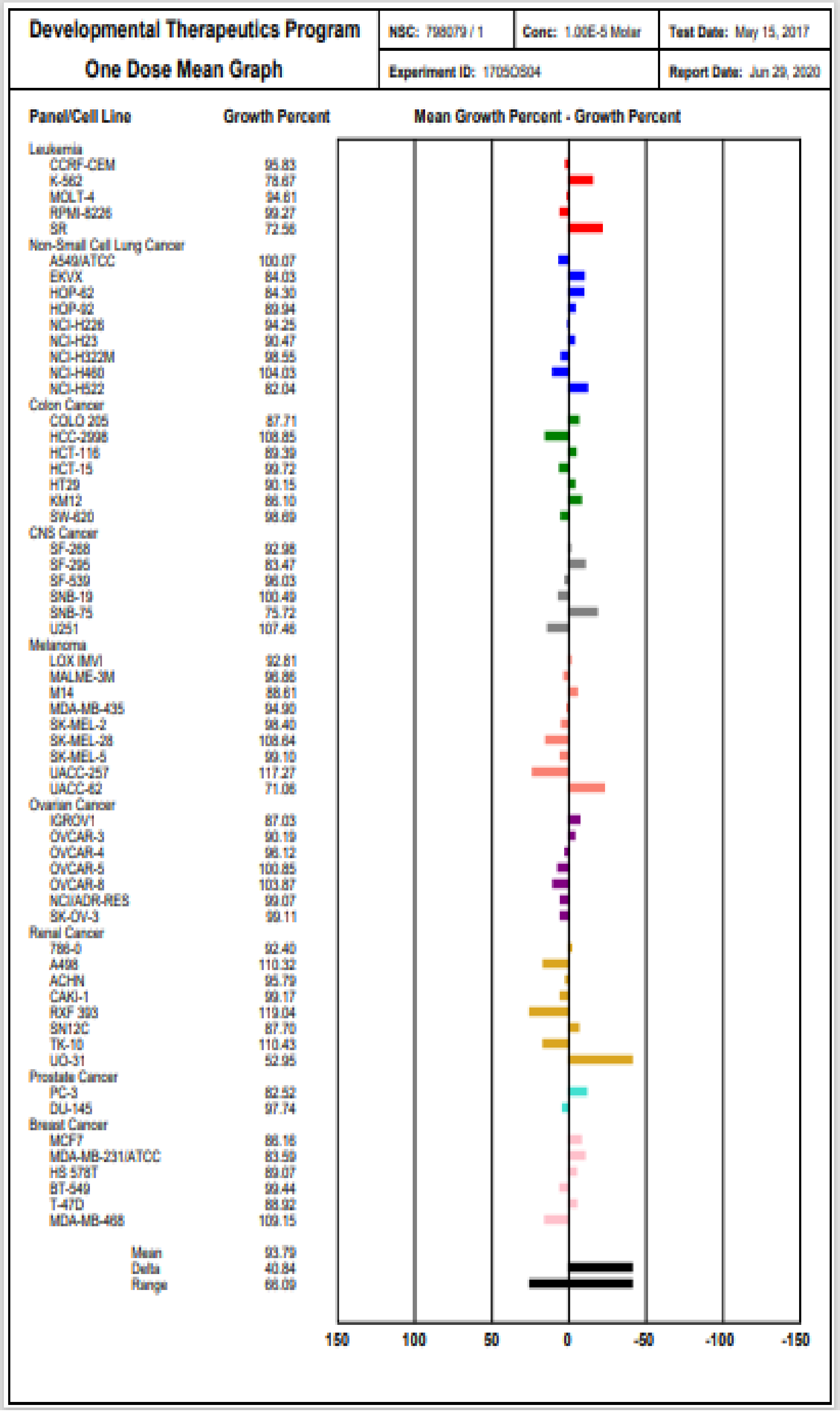

Figure S2. One dose mean graph for compound **7e**.

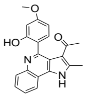

NSC798081\1 **7f**

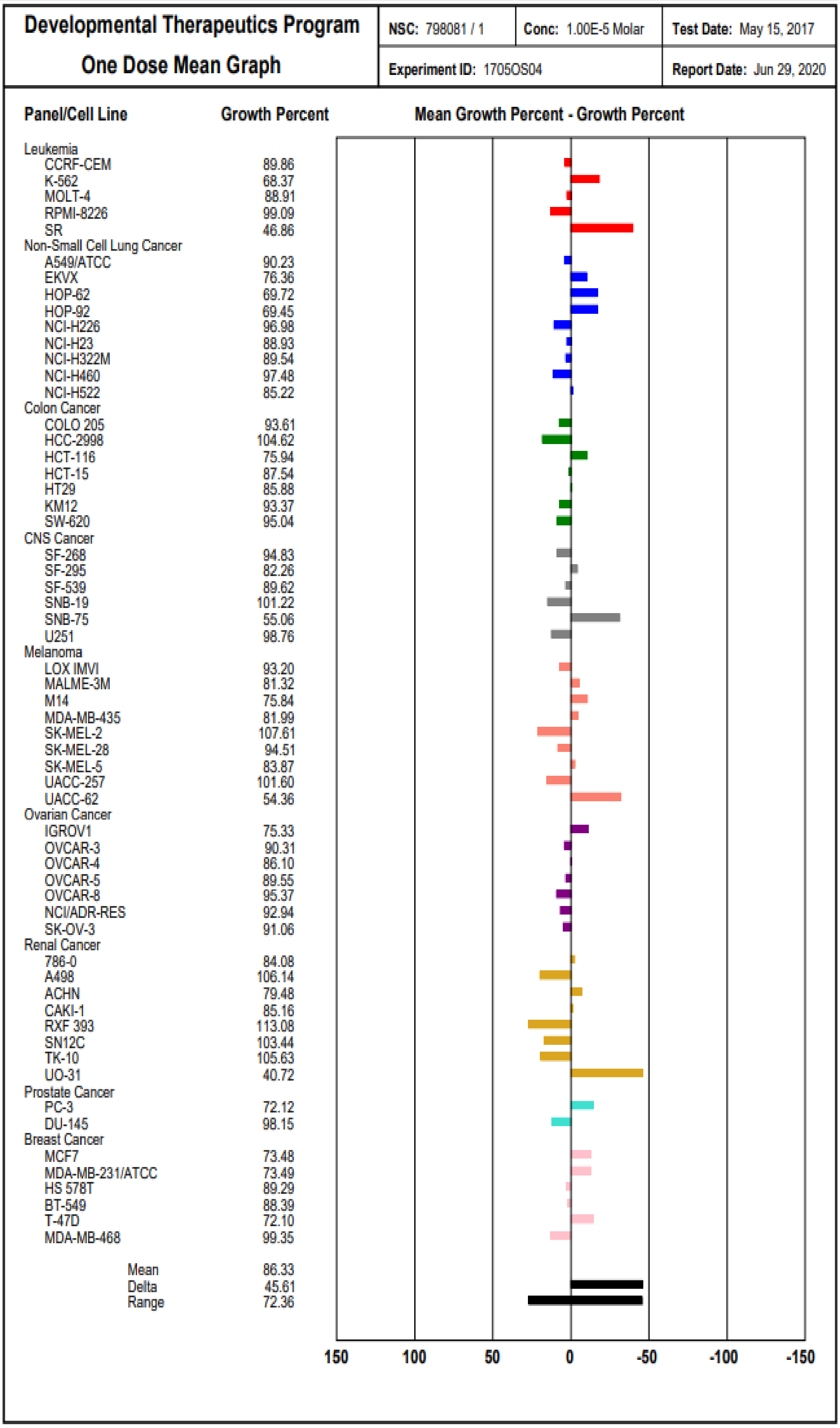

Figure S3. One dose mean graph for compound **7f**.

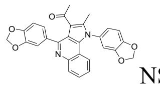

NSC798091\1 **7g**

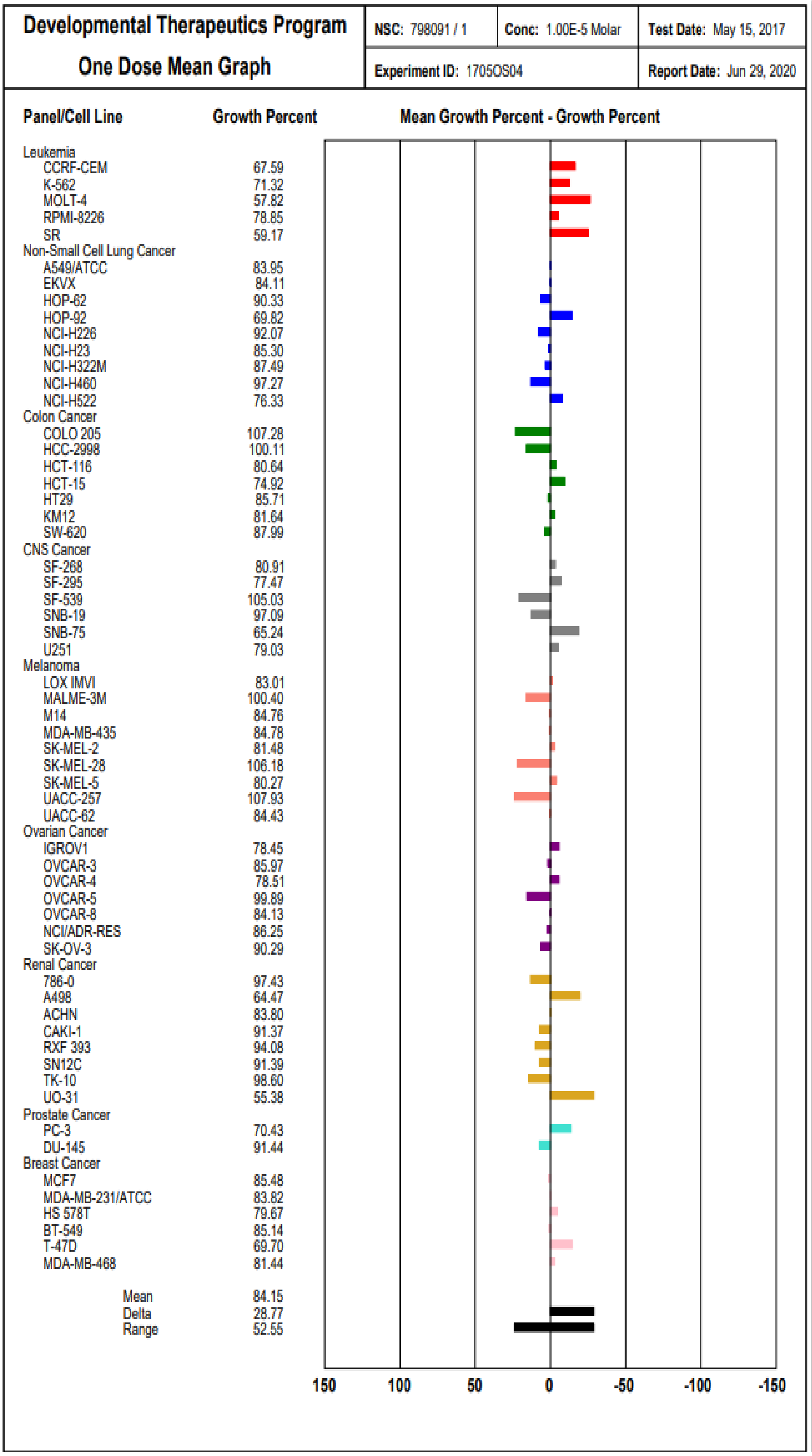

Developmental Therapeutics Program

One Dose Mean Graph

**NSC:** 798091 / 1	**Conc:** 1.00E-5 Molar	**Test Date:** May 15, 2017
**Experiment ID:** 1705OS04		**Report Date:** Jun 29, 2020

Panel/Cell Line	Growth Percent
**Leukemia**	
CCRF-CEM	67.59
K-562	71.32
MOLT-4	57.82
RPMI-8226	78.85
SR	59.17
**Non-Small Cell Lung Cancer**	
A549/ATCC	83.95
EKVX	84.11
HOP-62	90.33
HOP-92	69.82
NCI-H226	92.07
NCI-H23	85.30
NCI-H322M	87.49
NCI-H460	97.27
NCI-H522	76.33
**Colon Cancer**	
COLO 205	107.28
HCC-2998	100.11
HCT-116	80.64
HCT-15	74.92
HT29	85.71
KM12	81.64
SW-620	87.99
**CNS Cancer**	
SF-268	80.91
SF-295	77.47
SF-539	105.03
SNB-19	97.09
SNB-75	65.24
U251	79.03
**Melanoma**	
LOX IMVI	83.01
MALME-3M	100.40
M14	84.76
MDA-MB-435	84.78
SK-MEL-2	81.48
SK-MEL-28	106.18
SK-MEL-5	80.27
UACC-257	107.93
UACC-62	84.43
**Ovarian Cancer**	
IGROV1	78.45
OVCAR-3	85.97
OVCAR-4	78.51
OVCAR-5	99.89
OVCAR-8	84.13
NCI/ADR-RES	86.25
SK-OV-3	90.29
**Renal Cancer**	
786-0	97.43
A498	64.47
ACHN	83.80
CAKI-1	91.37
RXF 393	94.08
SN12C	91.39
TK-10	98.60
UO-31	55.38
**Prostate Cancer**	
PC-3	70.43
DU-145	91.44
**Breast Cancer**	
MCF7	85.48
MDA-MB-231/ATCC	83.82
HS 578T	79.67
BT-549	85.14
T-47D	69.70
MDA-MB-468	81.44
Mean	84.15
Delta	28.77
Range	52.55

Figure S4. One dose mean graph for compound **7g**.

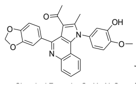

NSC798090\1 **7h**

Developmental Therapeutics Program | **NSC:** 798090 / 1 | **Conc:** 1.00E-5 Molar | **Test Date:** May 15, 2017

One Dose Mean Graph | **Experiment ID:** 1705OS04 | **Report Date:** Jun 29, 2020

Panel/Cell Line	Growth Percent
Leukemia	
CCRF-CEM	75.57
K-562	70.80
MOLT-4	65.34
RPMI-8226	78.17
SR	65.60
Non-Small Cell Lung Cancer	
A549/ATCC	92.31
EKVX	86.13
HOP-62	103.86
HOP-92	87.39
NCI-H226	102.94
NCI-H23	102.85
NCI-H322M	88.58
NCI-H460	94.01
NCI-H522	95.73
Colon Cancer	
COLO 205	109.30
HCC-2998	109.22
HCT-116	81.23
HCT-15	70.80
HT29	97.89
KM12	87.58
SW-620	89.20
CNS Cancer	
SF-268	82.56
SF-295	82.23
SF-539	97.05
SNB-19	87.77
SNB-75	82.81
U251	88.90
Melanoma	
LOX IMVI	91.76
MALME-3M	96.69
M14	88.69
MDA-MB-435	90.98
SK-MEL-2	86.23
SK-MEL-28	96.19
SK-MEL-5	84.71
UACC-257	109.03
UACC-62	91.11
Ovarian Cancer	
IGROV1	94.17
OVCAR-3	84.24
OVCAR-4	95.63
OVCAR-5	103.08
OVCAR-8	86.22
NCI/ADR-RES	92.66
SK-OV-3	107.62
Renal Cancer	
786-0	88.08
A498	82.36
ACHN	86.90
CAKI-1	97.27
RXF 393	105.74
SN12C	88.85
TK-10	108.59
UO-31	69.43
Prostate Cancer	
PC-3	75.05
DU-145	91.59
Breast Cancer	
MCF7	91.25
MDA-MB-231/ATCC	86.15
HS 578T	77.24
BT-549	95.50
T-47D	81.76
MDA-MB-468	98.05
Mean	89.81
Delta	24.47
Range	43.96

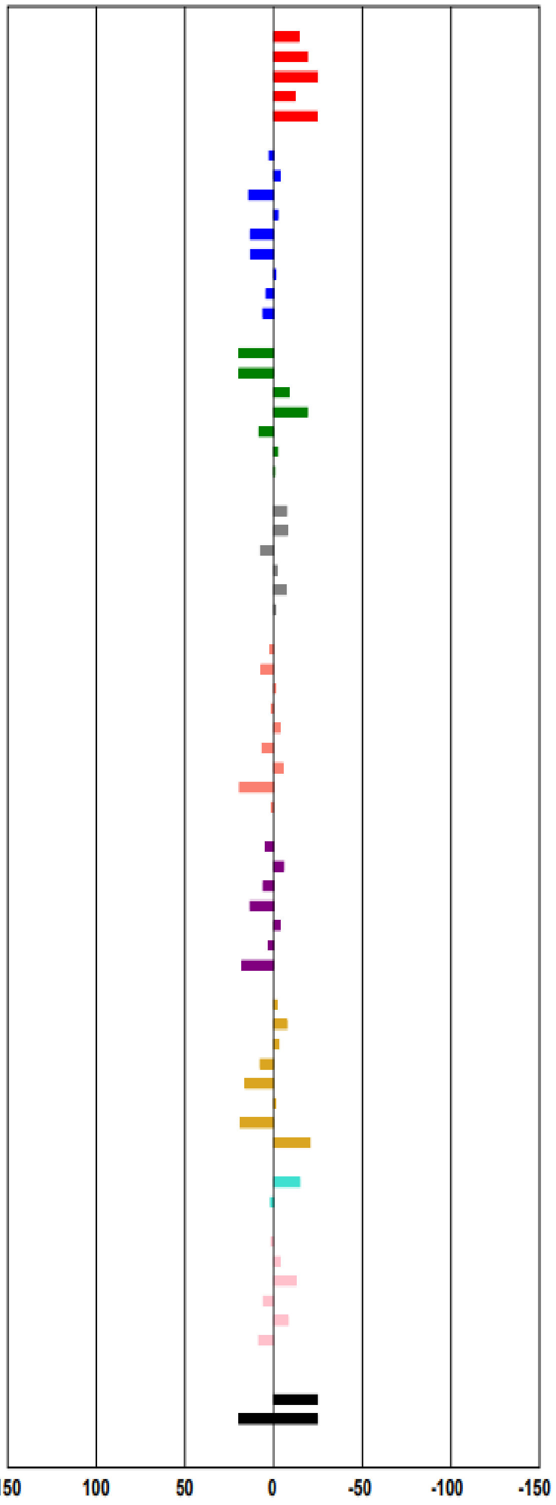

Figure S5. One dose mean graph for compound **7h**.

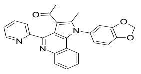

NSC798092\1 **7i**

Developmental Therapeutics Program One Dose Mean Graph	NSC: 798092 / 1	Conc: 1.00E-5 Molar	Test Date: May 15, 2017
	Experiment ID: 1705OS04		Report Date: Jun 29, 2020

Panel/Cell Line	Growth Percent
**Leukemia**	
CCRF-CEM	82.39
K-562	89.38
MOLT-4	83.97
RPMI-8226	90.42
SR	81.26
**Non-Small Cell Lung Cancer**	
A549/ATCC	95.78
EKVX	91.22
HOP-62	99.48
HOP-92	79.34
NCI-H226	97.74
NCI-H23	93.05
NCI-H322M	88.38
NCI-H460	99.24
NCI-H522	85.11
**Colon Cancer**	
COLO 205	109.80
HCC-2998	102.91
HCT-116	80.49
HCT-15	76.20
HT29	88.95
KM12	96.94
SW-620	96.02
**CNS Cancer**	
SF-268	99.14
SF-295	88.94
SF-539	101.57
SNB-19	94.18
SNB-75	82.62
U251	100.12
**Melanoma**	
LOX IMVI	94.33
MALME-3M	101.34
M14	96.94
MDA-MB-435	86.20
SK-MEL-2	97.97
SK-MEL-28	101.80
SK-MEL-5	89.28
UACC-257	106.97
UACC-62	86.87
**Ovarian Cancer**	
IGROV1	79.11
OVCAR-3	90.60
OVCAR-4	87.51
OVCAR-5	99.65
OVCAR-8	98.63
NCI/ADR-RES	98.17
SK-OV-3	101.45
**Renal Cancer**	
786-0	97.48
A498	99.33
ACHN	92.91
CAKI-1	98.82
RXF 393	98.98
SN12C	99.12
TK-10	102.15
UO-31	72.34
**Prostate Cancer**	
PC-3	87.60
DU-145	89.13
**Breast Cancer**	
MCF7	88.86
MDA-MB-231/ATCC	85.25
HS 578T	91.35
BT-549	100.70
T-47D	81.63
MDA-MB-468	86.53
Mean	92.60
Delta	20.26
Range	37.46

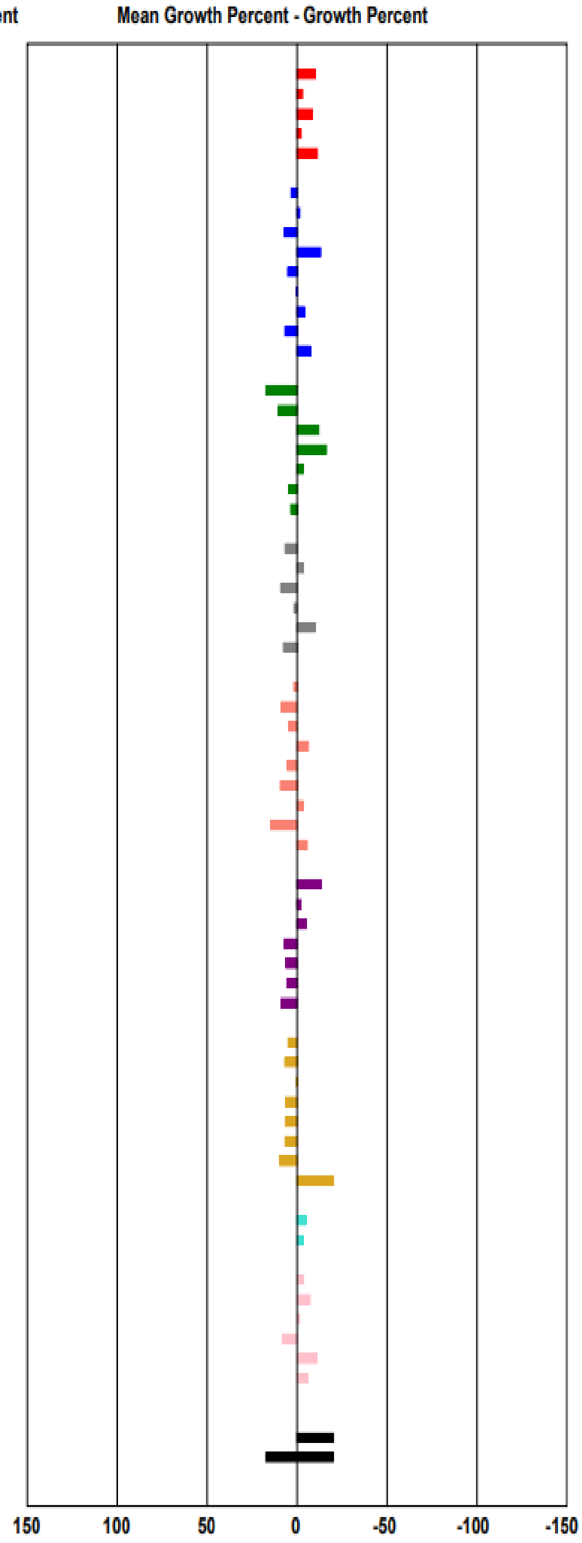

Figure S6. One dose mean graph for compound **7i**.

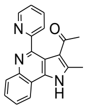

NSC798084\1 **7j**

Developmental Therapeutics Program One Dose Mean Graph	NSC: 798084 / 1	Conc: 1.00E-5 Molar	Test Date: May 15, 2017
	Experiment ID: 1705OS04		Report Date: Jun 29, 2020

Panel/Cell Line	Growth Percent
**Leukemia**	
CCRF-CEM	90.92
K-562	90.47
MOLT-4	108.48
RPMI-8226	107.25
SR	82.06
**Non-Small Cell Lung Cancer**	
A549/ATCC	97.95
EKVX	91.75
HOP-62	95.89
HOP-92	108.21
NCI-H226	99.70
NCI-H23	96.21
NCI-H322M	94.55
NCI-H460	109.89
NCI-H522	80.31
**Colon Cancer**	
COLO 205	113.17
HCC-2998	113.80
HCT-116	89.39
HCT-15	96.93
HT29	93.11
KM12	103.32
SW-620	103.51
**CNS Cancer**	
SF-268	99.40
SF-295	88.57
SF-539	94.63
SNB-19	102.09
SNB-75	81.91
U251	106.38
**Melanoma**	
LOX IMVI	101.61
MALME-3M	97.86
M14	93.53
MDA-MB-435	94.46
SK-MEL-2	104.34
SK-MEL-28	99.33
SK-MEL-5	103.20
UACC-257	101.86
UACC-62	87.63
**Ovarian Cancer**	
IGROV1	101.84
OVCAR-3	93.03
OVCAR-4	90.00
OVCAR-5	94.69
OVCAR-8	109.68
NCI/ADR-RES	102.00
SK-OV-3	106.45
**Renal Cancer**	
786-0	98.18
A498	109.01
ACHN	96.37
CAKI-1	94.59
RXF 393	121.07
SN12C	99.72
TK-10	99.38
UO-31	68.98
**Prostate Cancer**	
PC-3	91.78
DU-145	95.94
**Breast Cancer**	
MCF7	82.76
MDA-MB-231/ATCC	94.45
HS 578T	96.42
BT-549	100.74
T-47D	85.81
MDA-MB-468	103.93
Mean	97.64
Delta	28.66
Range	52.09

Mean Growth Percent - Growth Percent

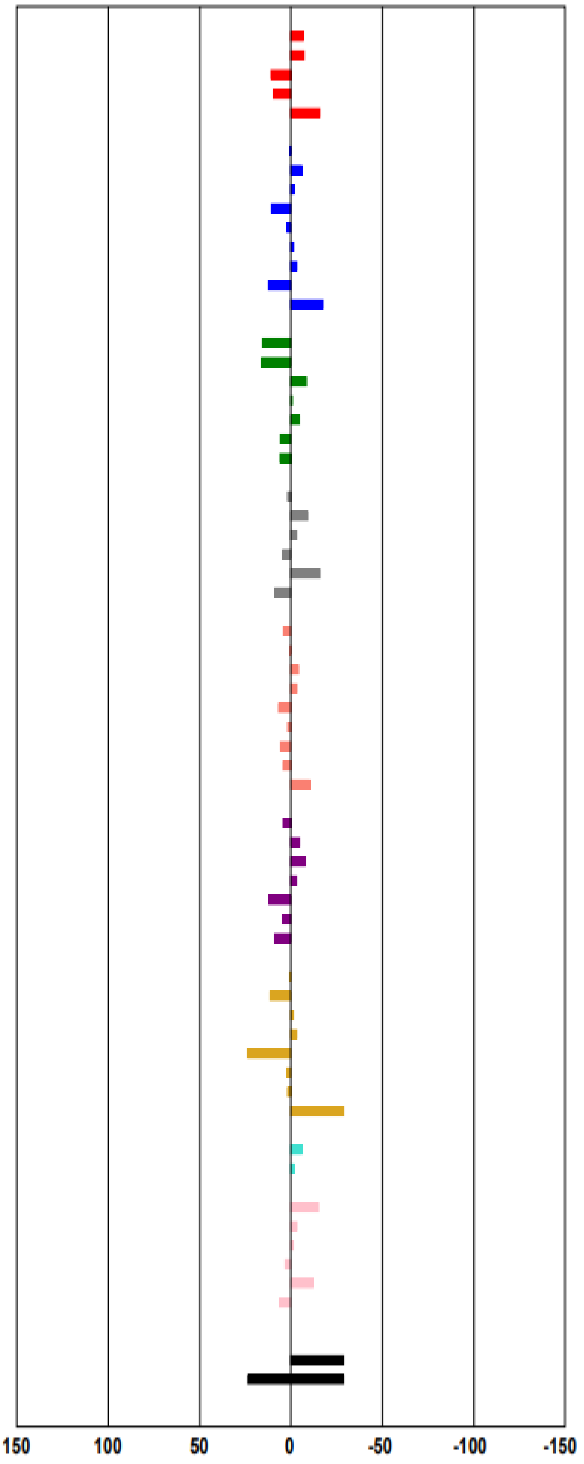

Figure S7. One dose mean graph for compound **7j**.

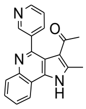

NSC798085\1 **7k**

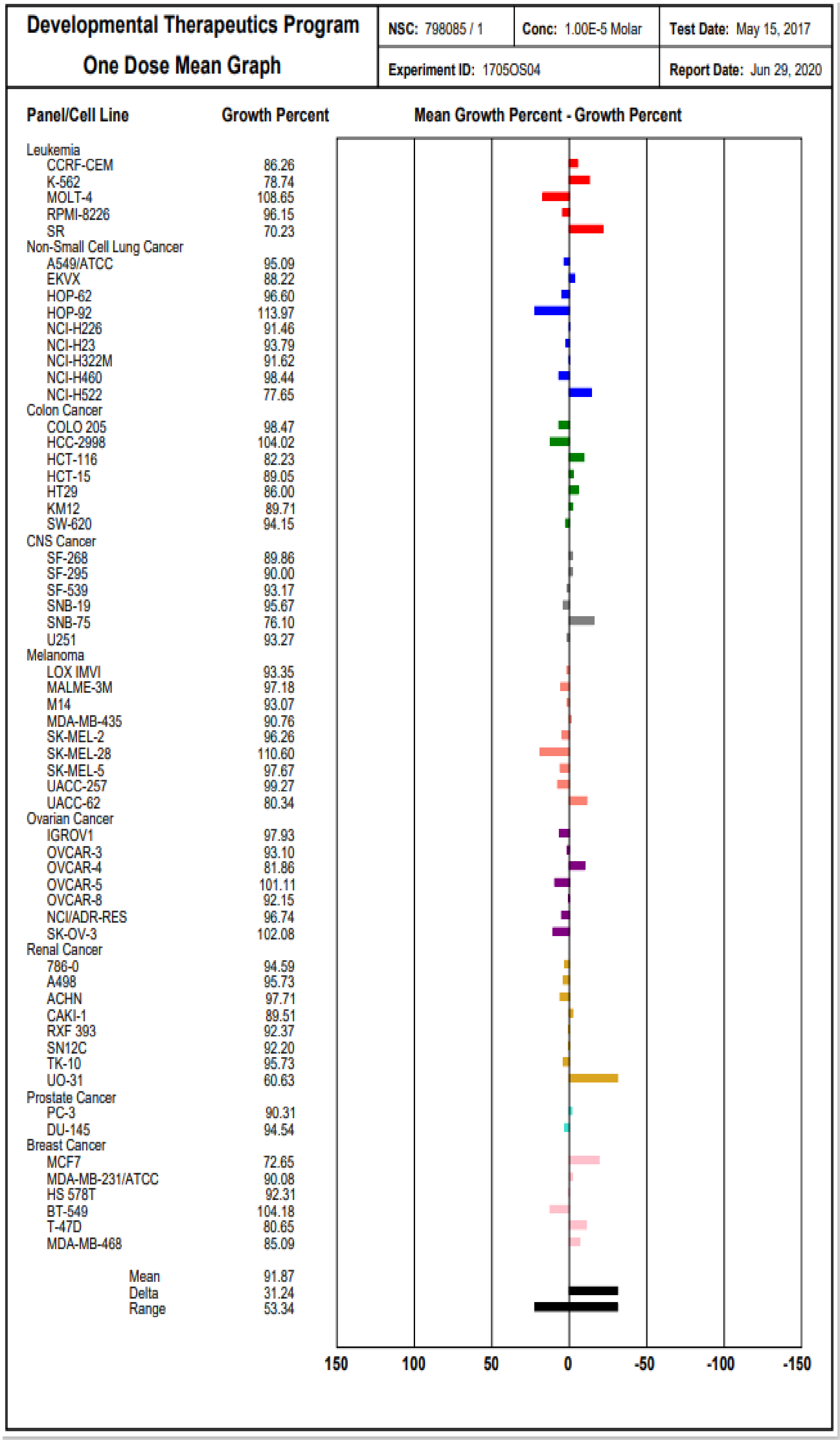

Figure S8. One dose mean graph for compound **7k**.

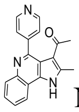

NSC798086\1 **7l**

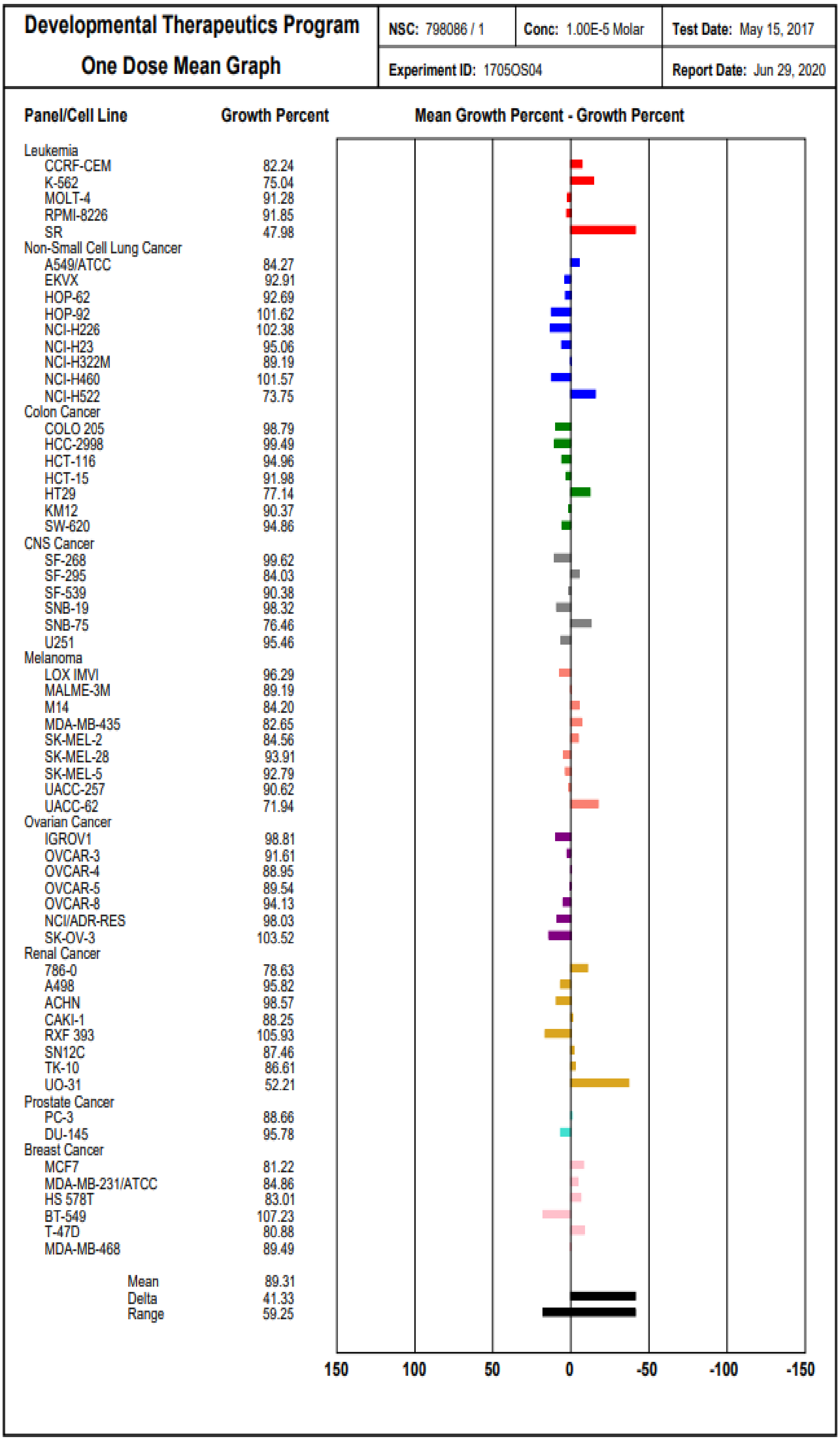

Figure S9. One dose mean graph for compound **7l**.

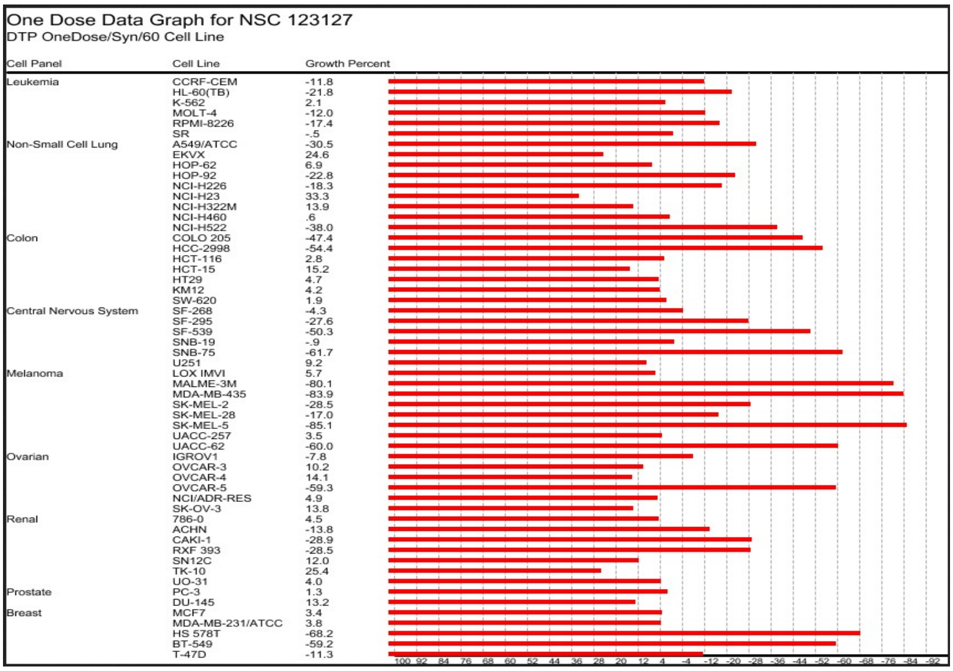

Figure S10. Doxorubicin (NCI testing) one dose at 10 μM (source NCI database).

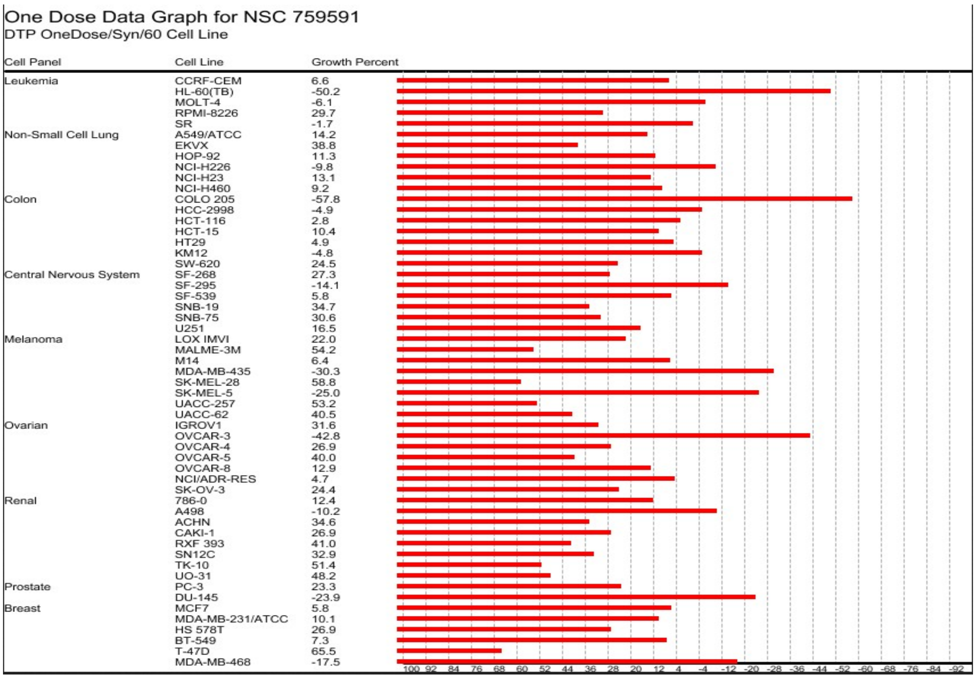

Figure S11. Podophyllotoxin (NCI testing) one dose at 10 μM (source NCI database).

Interpretation of One-Dose Data: The One-Concentration Screen data will be reported as a mean graph of the percent growth of treated cells and will be similar in appearance to mean graphs from the Five-Concentration Screen. The number reported for the One-Concentration Screen is growth relative to the vehicle control, and relative to the number of cells at time zero. This allows detection of both growth inhibition (values between 0 and 100) and lethality (values less than 0). This is the same as for the Five-Concentration Screen, described below. For example, a value of 100 means no growth inhibition. A value of 40 would mean 60% growth inhibition. A value of 0 means no net growth over the course of the experiment. A value of -40 would mean 40% lethality. A value of -100 means all the cells are dead.

Below are the ^{1}H NMR, ^{13}C NMR spectra of test new compounds **7e-p**, **3 b, c, 2 b, c**.

7p

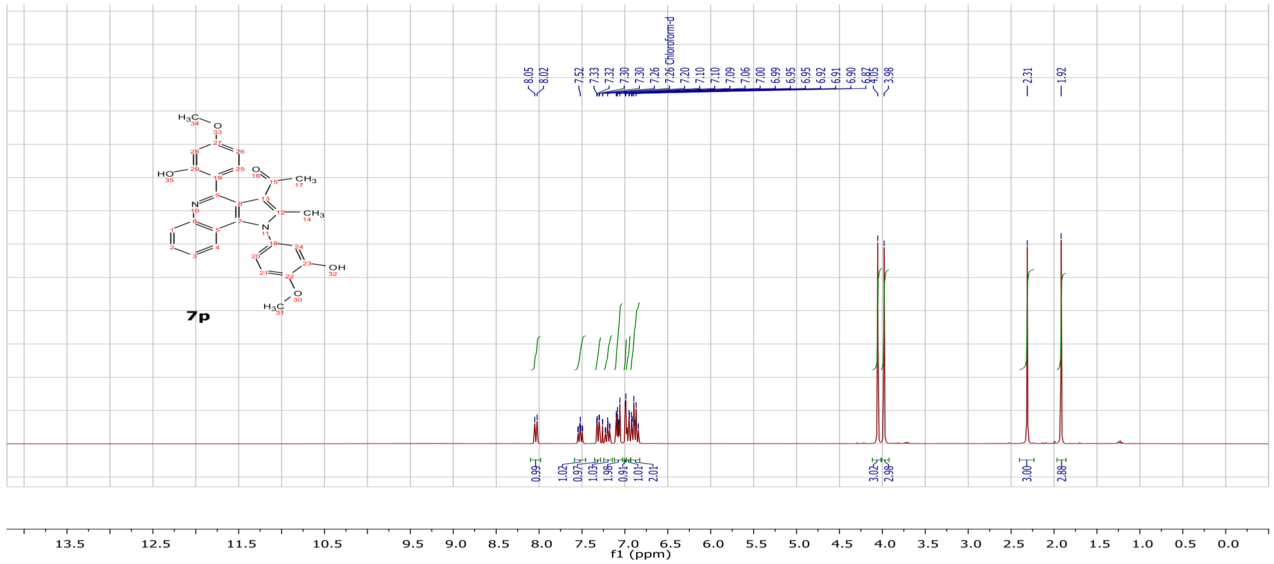

Figure S12. ^{1}HNMR spectra for compound **7p**.

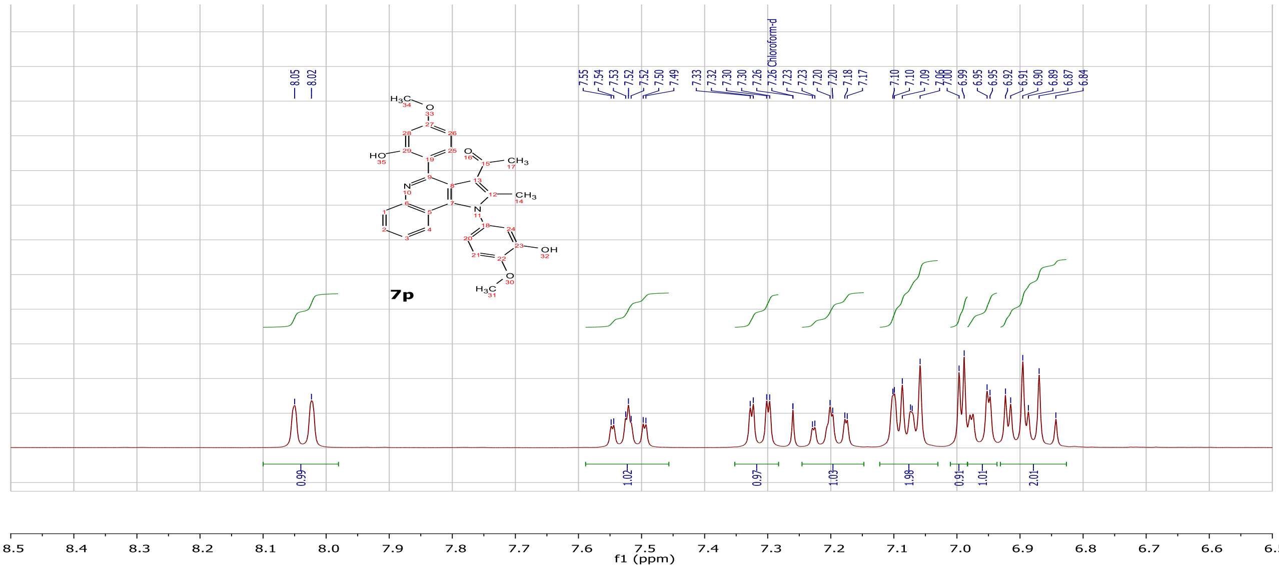

Figure S13. Focused ^{1}HNMR spectra for compound **7p**.

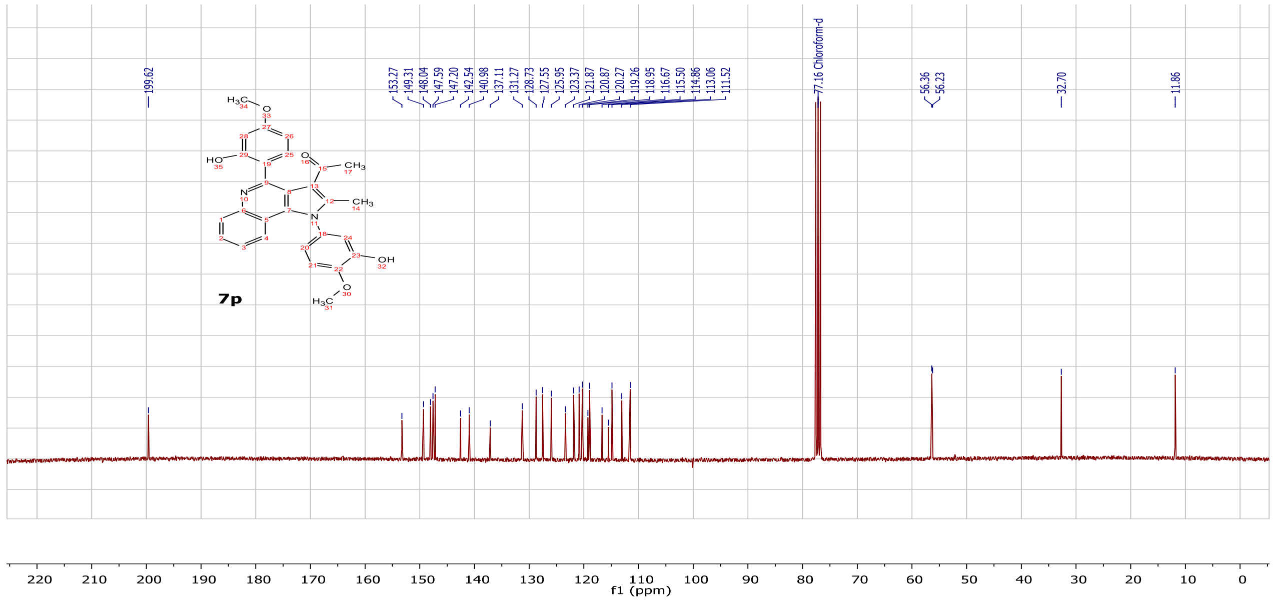

Figure S14. ^{13}CNMR spectra for compound **7p**.

7o

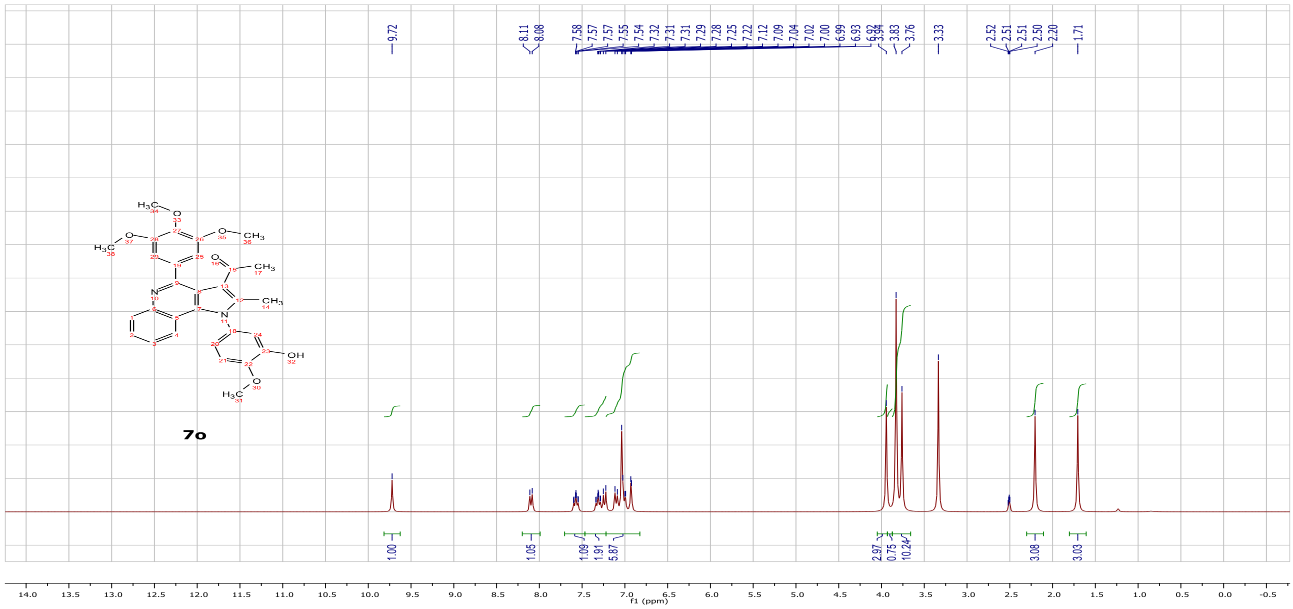

Figure S15. ^{1}HNMR spectra for compound **7o**.

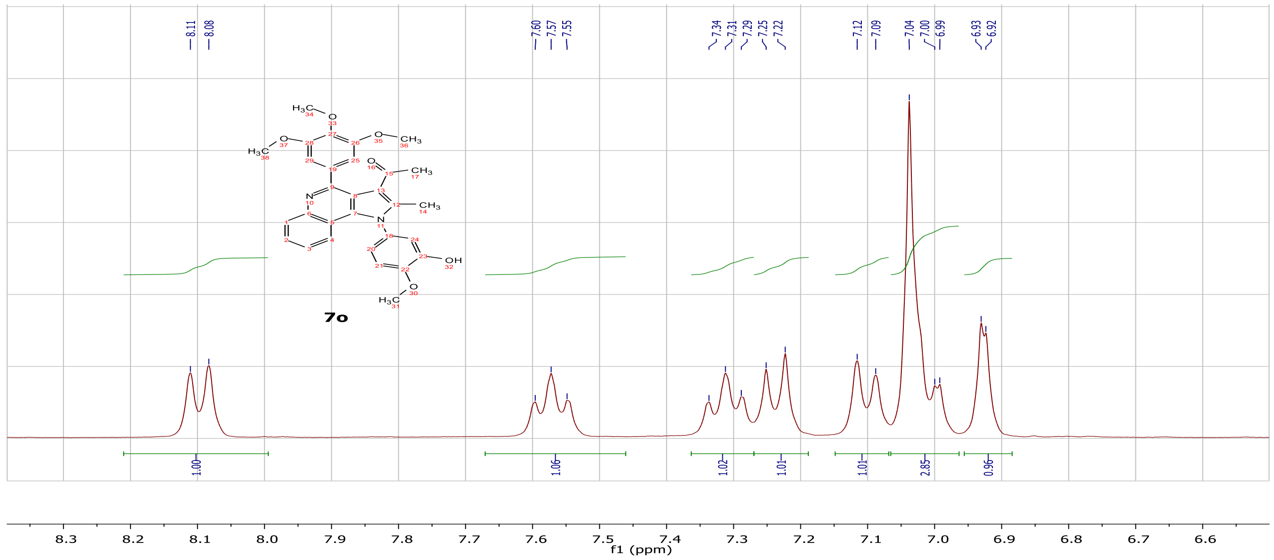

Figure S16. Focused ^{1}HNMR spectra for compound **7o**.

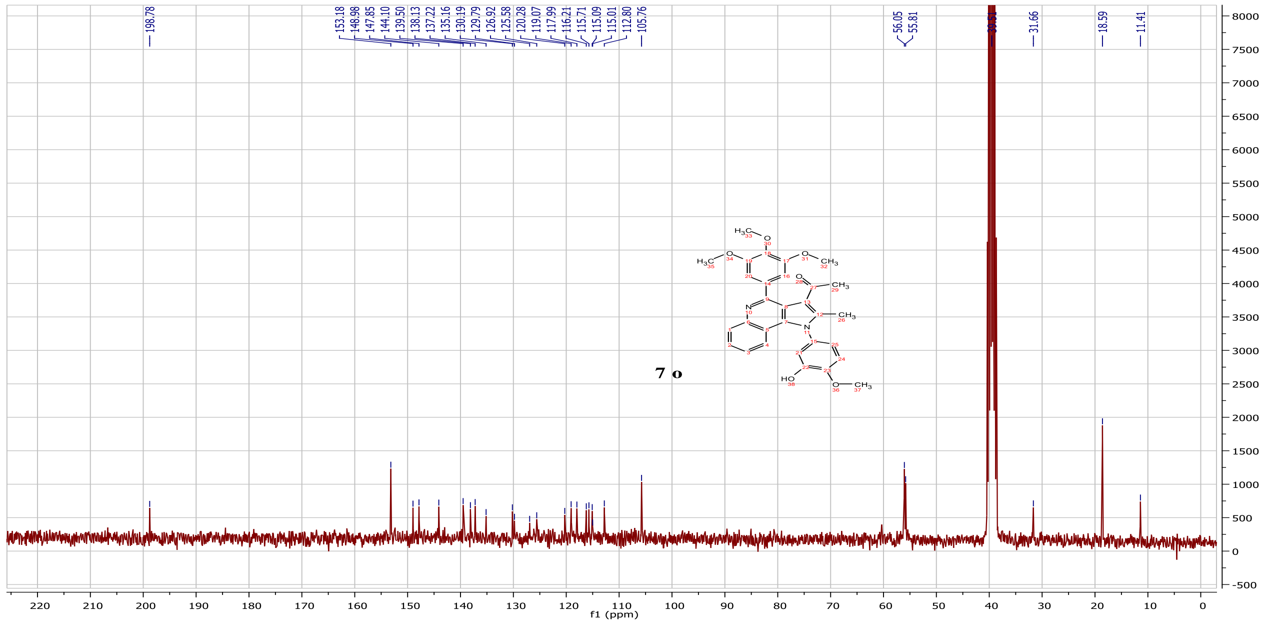

Figure S17. ^{13}CNMR spectra for compound **7o**.

7n

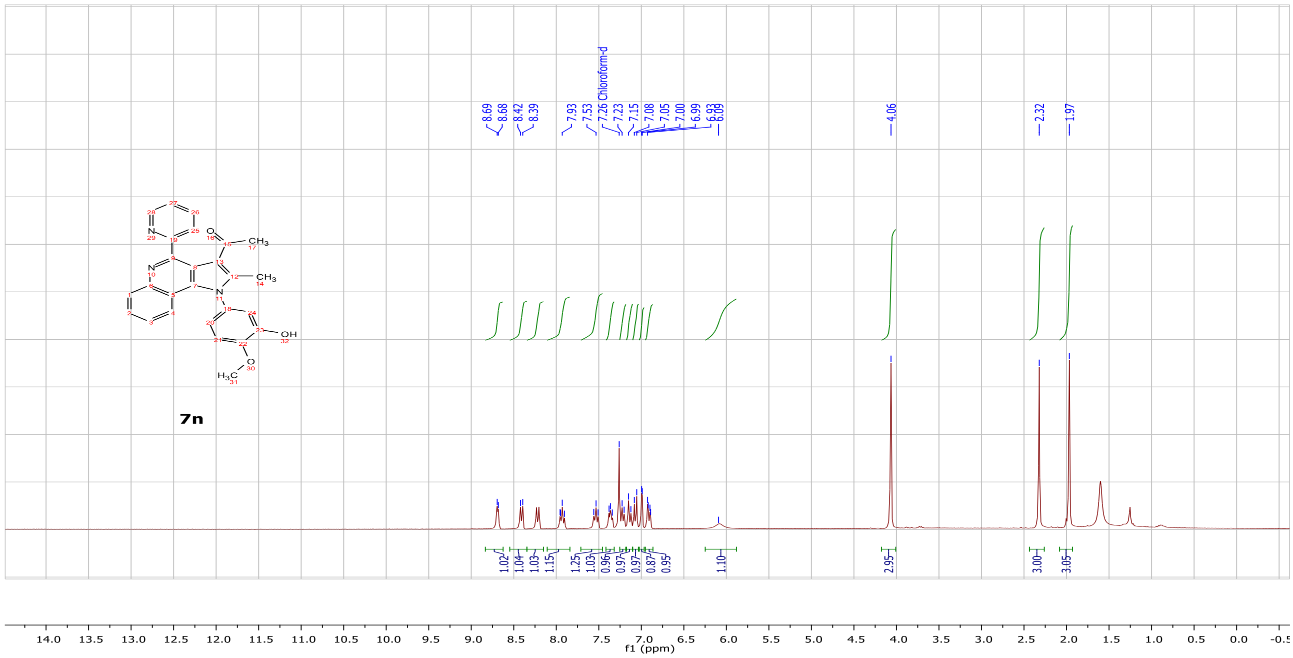

Figure S18. ^{1}HNMR spectra for compound **7n**.

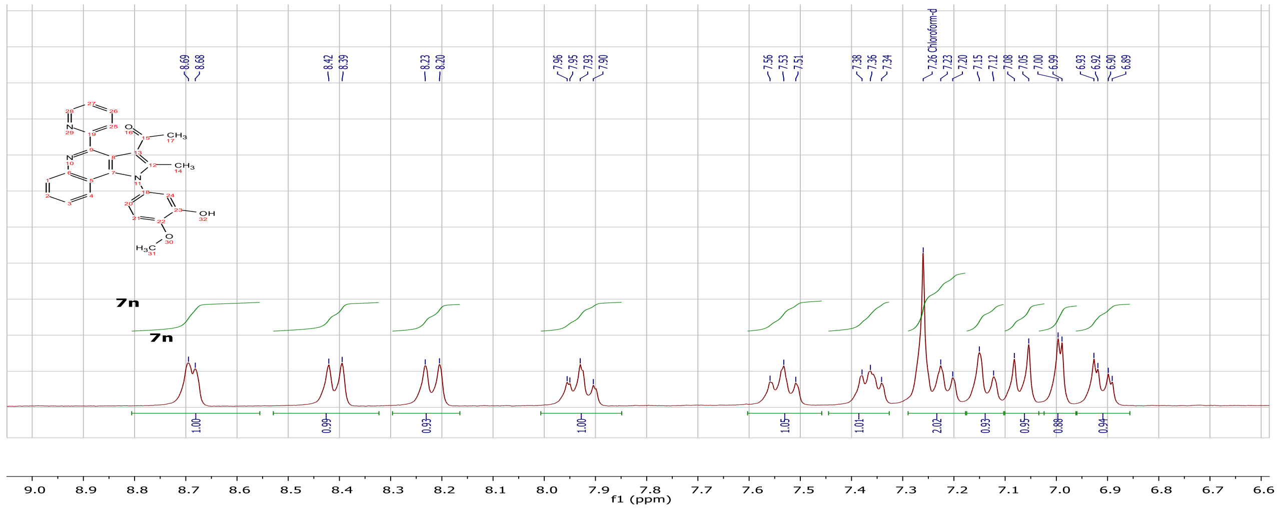

Figure S19. Focused ^{1}HNMR spectra for compound **7n**.

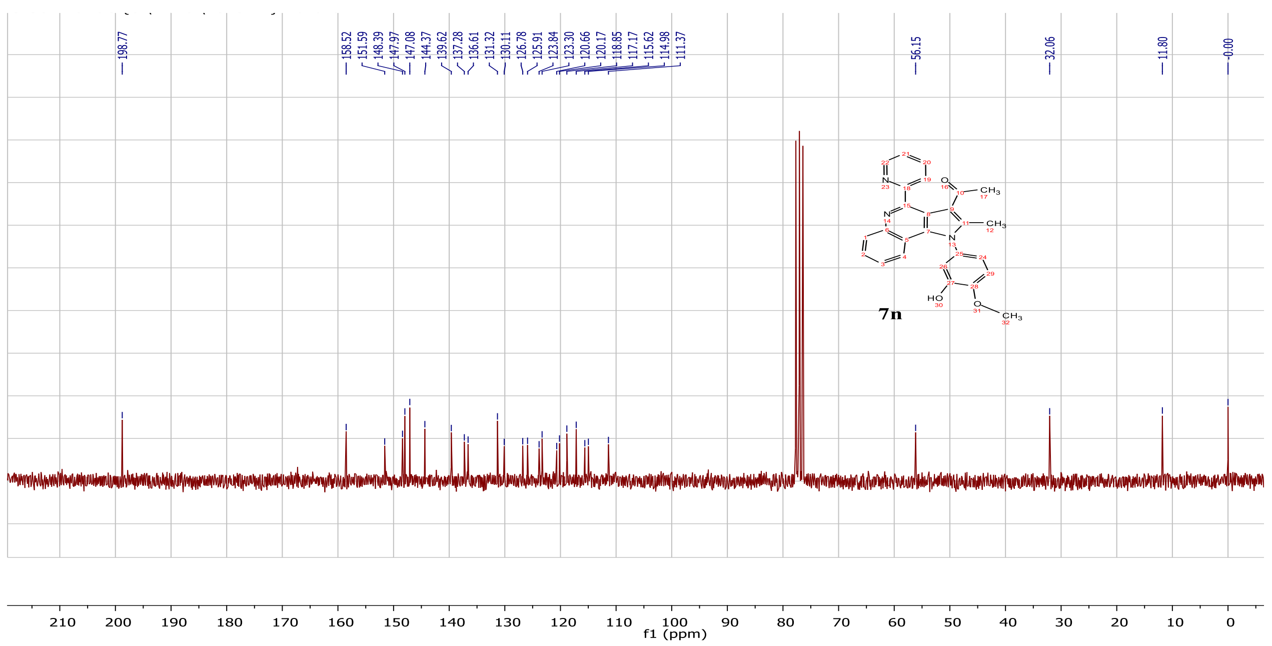

Figure S20. ^{13}CNMR spectra for compound **7n**.

7m

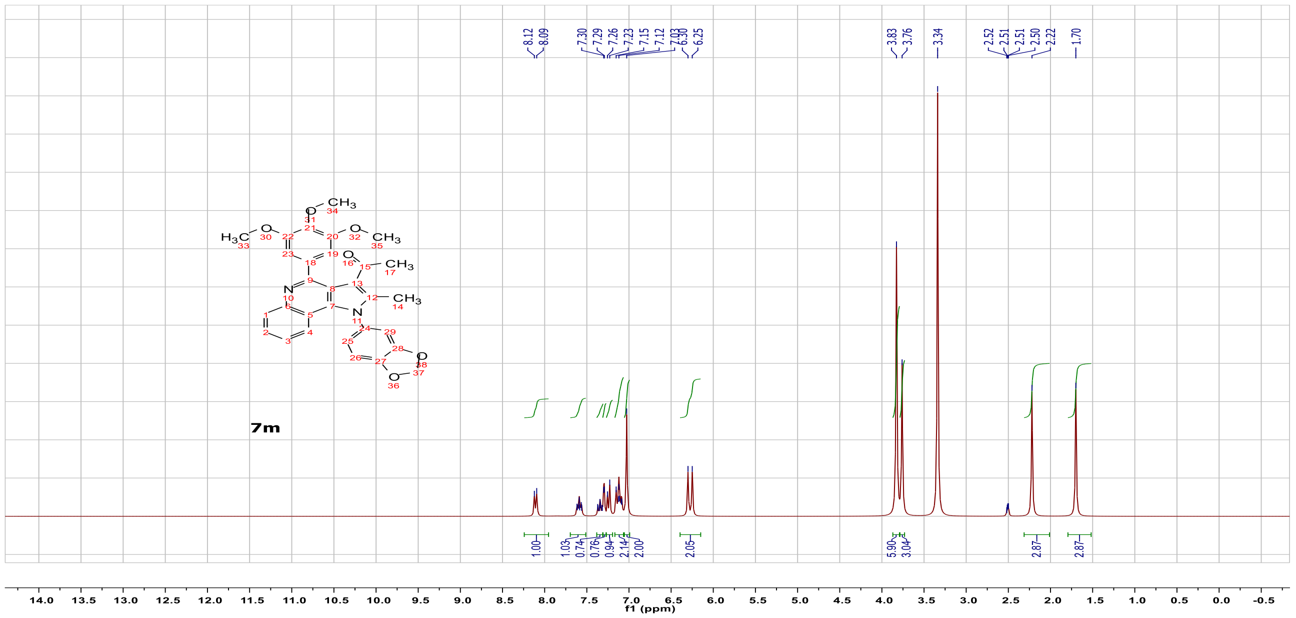

Figure S21. ^{1}HNMR spectra for compound **7m**.

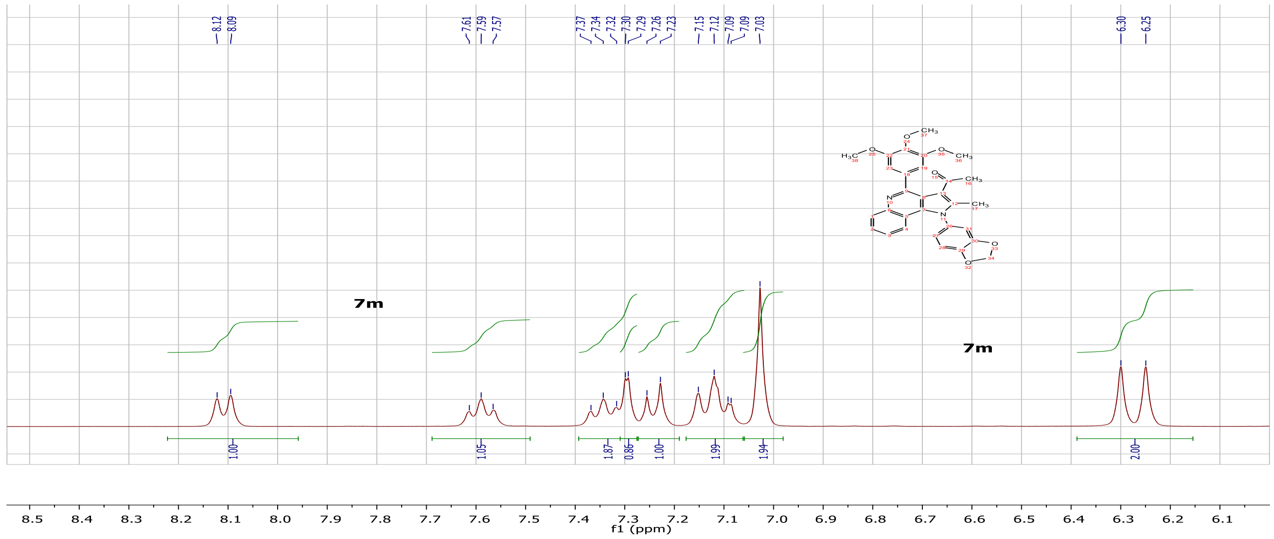

Figure S22. Focused ^{1}HNMR spectra for compound **7m**.

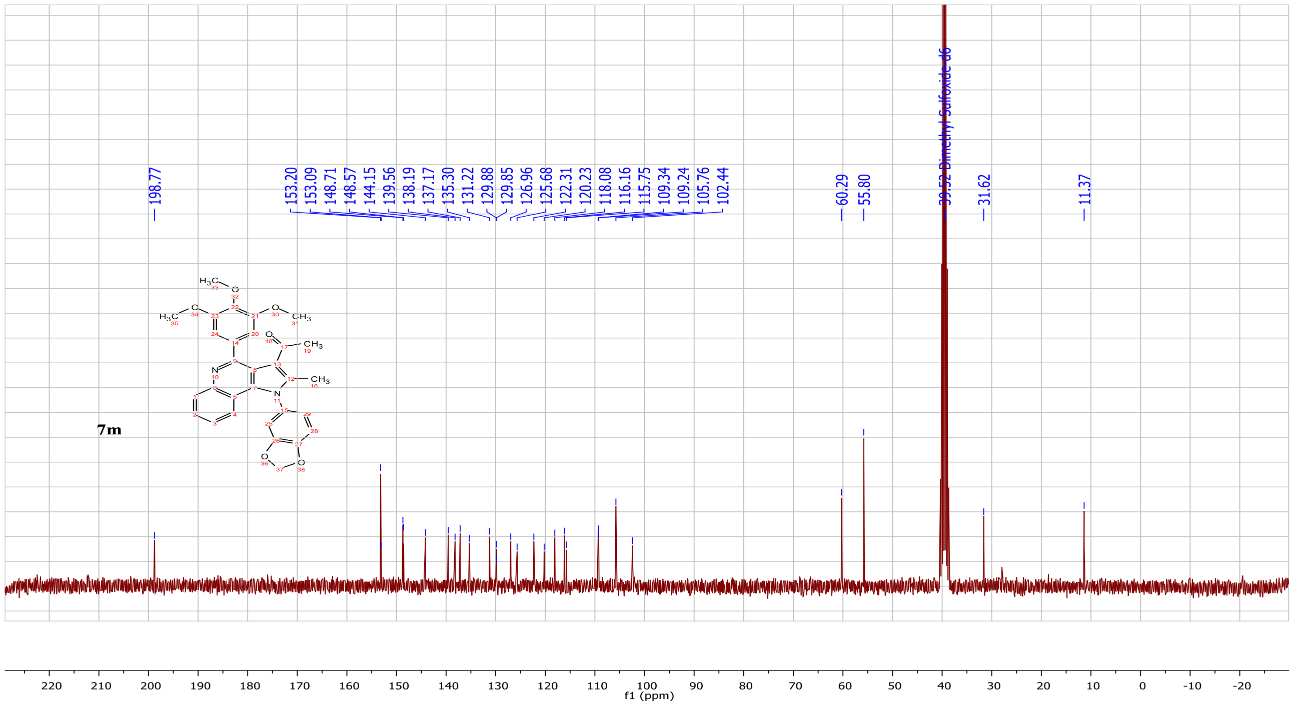

Figure S23. ^{13}CNMR spectra for compound **7m**.

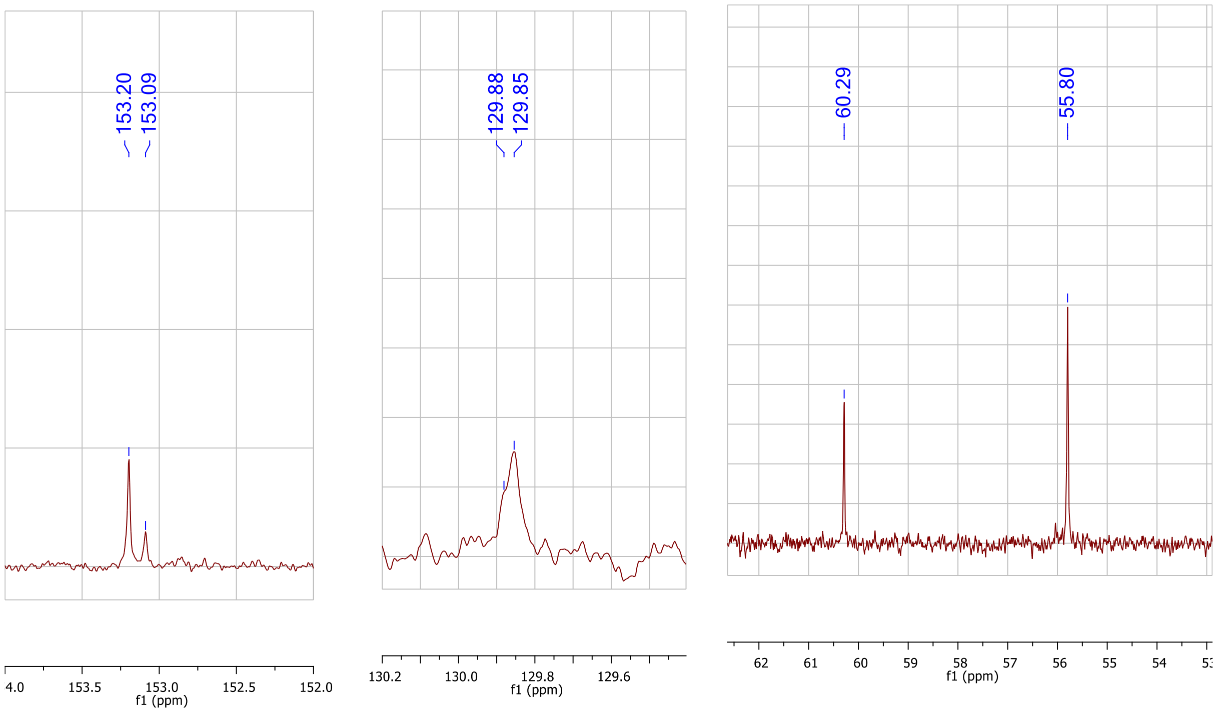

Figure S24. Focus on the most important spectra peaks for compound **7m** (153.20-153.09, 129.88-129.85, 60.29, 55.80).

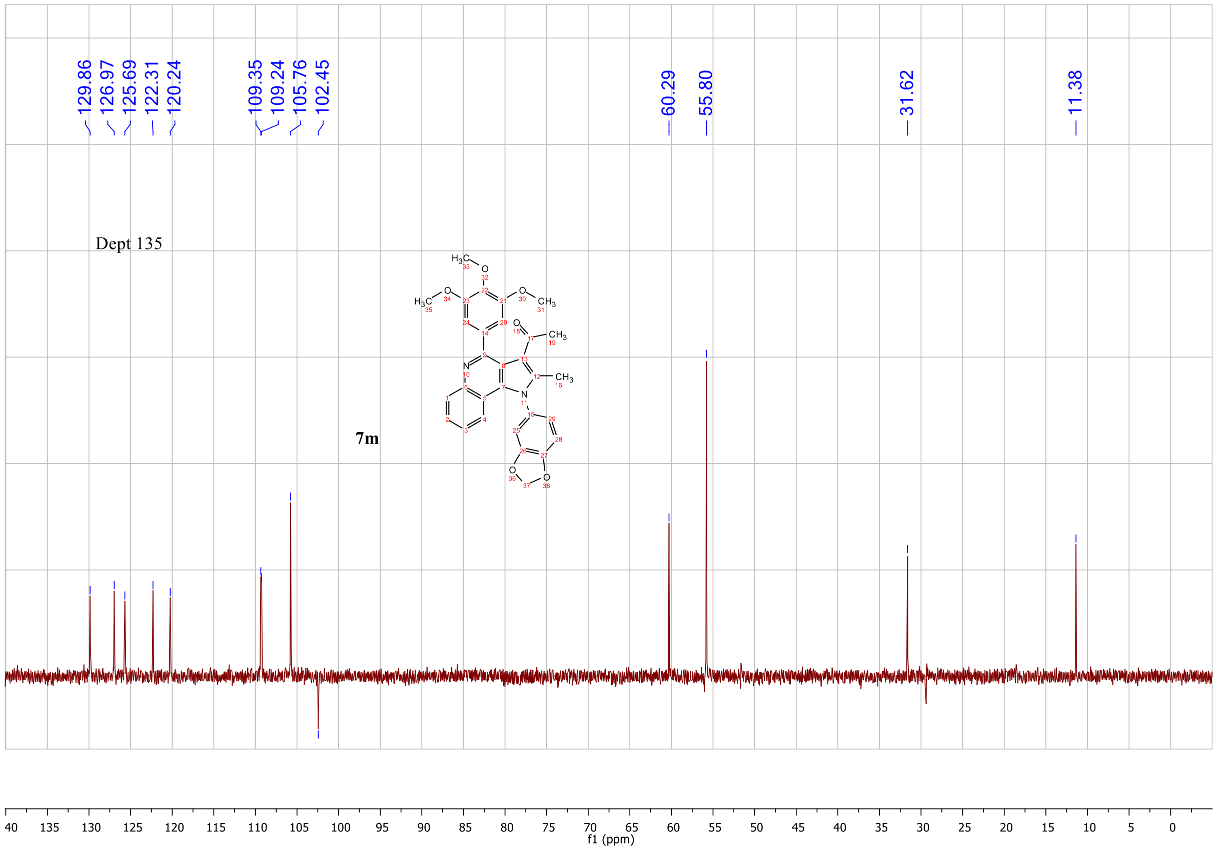

Figure S25. ^{13}CNMR DEPT spectra for compound **7m**.

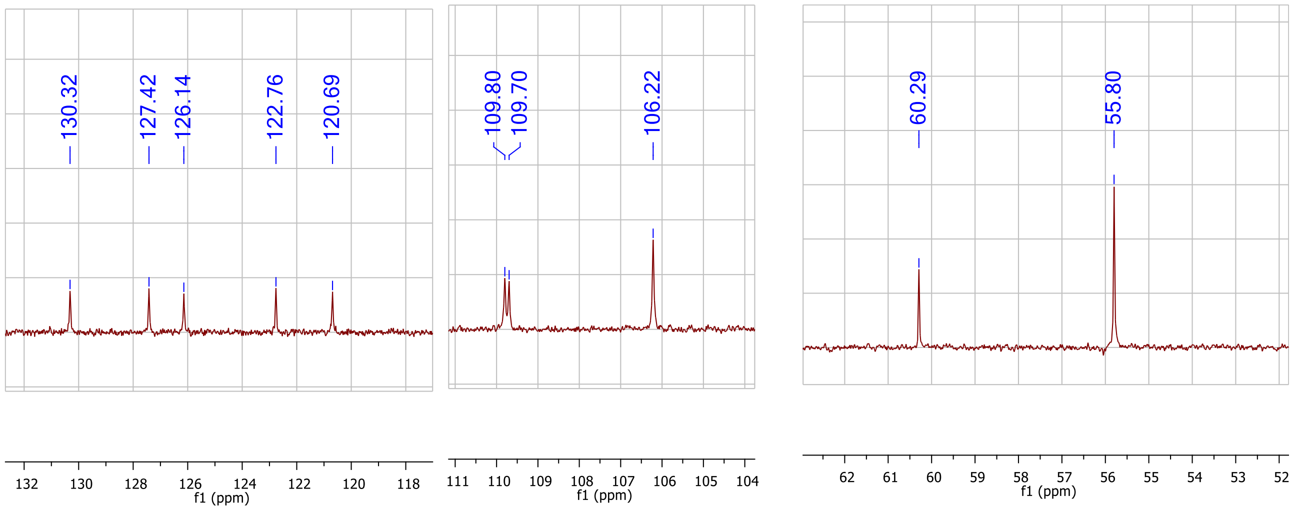

Figure S26. Focus on the most important spectra peaks for compound **7m** (130.32, 127.42-126.14, 122.76, 120.69, 109.80-109.70, 106.22, 60.29, 55.80).

7 l

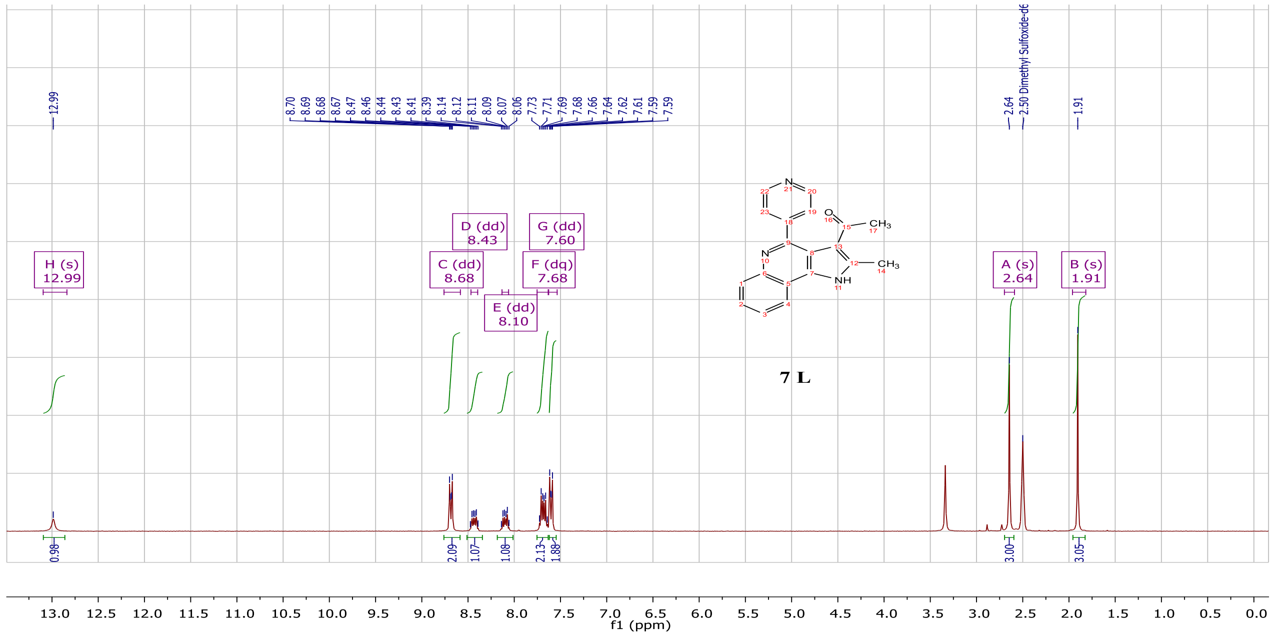

Figure S27. ^{1}HNMR spectra for compound **7l**.

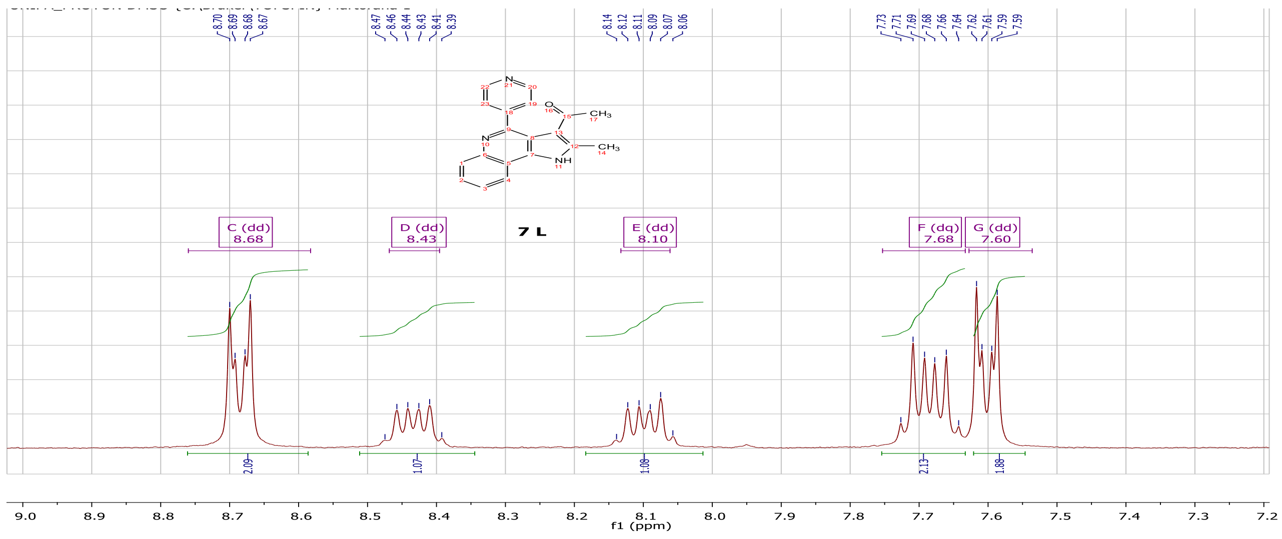

Figure S28. Focused ^{1}HNMR spectra for compound **7l**.

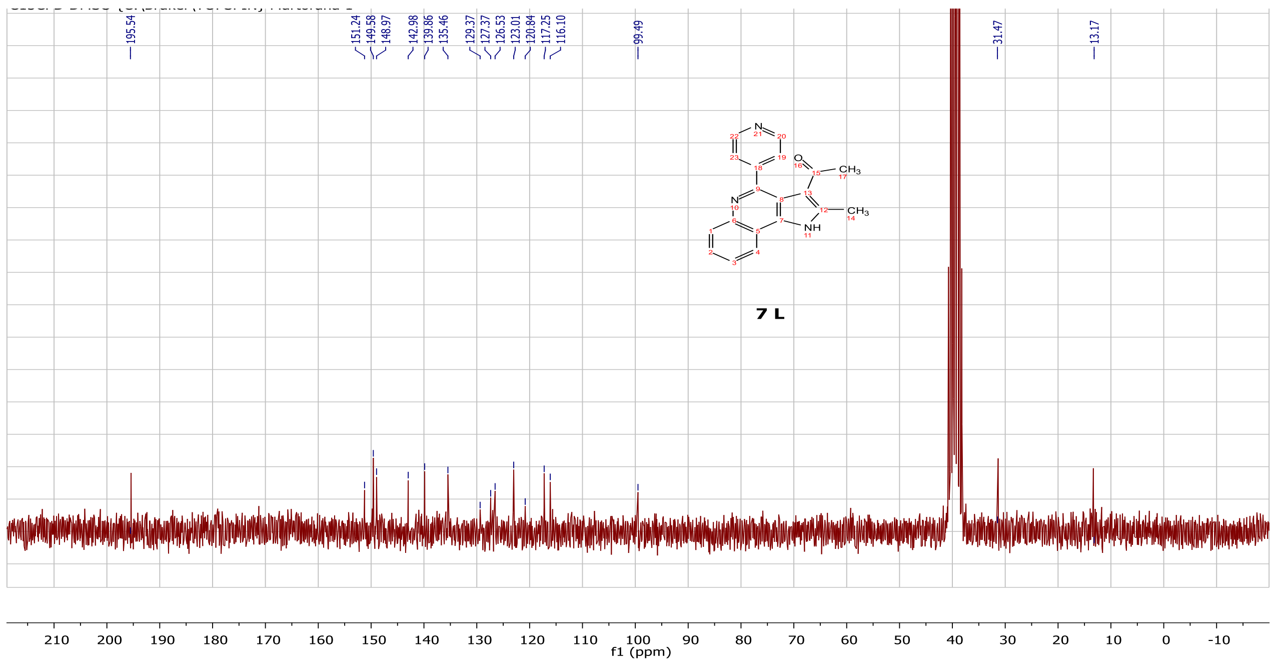

Figure S29. ^{13}CNMR spectra for compound **7l**.

7k

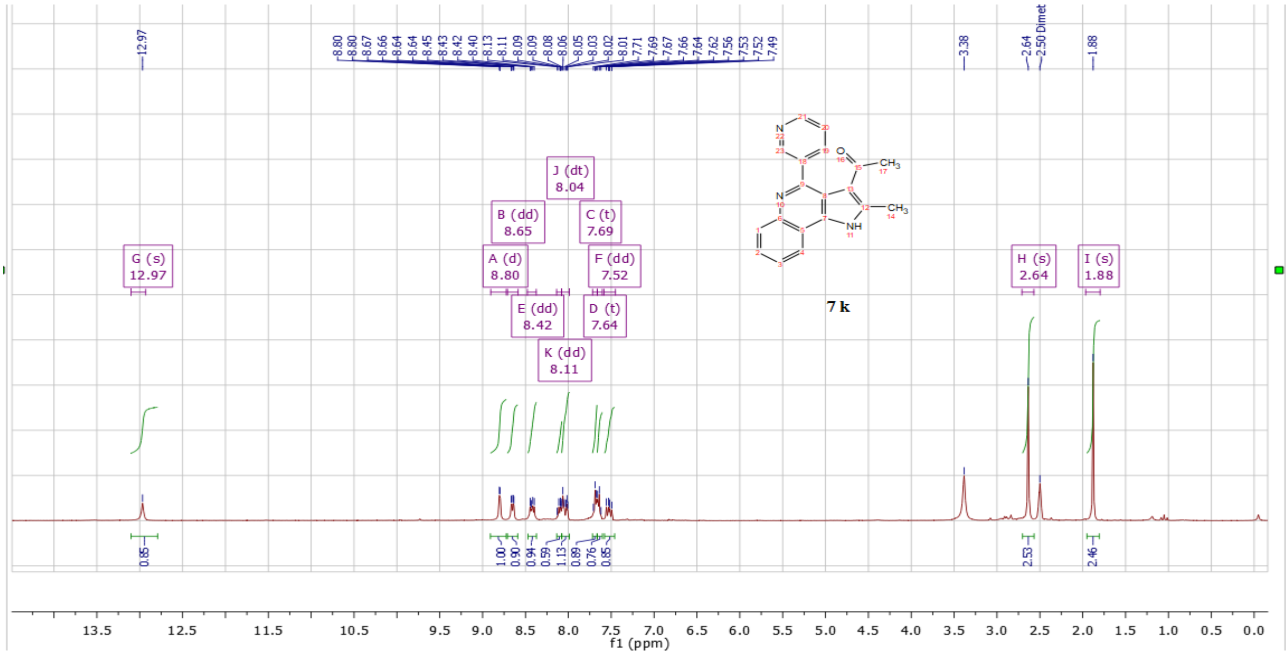

Figure S30. ^{1}HNMR spectra for compound **7k**.

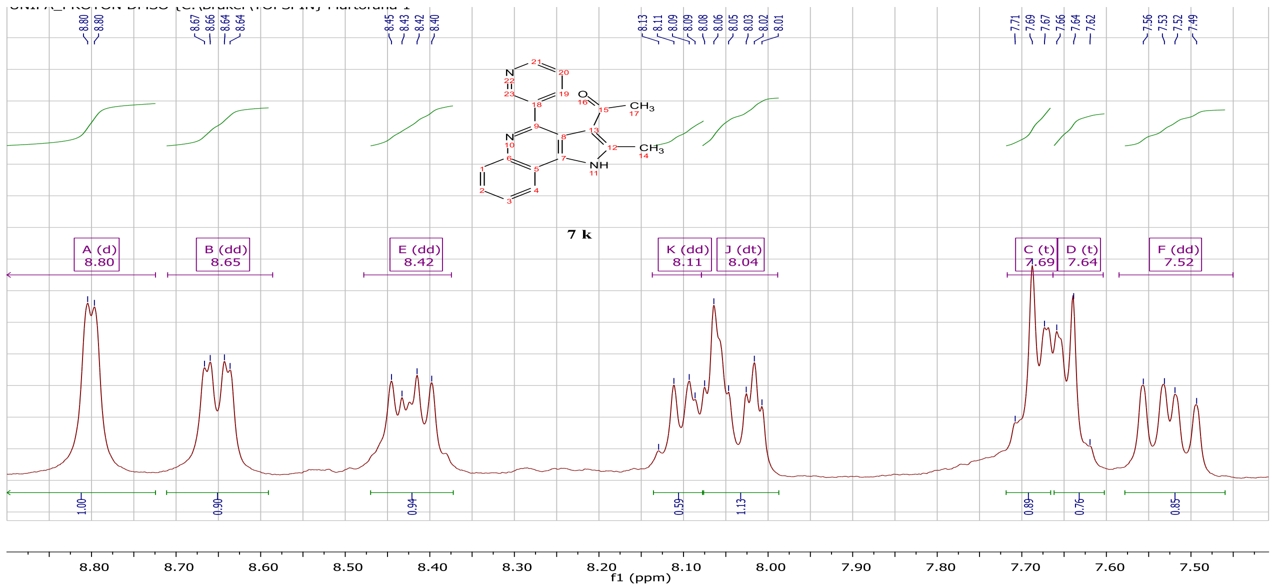

Figure S31. Focused ^{1}HNMR spectra for compound **7k**.

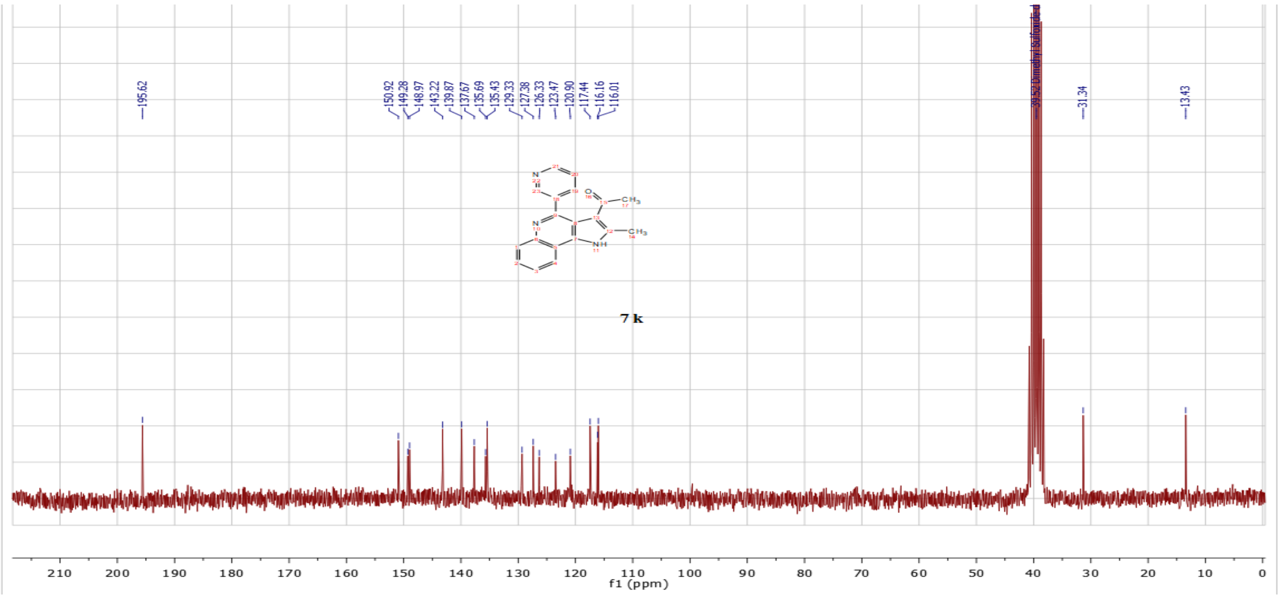

Figure S32. ^{13}CNMR spectra for compound **7k**.

7j

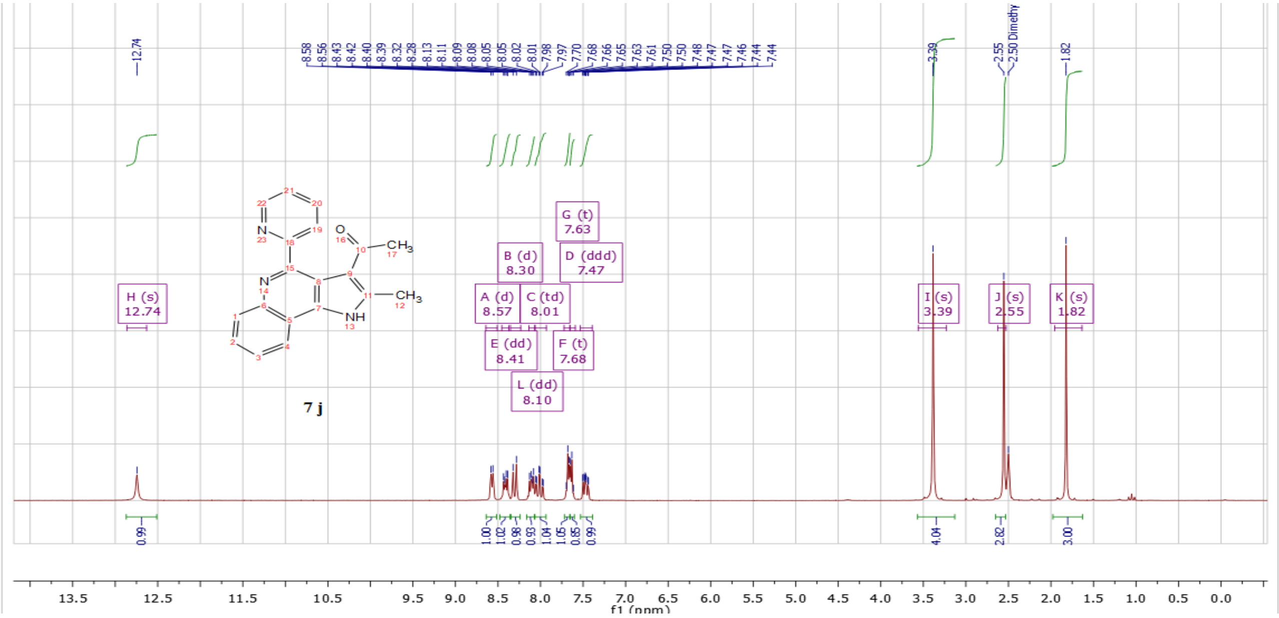

Figure S33. ^{1}HNMR spectra for compound **7j**.

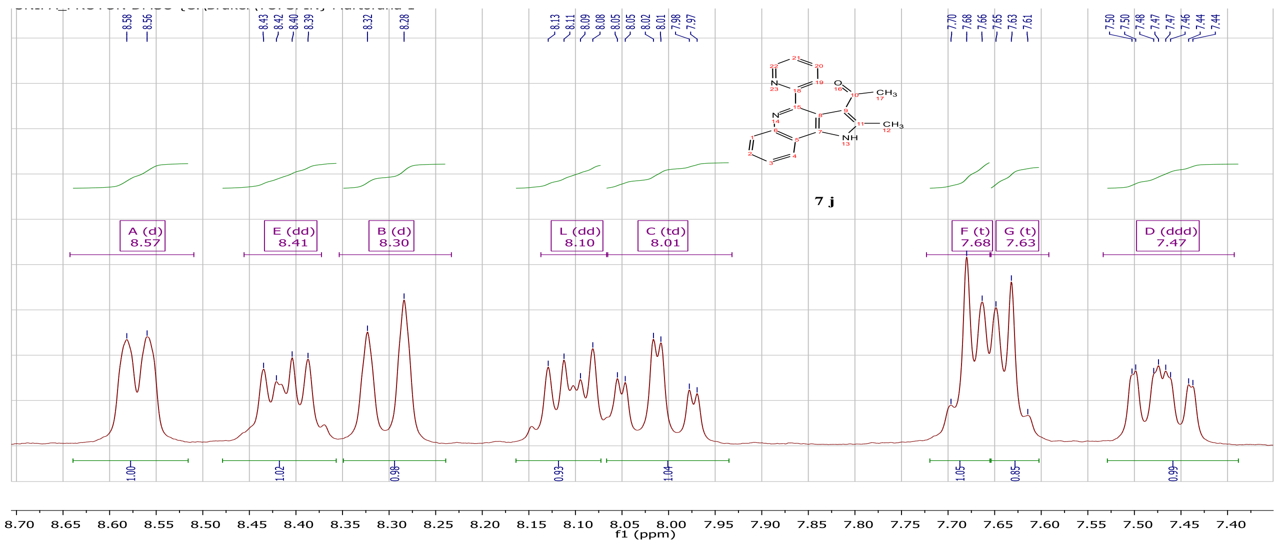

Figure S34. Focused ^{1}HNMR spectra for compound **7j**.

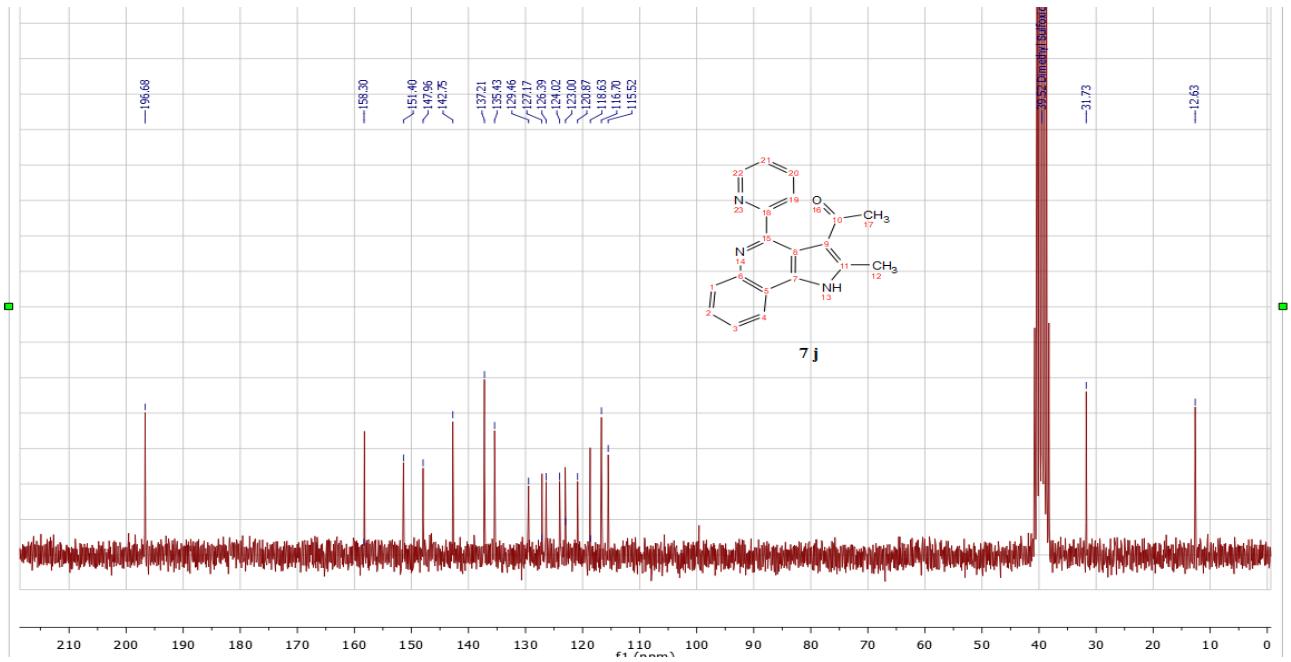

Figure S35. ^{13}CNMR spectra for compound **7j**.

7i

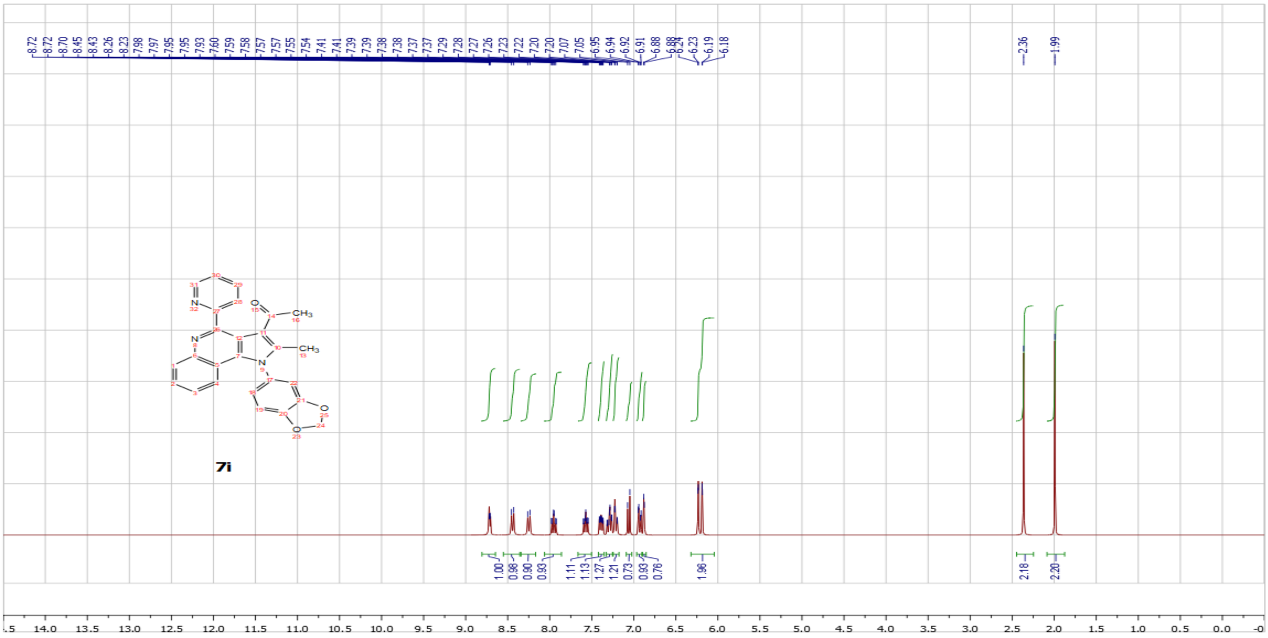

Figure S36. ^{1}HNMR spectra for compound **7i**.

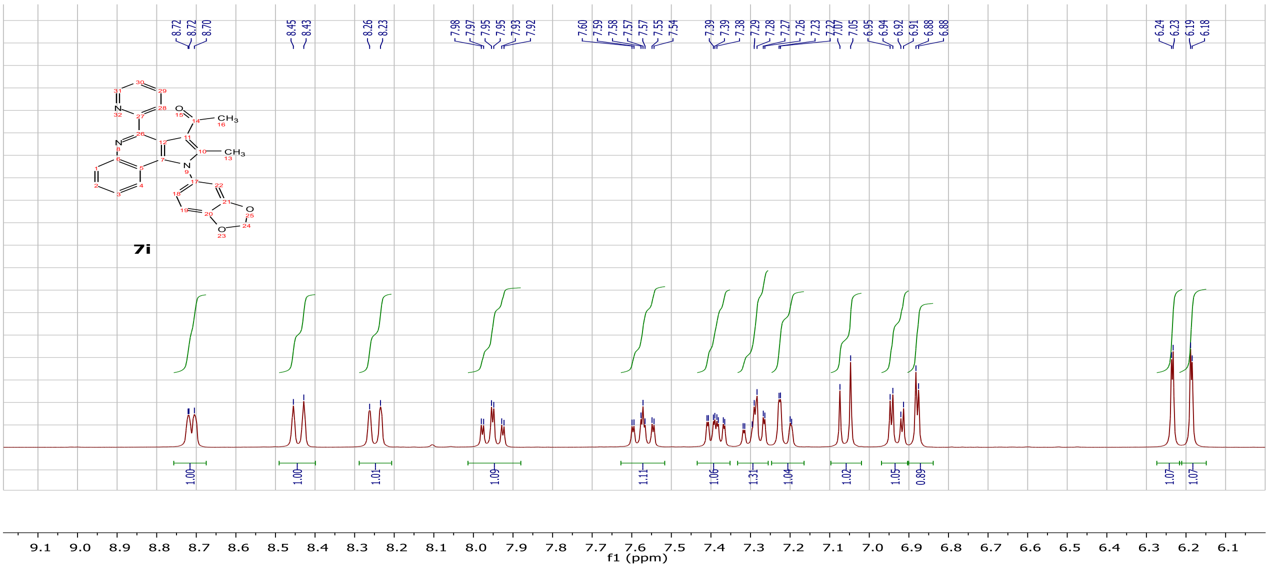

Figure S37. Focused ^{1}HNMR spectra for compound **7i**.

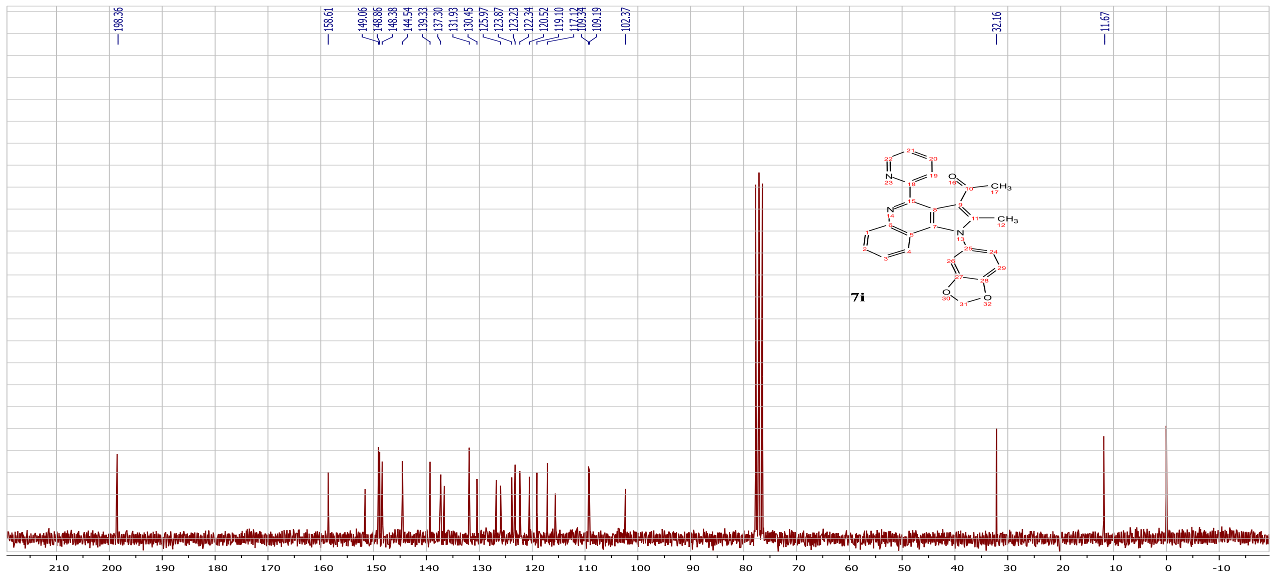

Figure S38. ^{13}CNMR spectra for compound **7i**.

7h

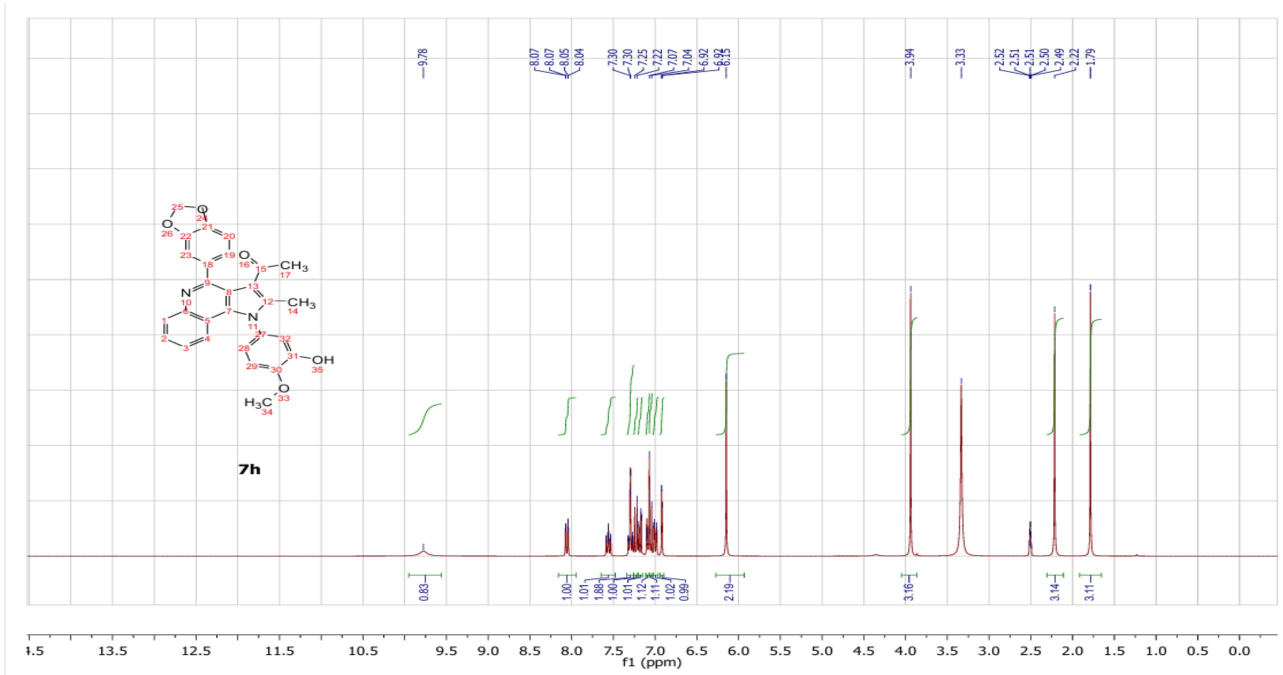

Figure S39. ^{1}HNMR spectra for compound **7h**.

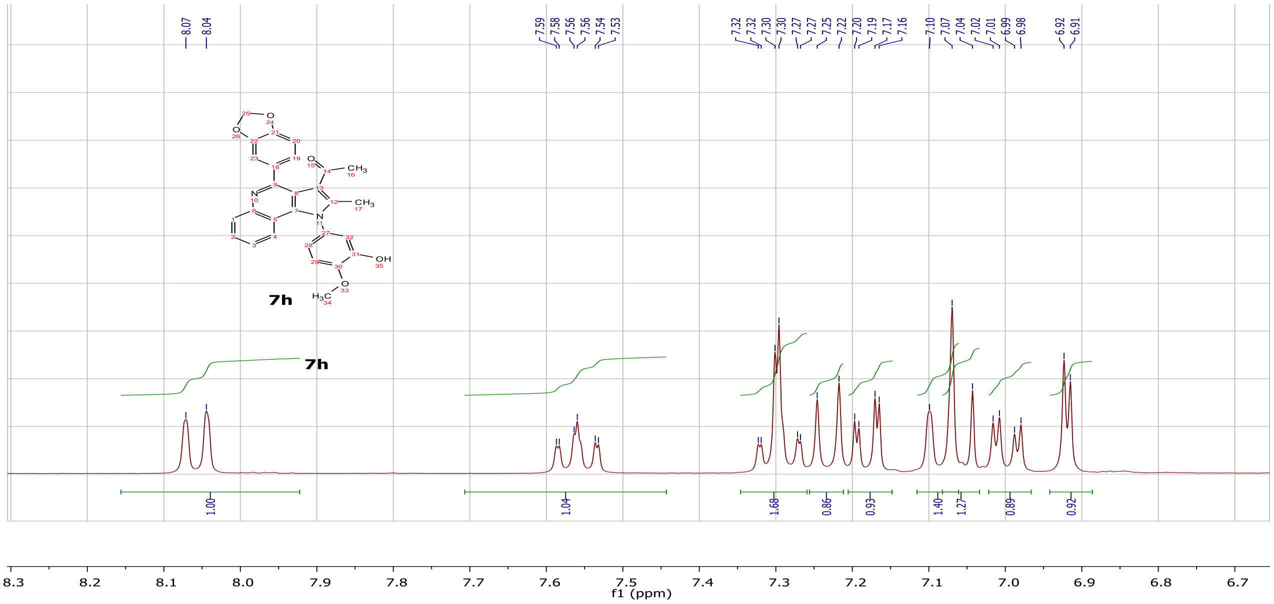

Figure S40. Focused ^{1}HNMR spectra for compound **7h**.

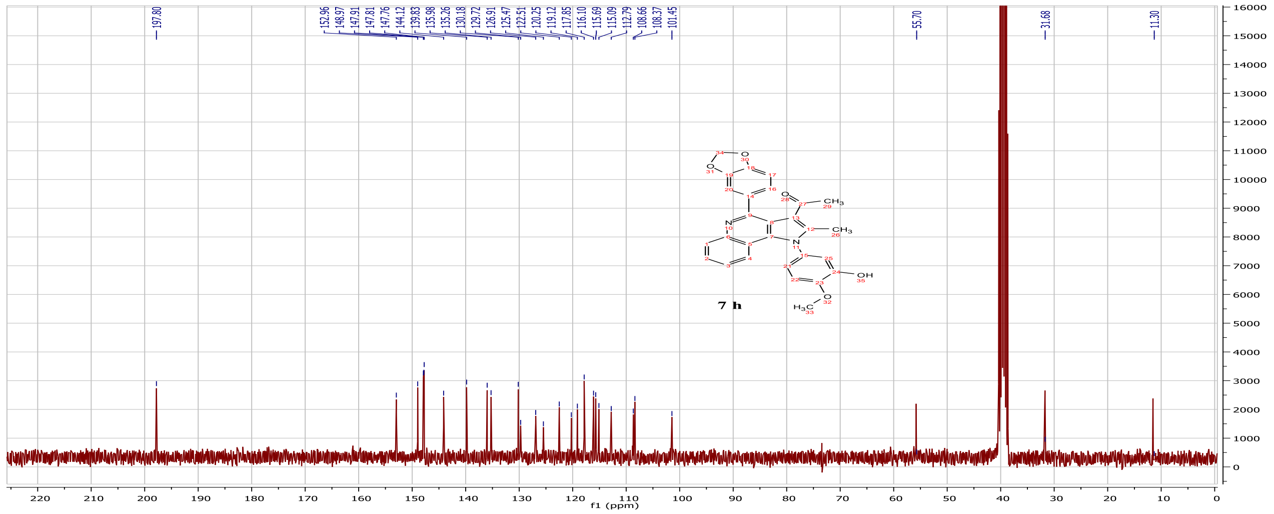

Figure S41. ^{13}CNMR spectra for compound **7h**.

7g

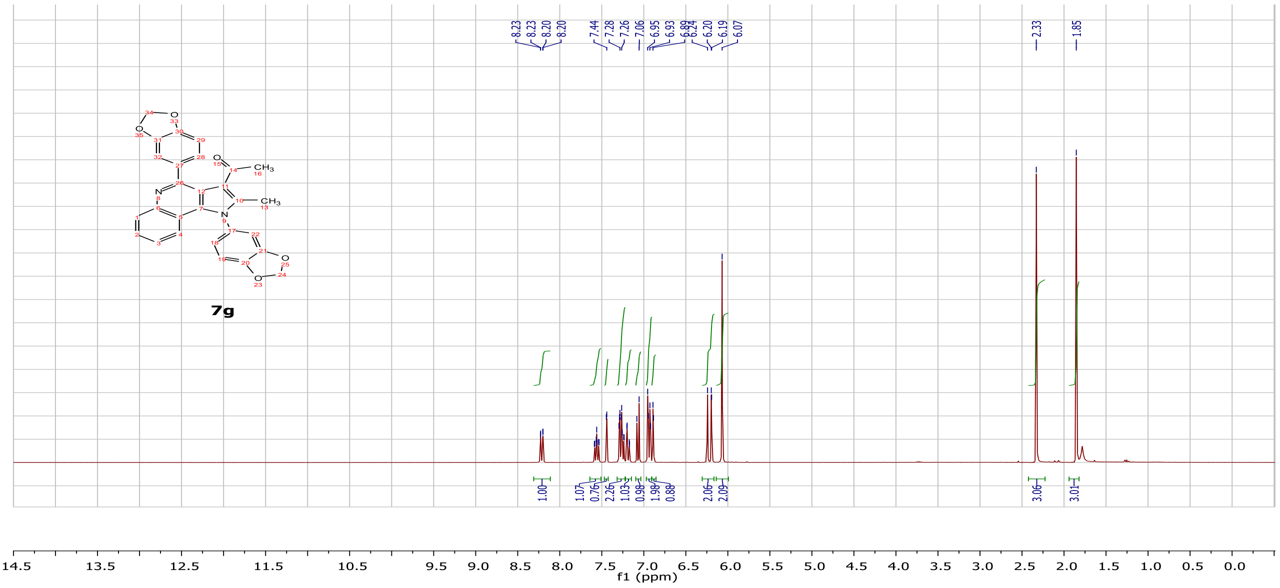

Figure S42. ^{1}HNMR spectra for compound **7g**.

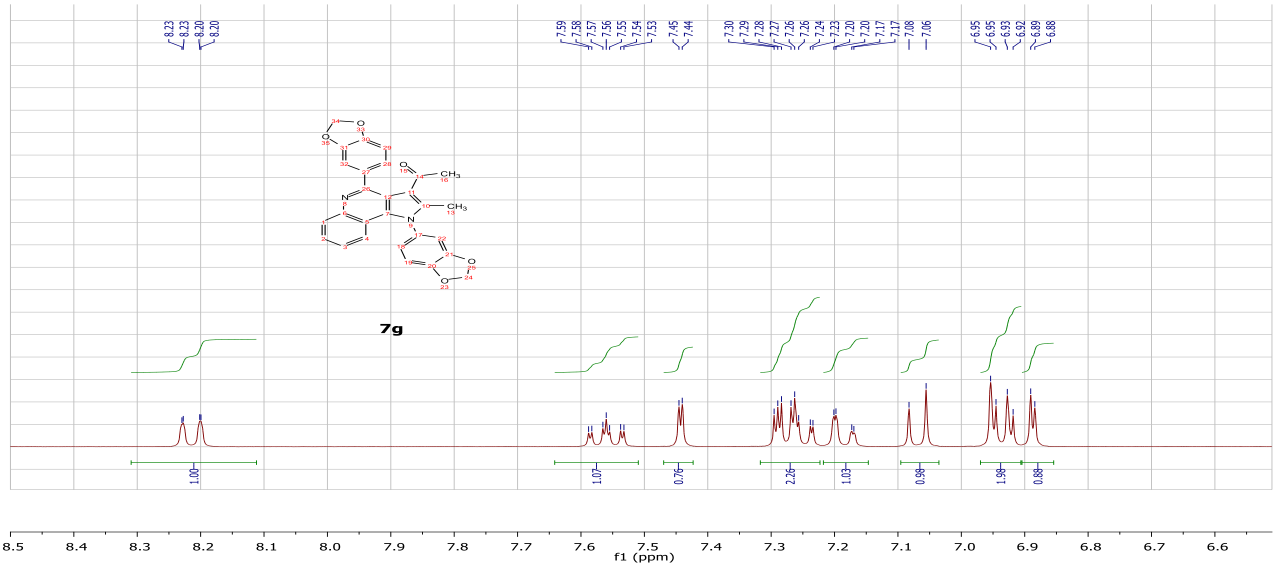

Figure S43. Focused ^{1}HNMR spectra for compound **7g**.

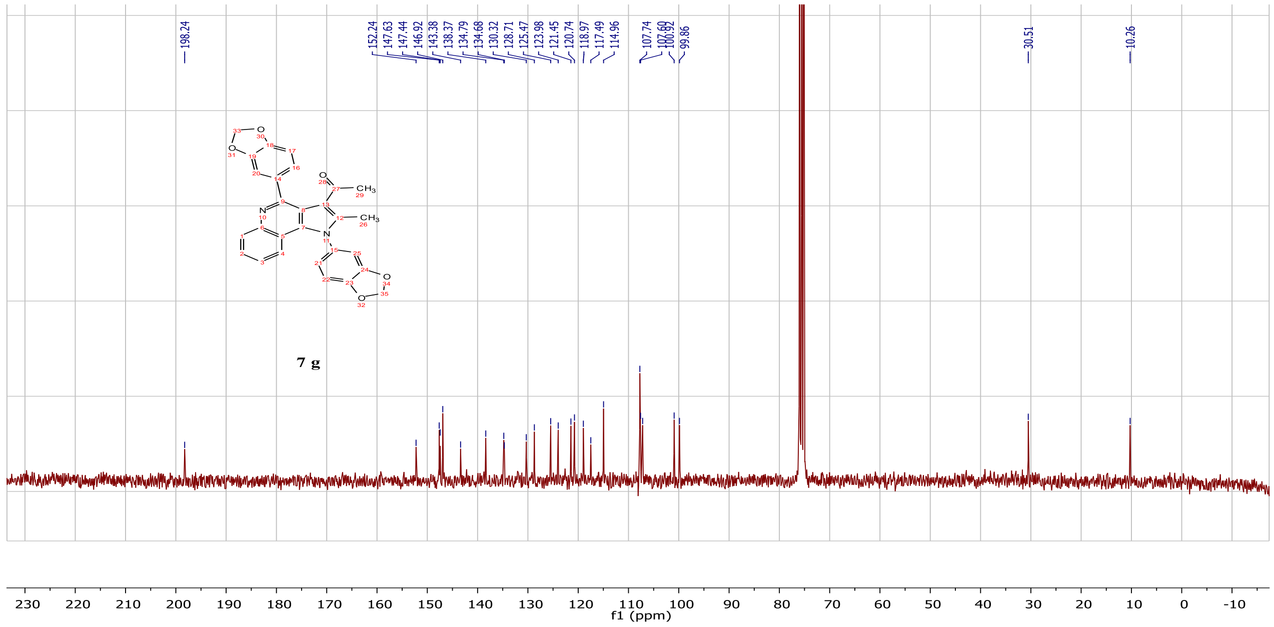

Figure S44. ^{13}CNMR spectra for compound **7g**.

7f

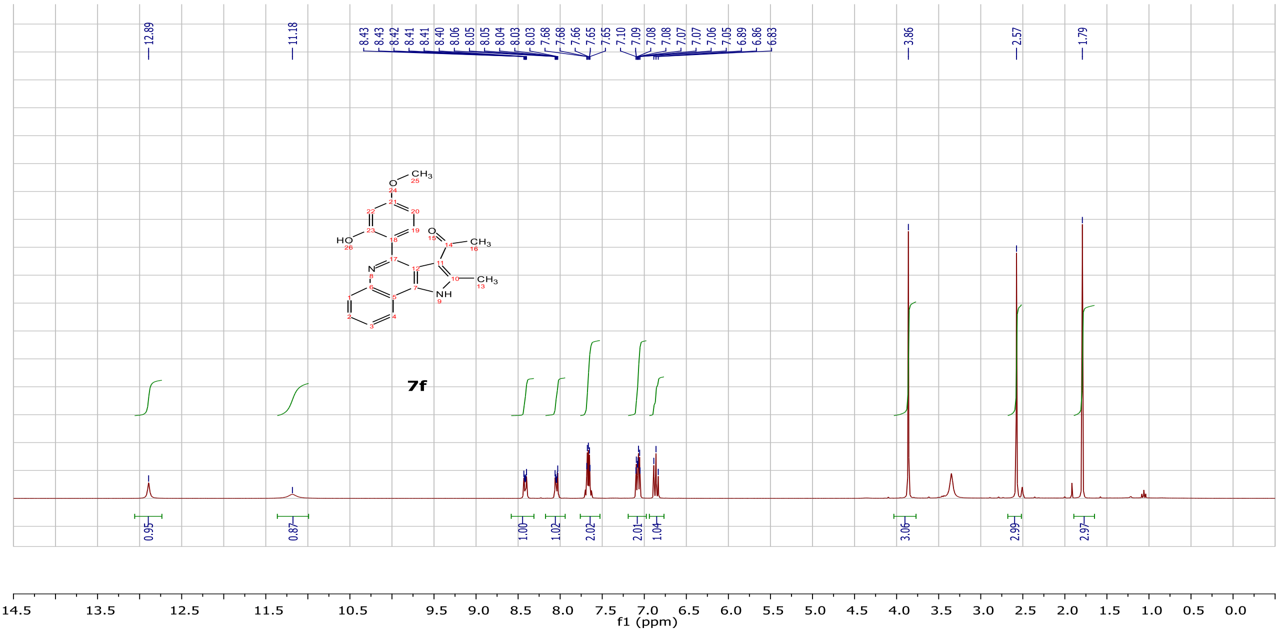

Figure S45. ^{1}HNMR spectra for compound **7f**.

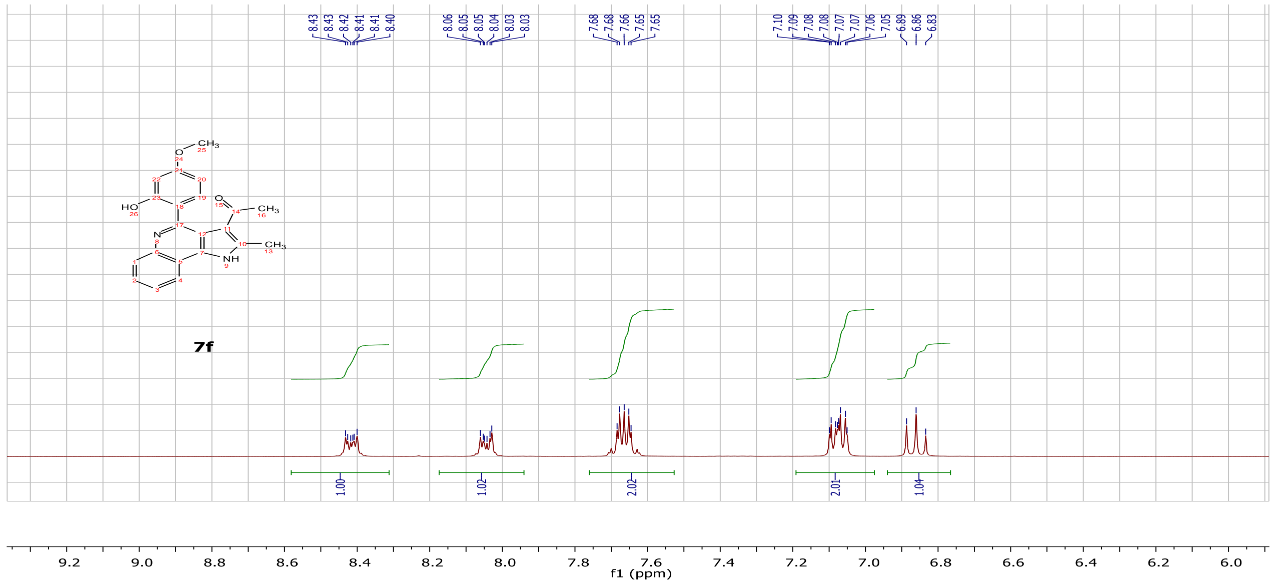

Figure S46. Focused ^{1}HNMR spectra for compound **7f**.

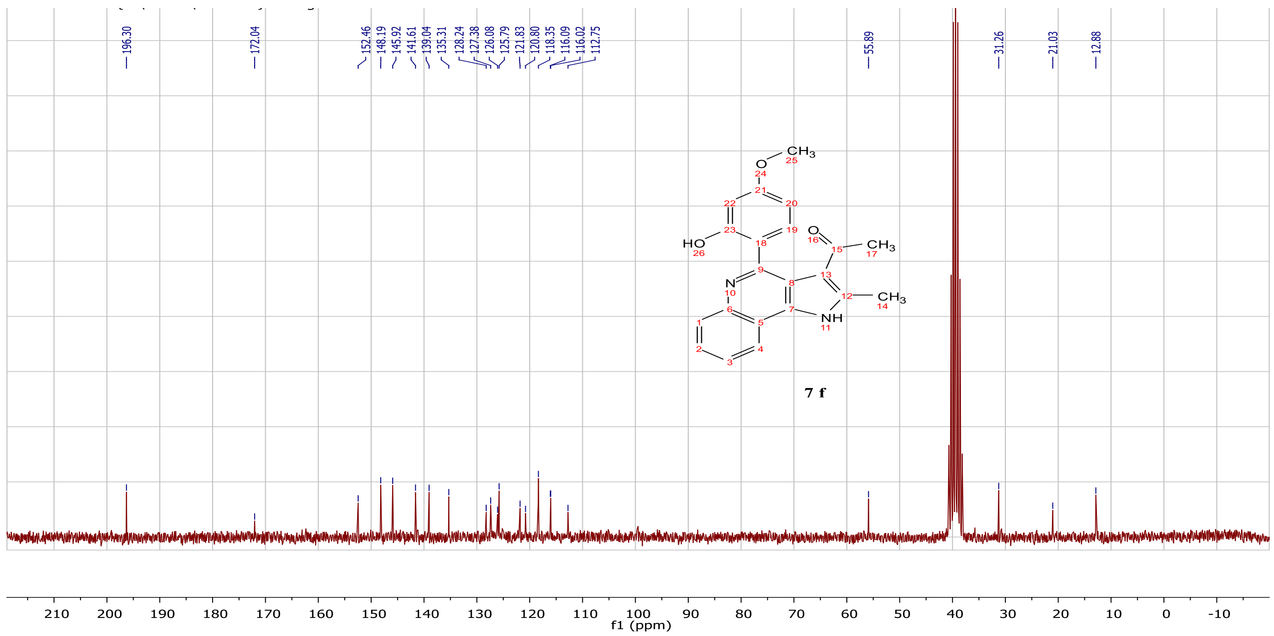

Figure S47. ^{13}CNMR spectra for compound **7f**.

7e

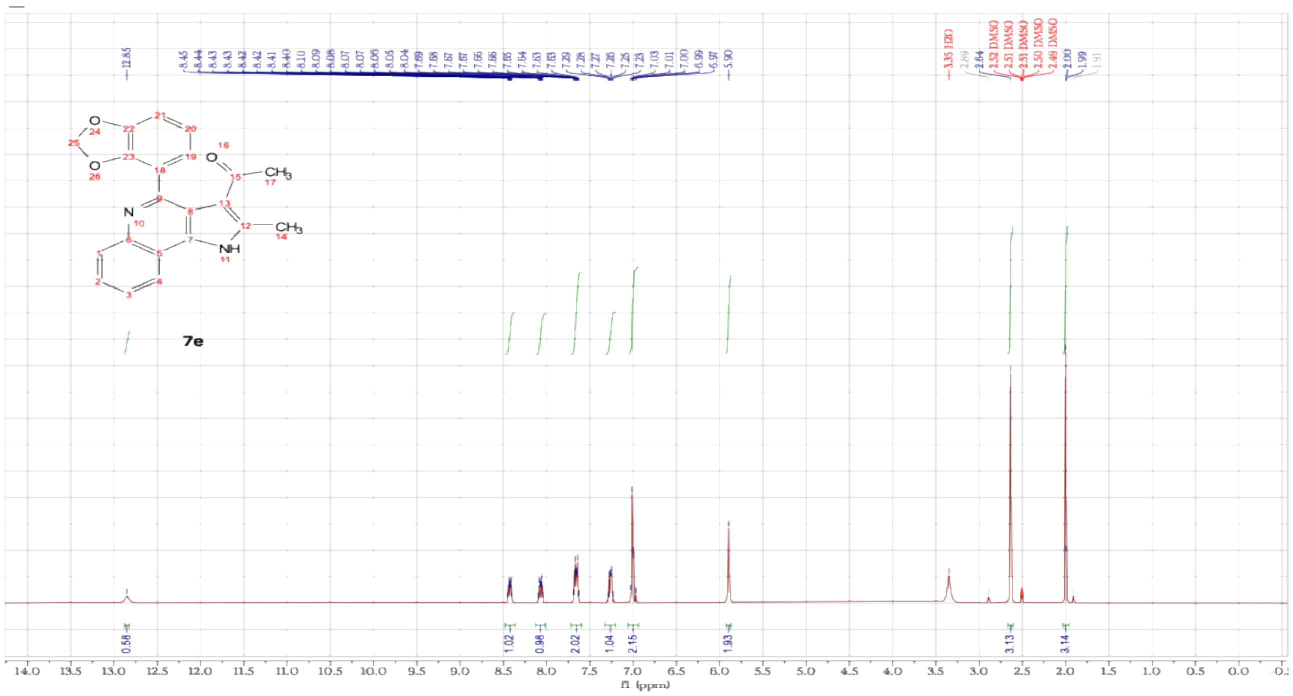

Figure S48. ^{1}HNMR spectra for compound **7e**.

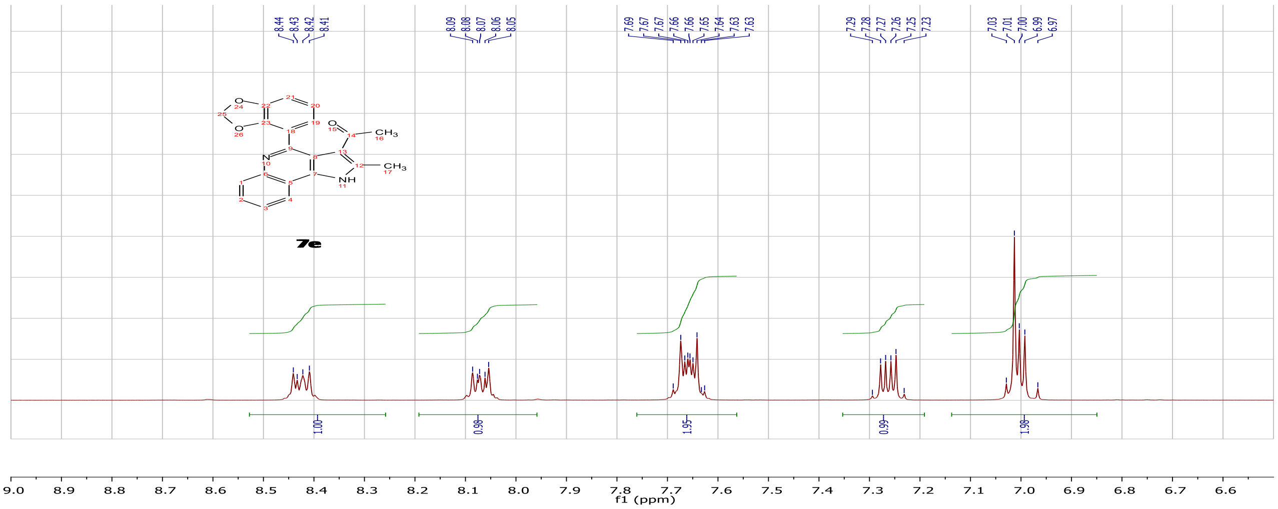

Figure S49. Focused ^{1}HNMR spectra for compound **7e**.

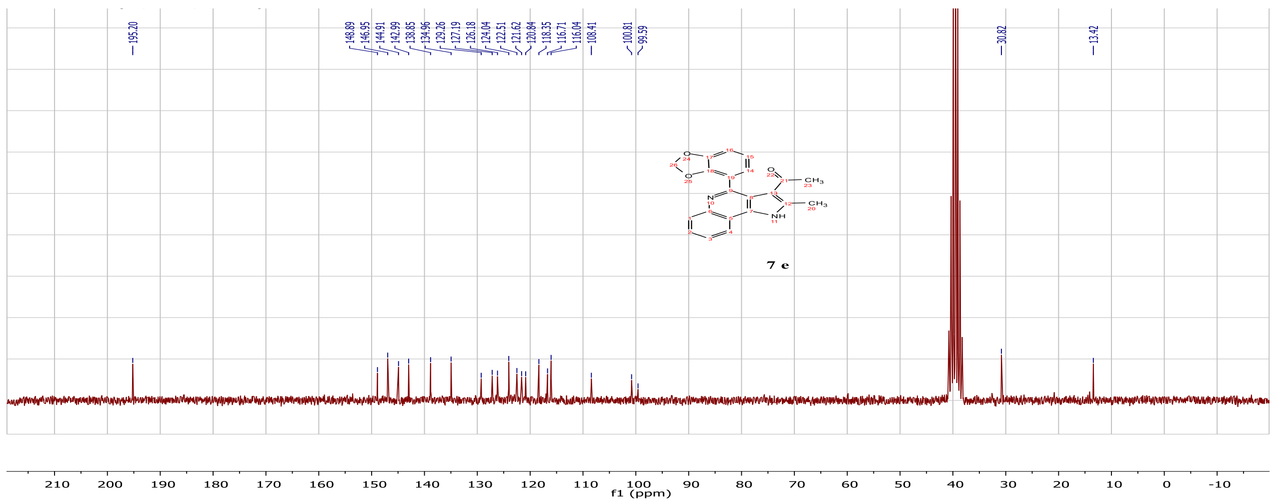

Figure S50. ^{13}CNMR spectra for compound **7e**.

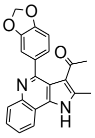

7d (F. Mingoia, C. Di Sano, C. D'Anna, M. Fazzari, L. Minafra, A. Bono, G. La Monica, A. Martorana, A. M. Almerico, A. Lauria. *Eur. J. of Med. Chem.*, **2023**, 258, 115537. https://doi.org/10.1016/j.ejmech.2023.115537)

3c

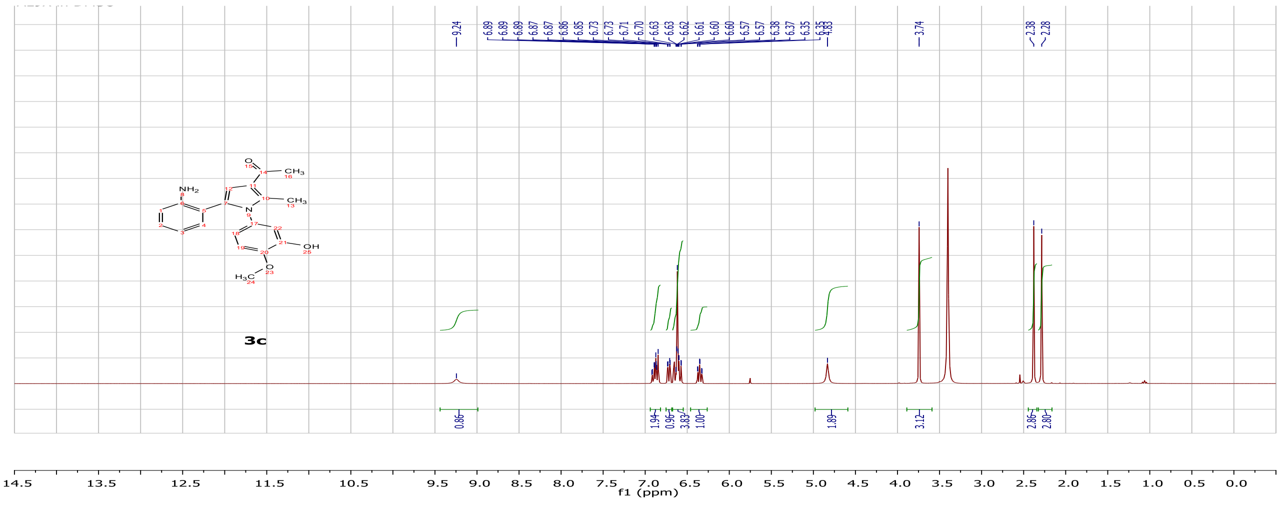

Figure S51. ^{1}HNMR spectra for compound **3c**.

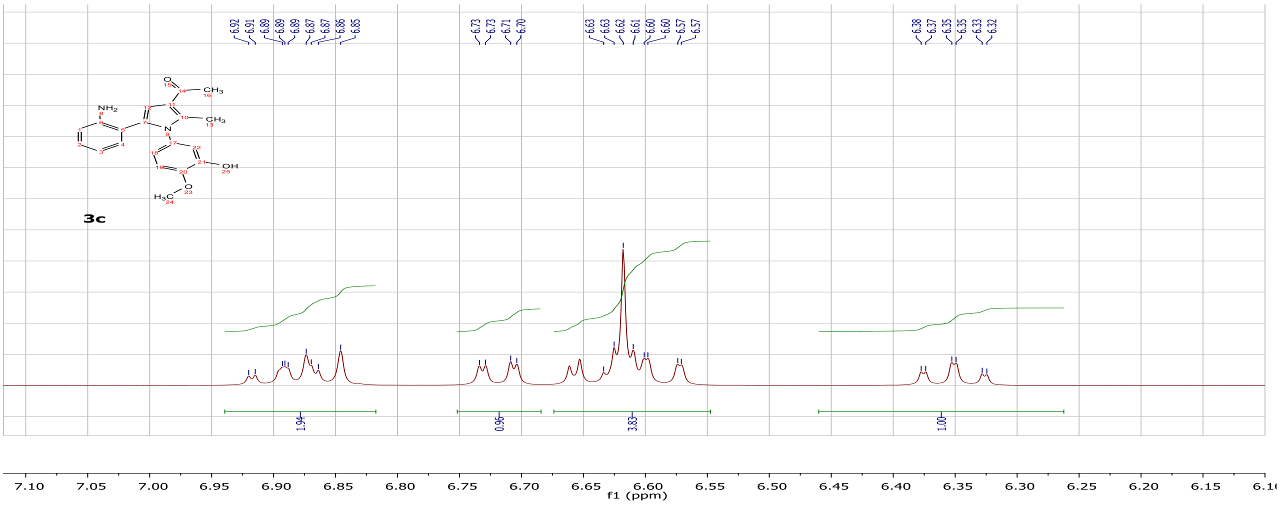

Figure S52. Focused ^{1}HNMR spectra for compound **3c**.

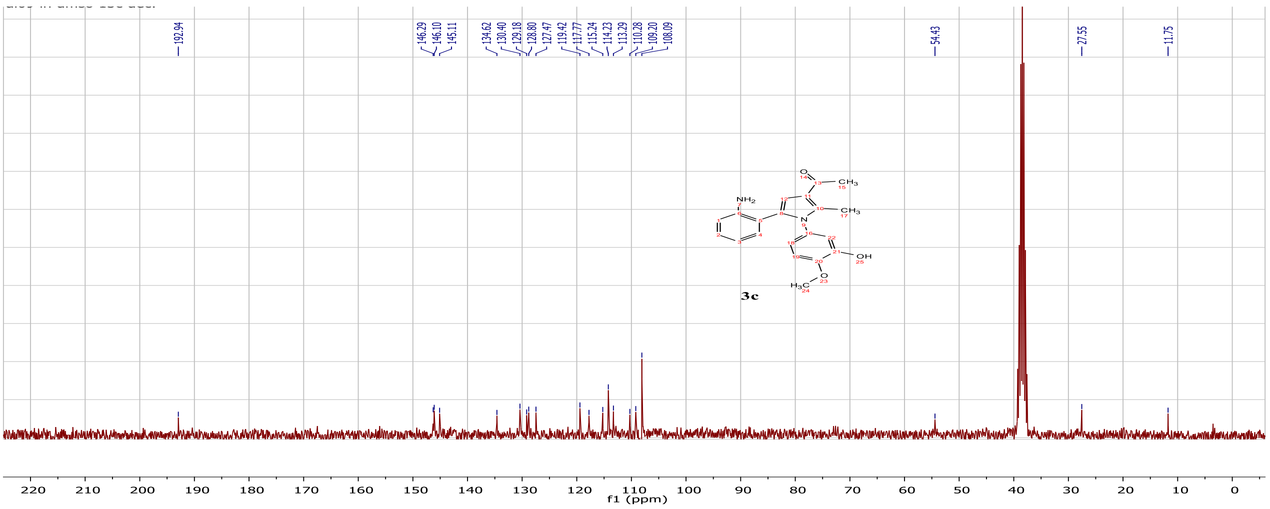

Figure S53. ^{13}CNMR spectra for compound **3c**.

3b

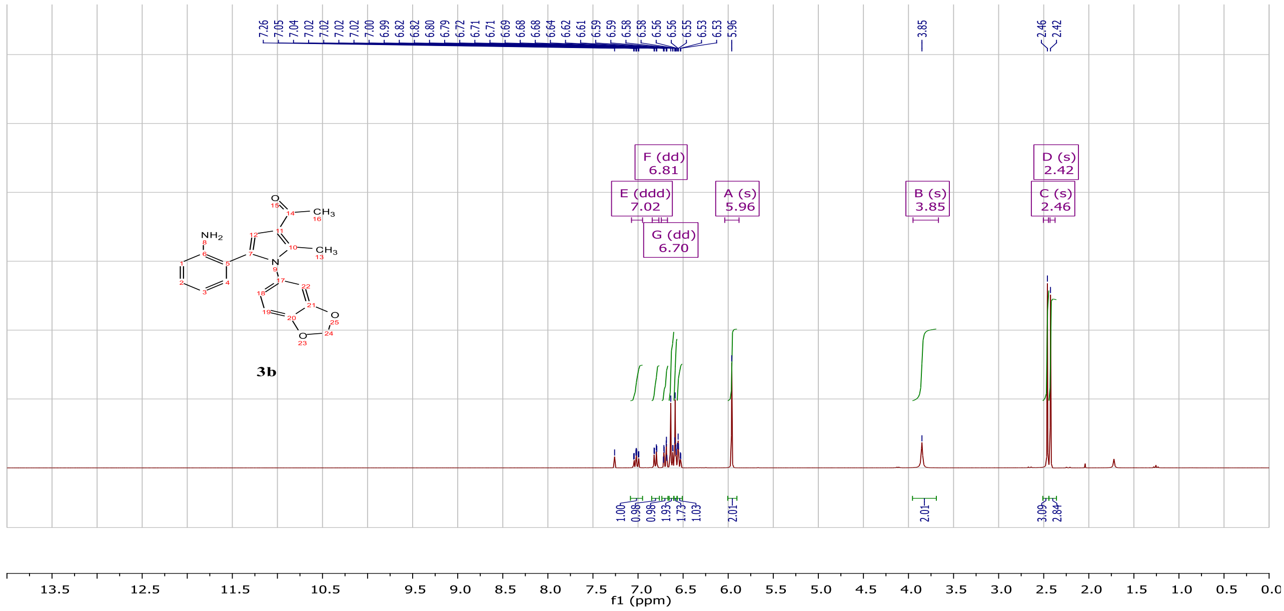

Figure S54. ^{1}HNMR spectra for compound **3b**.

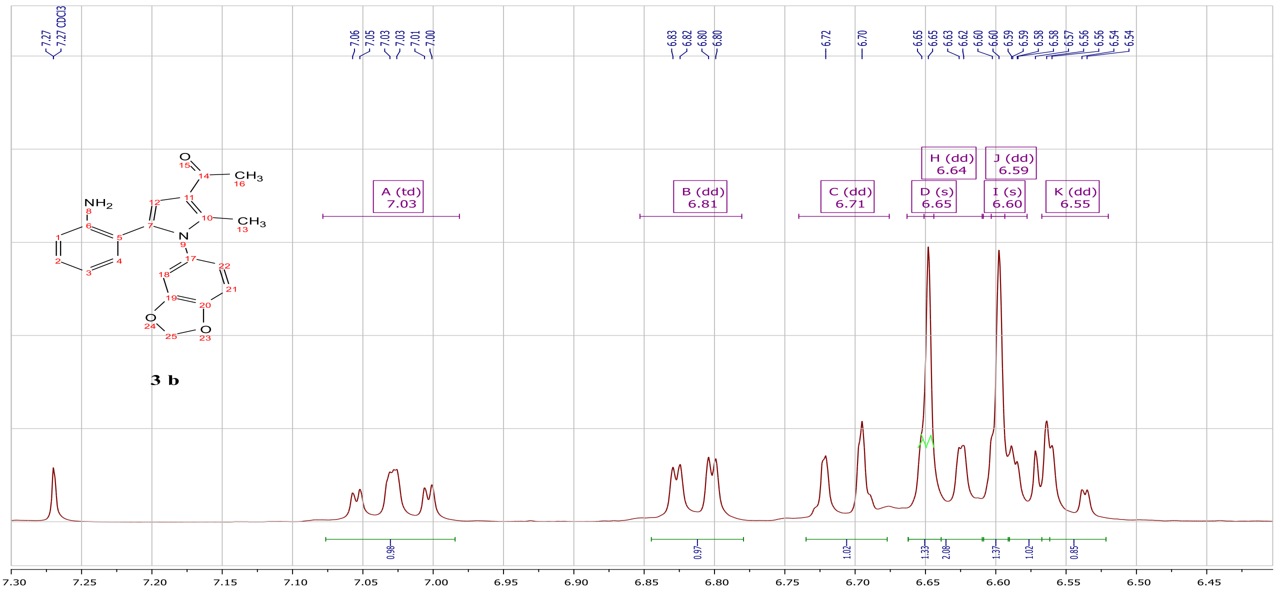

Figure S55. Focused ^{1}HNMR spectra for compound **3b**.

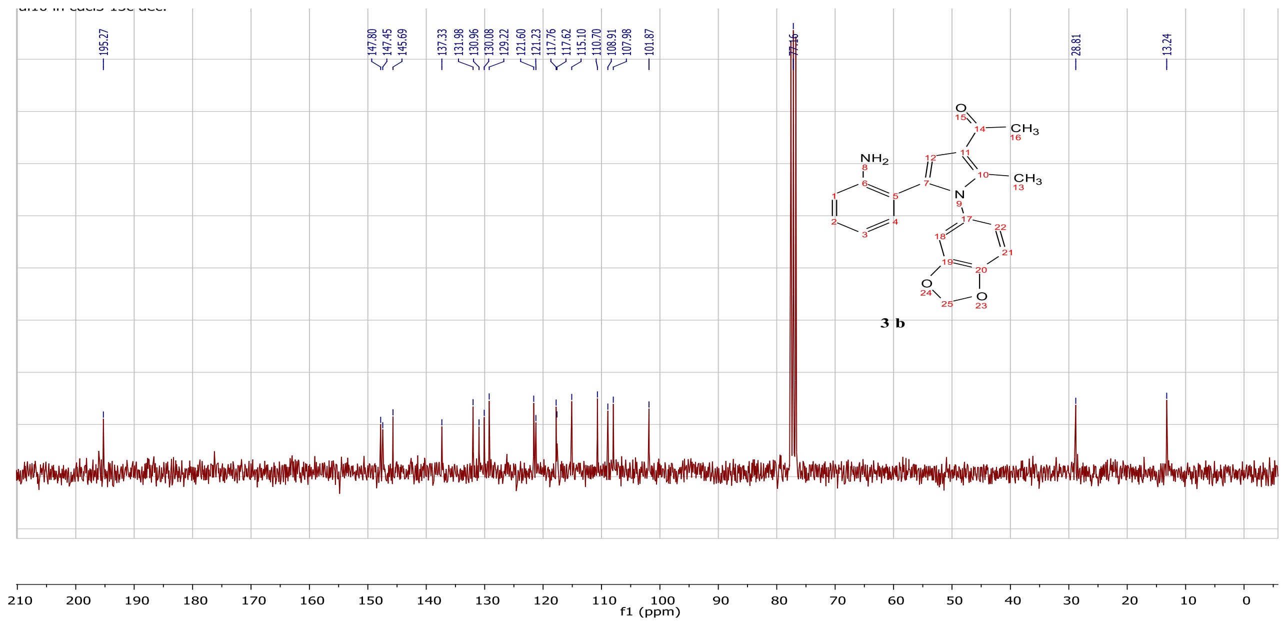

Figure S56. ^{13}CNMR spectra for compound **3b**.

2c

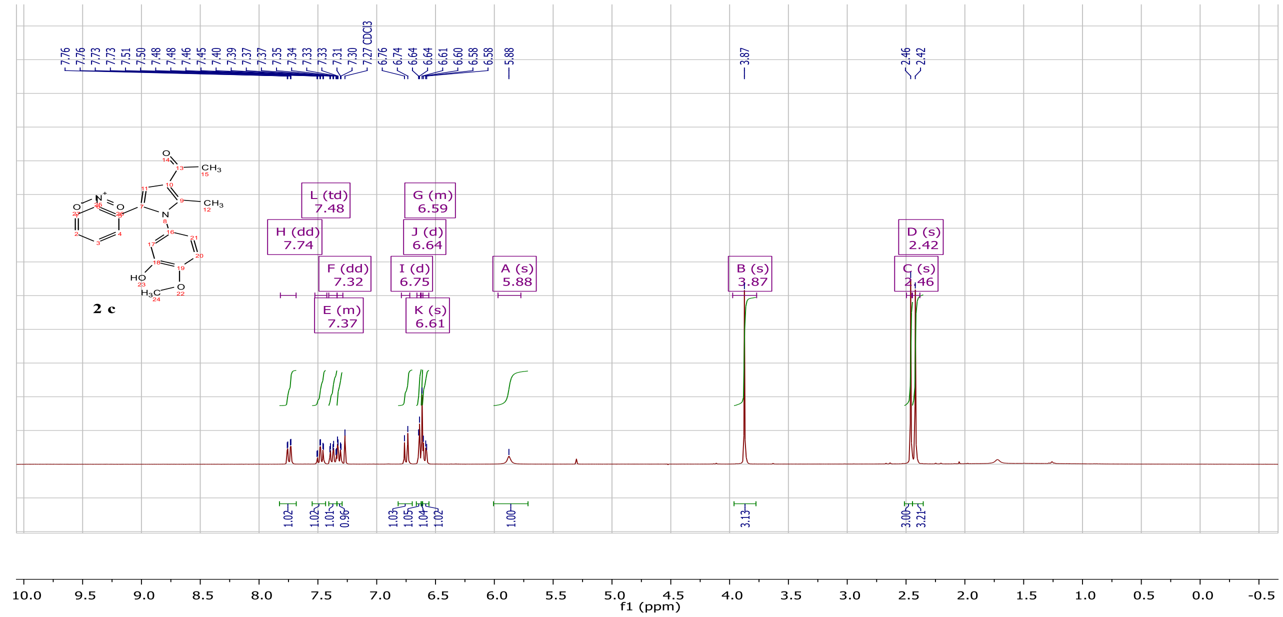

Figure S57. ^{1}HNMR spectra for compound **2c**.

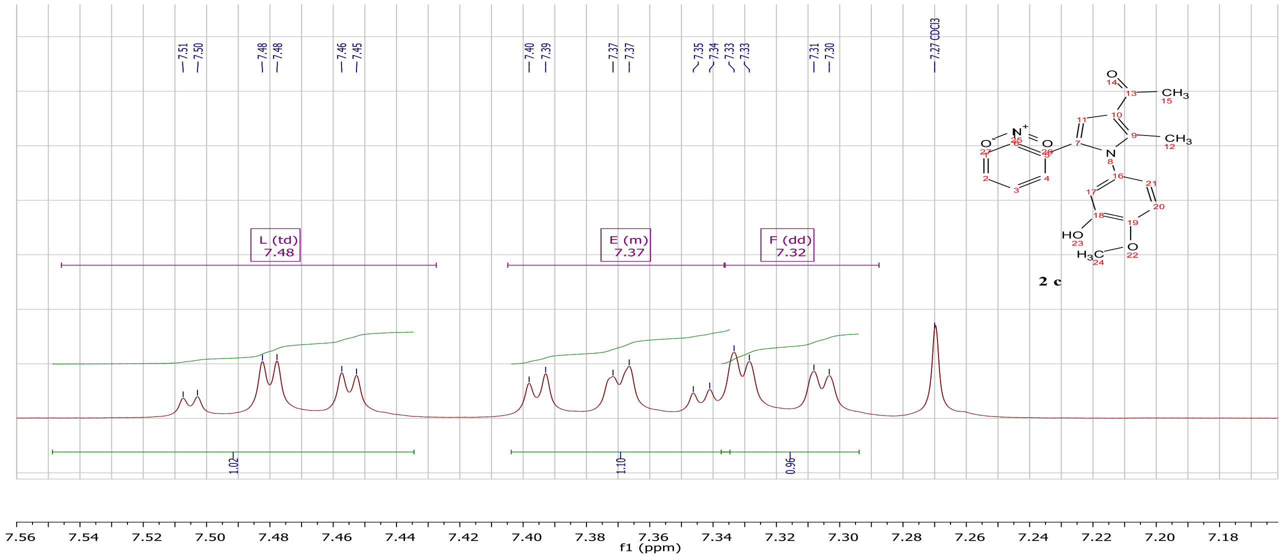

Figure S58. Focused ^{1}HNMR spectra for compound **2c**.

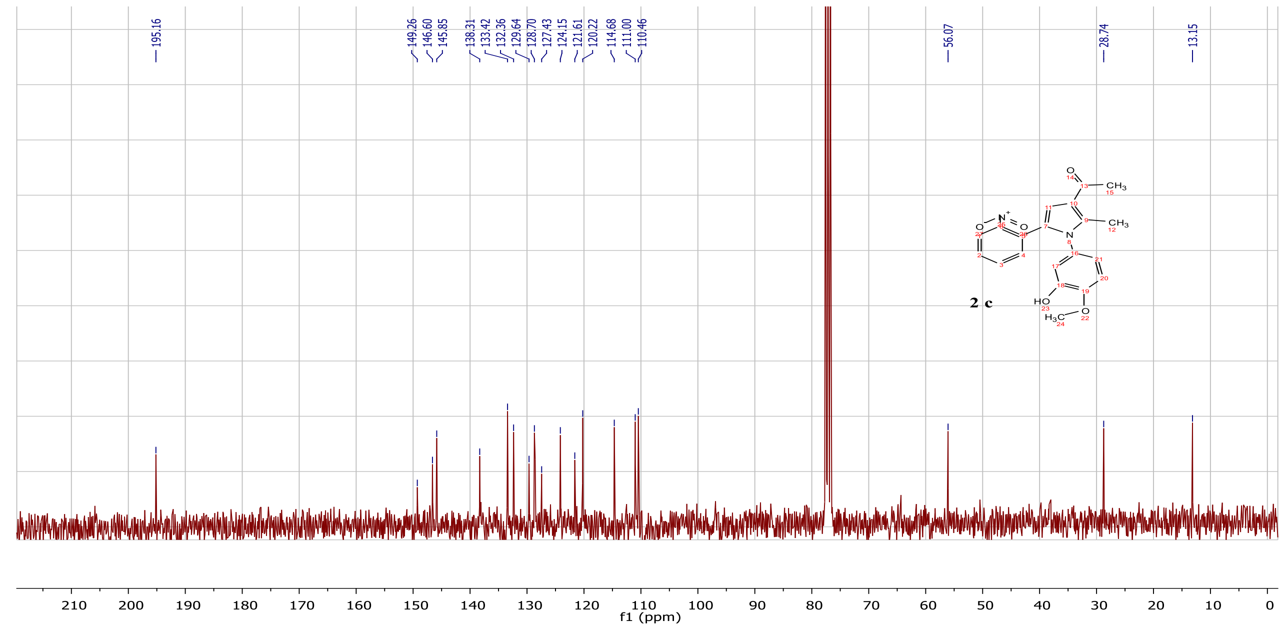

Figure S59. ^{13}CNMR spectra for compound **2c**.

2b

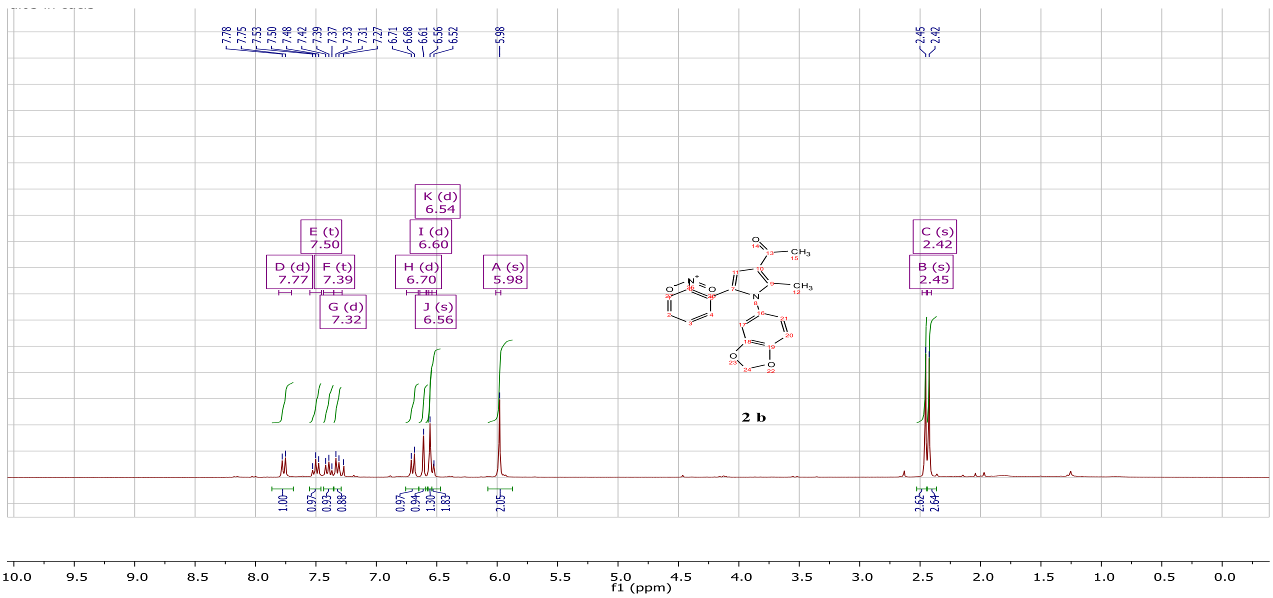

Figure S60. ^{1}HNMR spectra for compound **2b**.

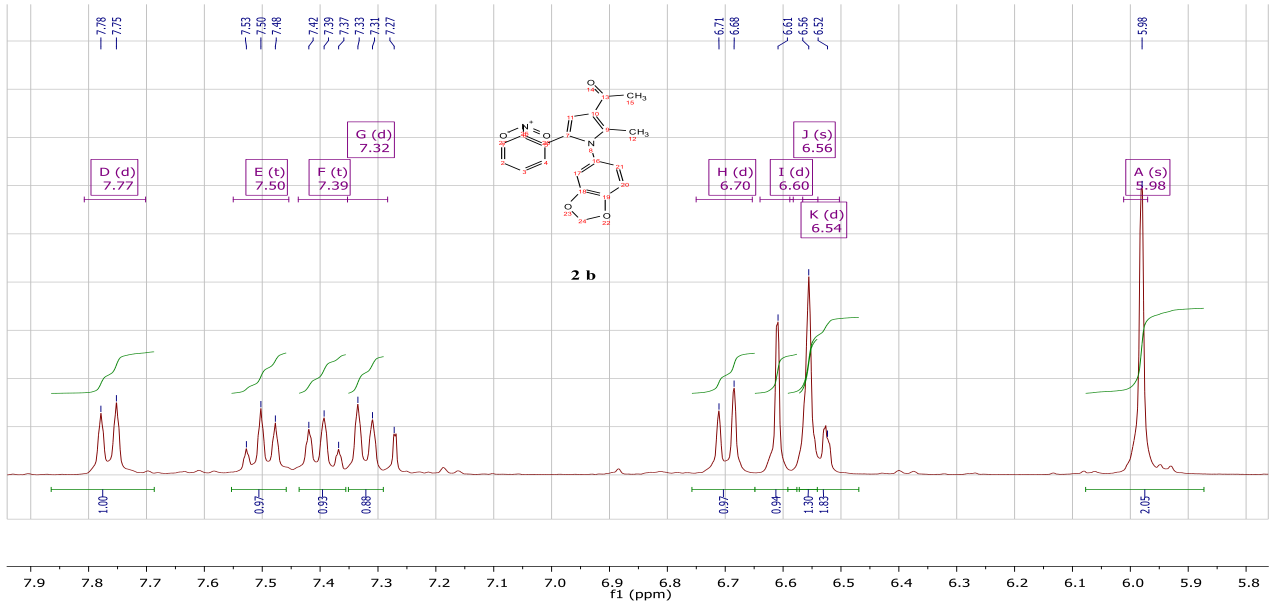

Figure S61. Focused ^{1}HNMR spectra for compound **2b**.

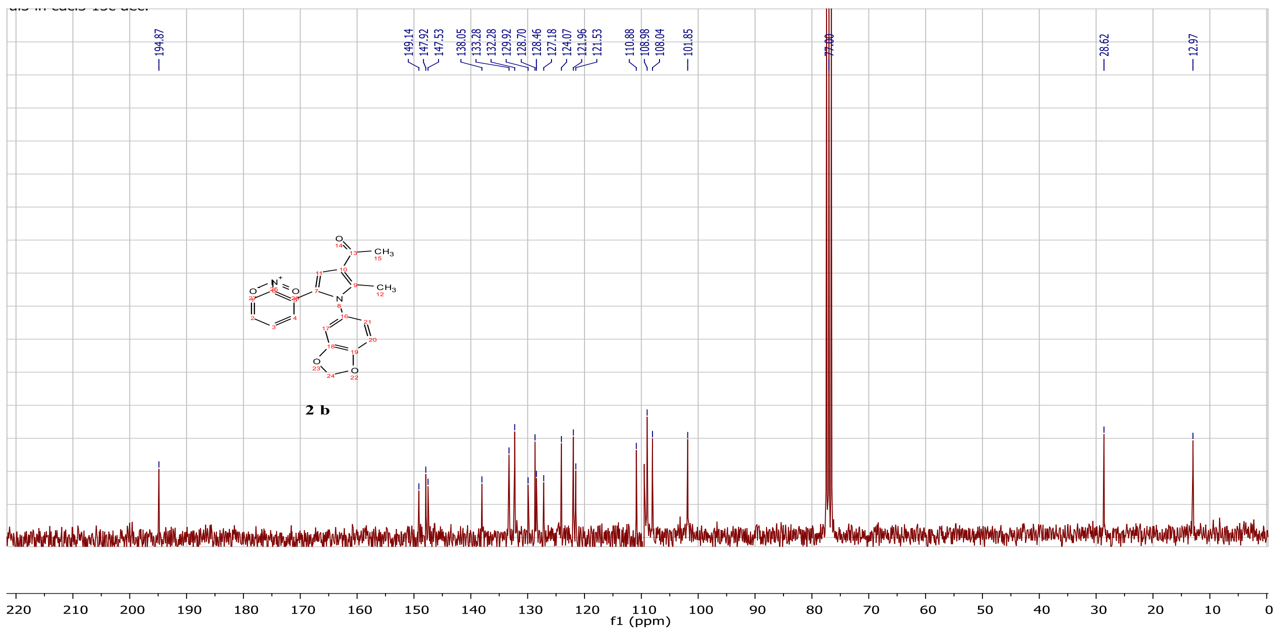

Figure S62. ^{13}CNMR spectra for compound **2b**.

8r

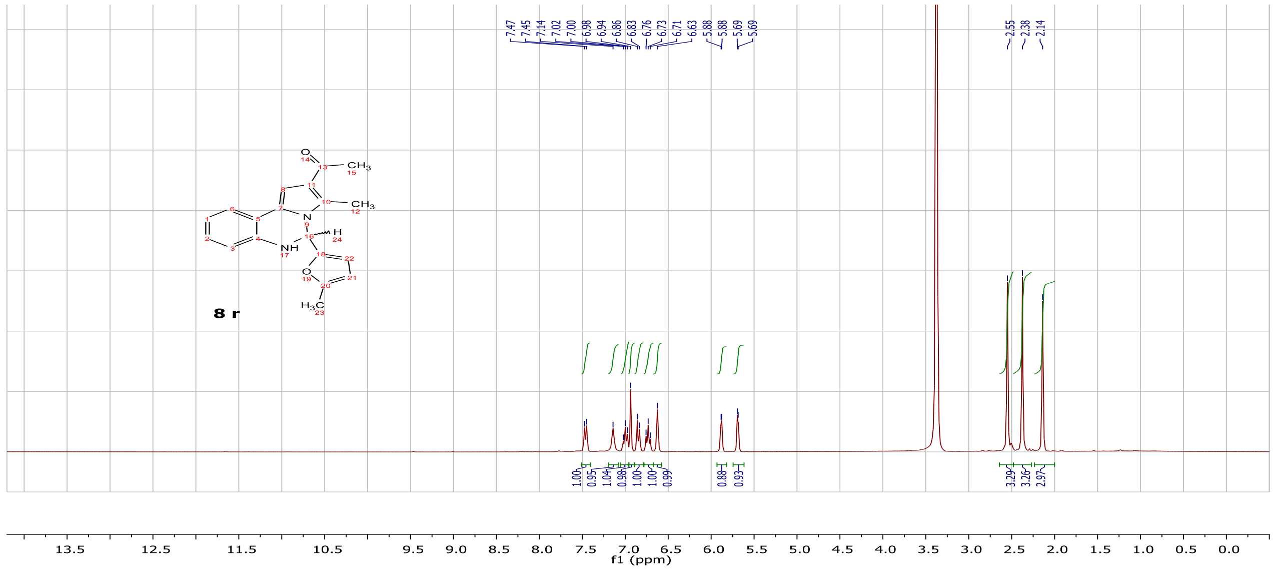

Figure S63. ^{1}HNMR spectra for compound **8r**.

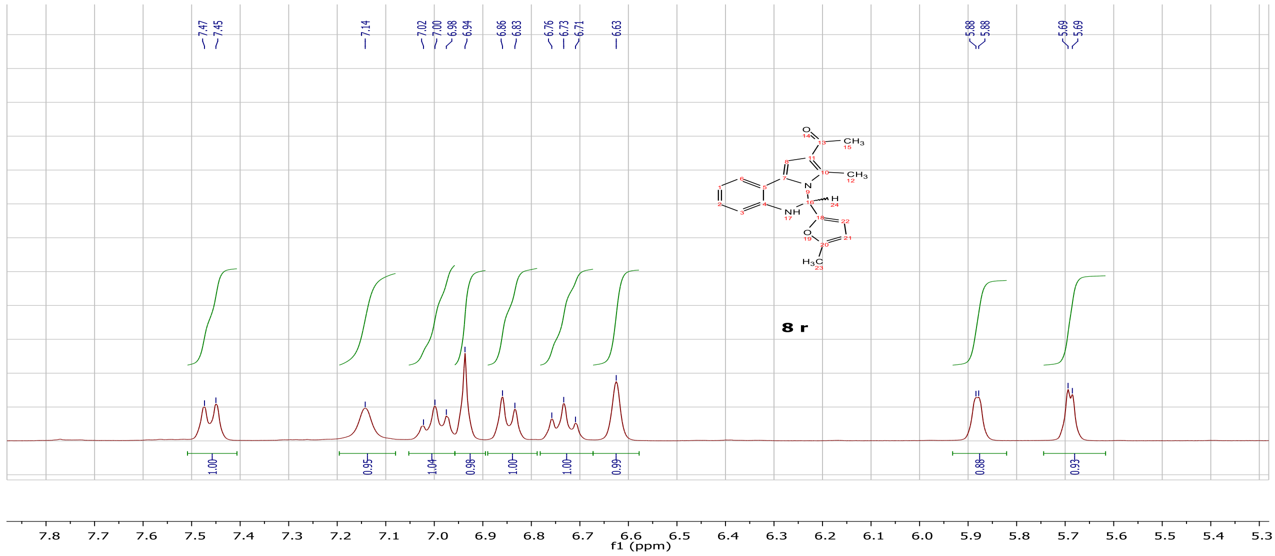

Figure S64. Focused ^{1}HNMR spectra for compound **8e**.

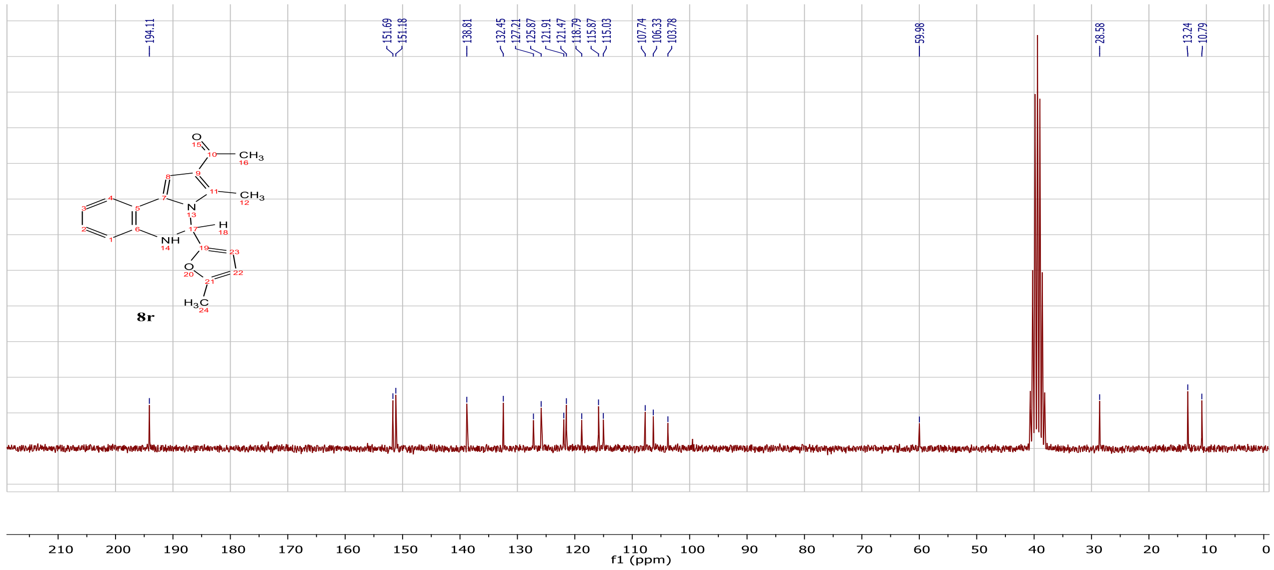

Figure S65. ^{13}CNMR spectra for compound **8r**.

All spectra were processed with MestReNova Software.

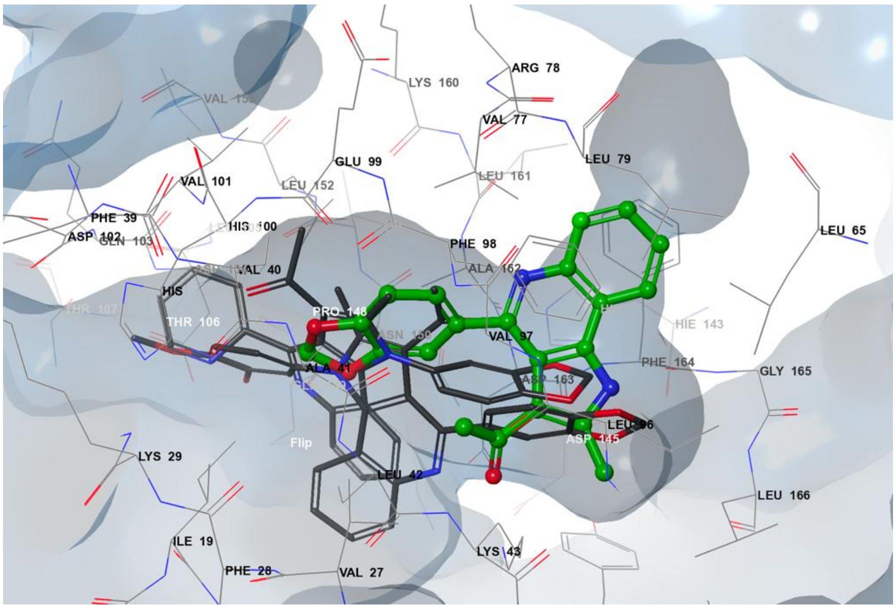

Figure S66. 3D binding poses of compounds **7d**, **7h**, and **7i** within the **CDK6** active site. The figure highlights the different spatial conformations adopted by the ligands, particularly in the orientation of the benzodioxole moiety. Compound **7d** (in green) exhibits a distinct binding orientation compared to **7h** and **7i** (in grey), which share a similar conformation. This conformational divergence may explain the differences in biological activity observed experimentally.

Table S1. Complete SwissADME output for compounds **7d** and **7f**, including calculated physicochemical properties, lipophilicity descriptors, solubility predictions, drug-likeness filters, medicinal chemistry alerts, and ADME-related predictions (GI absorption, BBB permeability, P-gp substrate status, and CYP inhibition profiles).

**Molecule**	**7d**	**7e**	**7f**	**7g**	**7h**	**7i**	**7j**	**7k**	**7l**	**7m**	**7n**	**7o**	**7p**	**7q**
**NCI GI% (SR)**	68.36	27.45	53.14	40.83	34.40	-	-	29.77	52.02	-	-	-	-	-
**NCI GI% (K562)**	-	-	31.63	28.68	29.20	-	-	-	24.96	-	-	-	-	-
**NCI GI% (CCRF-CEM)**	-	-	-	32.41	24.43	-	-	-	-	-	-	-	-	-
**NCI GI% (MOLT4)**	-	-	-	42.18	34.66	-	-	-	-	-	-	-	-	-
**NCI GI% (SNB75)**	53.53	24.38	44.94	34.76	-	-	-	-	-	-	-	-	-	-
**NCI GI% (MDA-MB-435)**	57.89	28.94	-	-	-	-	-	-	-	-	-	-	-	-
**NCI GI% (UO-31)**	73.57	47.06	59.28	44.62	30.57	27.66	31.02	39.37	47.79	-	-	-	-	-
**NCI GI% (A498)**	-	-	-	35.03	-	-	-	-	-	-	-	-	-	-
**NCI GI% (MCF-7)**	58.51	-	26.52	-	-	-	-	-	-	-	-	-	-	-

**NCI GI% (T47D)**	-	-	-	30.30	-	-	-	27.35	-	-	-	-	-	-
**NCI GI% (HCT15)**	-	-	-	-	29.20	-	-	-	-	-	-	-	-	-
**NCI GI% (HOP62)**	-	-	30.28	30.18	-	-	-	-	-	-	-	-	-	-
**NCI GI% (HOP92)**	-	-	30.55	-	-	-	-	-	-	-	-	-	-	-
**NCI GI% (UACC62)**	-	-	45.64	-	-	-	-	-	28.06	-	-	-	-	-
**NCI GI% (NCI-H522)**	-	-	-	-	-	-	-	-	26.25	-	-	-	-	-
**GI_{50} µM (LAN5)**	-	-	-	17.8 ± 2.0	16.6 ± 2.1	77.4 ± 9.5	-	-	-	25.2 ± 3.0	90.8 ± 11.0	27.2 ± 3.2	33.6 ± 4.0	-
**GI_{50} µM (H292)**	-	-	-	91.3 ± 11.0	38.2 ± 4.5	67.2 ± 8.0	-	-	-	55 ± 6.5	92.4 ± 11.0	40.4 ± 4.8	22.4 ± 2.5	-
**GI_{50} µM (16HBE)**	-	-	-	22.2 ± 2.5	17.9 ± 2.0	52 ± 6.0	-	-	-	16.5 ± 2.0	59.4 ± 7.0	22.3 ± 2.6	34.2 ± 4.0	-
**GI_{50} µM (HeLa)**	-	-	-	75.3 ± 9 .0	38.5 ± 4.5	40.3 ± 5.0	-	-	-	38.2 ± 4.5	46.7 ± 5.5	71.3 ± 8.5	16.4 ± 2.0	-
**GI_{50} µM (MCF7)**	-	-	-	10.6 ± 1.2	15.7 ± 1.8	16.7 ± 2.0	-	-	-	18.4 ± 2.2	17.5 ± 2.0	13.6 ± 1.5	2,8 ± 0.3	-
**GI_{50} µM (Caco2)**	-	-	-	20.2 ± 2.4	16.6 ± 2.0	58.5 ± 7.0	-	-	-	28.2 ± 3.3	50.7 ± 6.0	15.8 ± 2.0	25.3 ± 3.0	-
**Heavy atoms**	26	26	26	35	35	32	23	23	23	38	32	38	35	23
**Aromatic heavy atoms**	19	19	19	25	25	25	19	19	19	25	25	25	25	18
**Rotatable bonds**	2	2	3	3	4	3	2	2	2	6	4	7	5	2
**H-bond acceptors**	4	4	4	6	6	5	3	3	3	7	5	7	6	3
**H-bond donors**	1	1	2	0	1	0	1	1	1	0	1	1	2	1
**MR**	100.3	100.3	102.7	131.3	133.8	123	92	92	92	144.7	125.5	147.2	136.2	91.4
**TPSA**	64.21	64.21	75.21	71.81	82.81	66.24	58.64	58.64	58.64	81.04	77.24	92.04	93.81	58.89
**iLOGP**	2.86	2.62	2.51	4.1	4.06	3.58	1.9	2.05	2.05	4.35	3.53	4.33	3.81	2.71
**XLOGP3**	3.88	3.88	3.68	5.3	5.1	4.45	3.03	2.99	2.99	5.4	4.25	5.2	4.91	3.56
**WLOGP**	4.62	4.62	4.61	5.81	5.8	5.48	4.29	4.29	4.29	6.11	5.47	6.1	5.79	4.8
**MLOGP**	2.19	2.19	1.79	3.04	2.63	2.5	1.63	1.63	1.63	2.5	2.09	2.09	2.23	1.67
**Consensus Log P**	3.77	3.72	3.52	4.77	4.59	4.24	3.15	3.17	3.17	4.87	4.06	4.7	4.37	3.61
**ESOL Log S**	-4.83	-4.83	-4.65	-6.39	-6.21	-5.64	-4.1	-4.07	-4.07	-6.5	-5.46	-6.32	-6.04	-4.42
**ESOL Solubility (mg/ml)**	5.11E-03	5.11E-03	7.78E-03	1.90E-04	2.88E-04	9.73E-04	2.41E-02	2.56E-02	2.56E-02	1.62E-04	1.48E-03	2.46E-04	4.31E-04	1.17E-02

**ESOL Solubility (mol/l)**	1.49E-05	1.49E-05	2.25E-05	4.08E-07	6.17E-07	2.31E-06	8.01E-05	8.49E-05	8.49E-05	3.18E-07	3.49E-06	4.80E-07	9.19E-07	3.83E-05
**ESOL Class**	Moderately soluble	Moderately soluble	Moderately soluble	Poorly soluble	Poorly soluble	Moderately soluble	Moderately soluble	Moderately soluble	Moderately soluble	Poorly soluble	Moderately soluble	Poorly soluble	Poorly soluble	Moderately soluble
**Ali Log S**	-4.93	-4.93	-4.95	-6.56	-6.58	-5.56	-3.93	-3.89	-3.89	-6.86	-5.58	-6.88	-6.62	-4.48
**Ali Solubility (mg/ml)**	4.08E-03	4.08E-03	3.89E-03	1.28E-04	1.22E-04	1.16E-03	3.57E-02	3.92E-02	3.92E-02	7.10E-05	1.10E-03	6.76E-05	1.13E-04	1.00E-02
**Ali Solubility (mol/l)**	1.19E-05	1.19E-05	1.12E-05	2.76E-07	2.62E-07	2.75E-06	1.18E-04	1.30E-04	1.30E-04	1.39E-07	2.61E-06	1.32E-07	2.42E-07	3.30E-05
**Ali Class**	Moderately soluble	Moderately soluble	Moderately soluble	Poorly soluble	Poorly soluble	Moderately soluble	Soluble	Soluble	Soluble	Poorly soluble	Moderately soluble	Poorly soluble	Poorly soluble	Moderately soluble
**GI absorption**	High	High	High	High	High	High	High	High	High	High	High	Low	High	High
**BBB permeant**	Yes	Yes	No	No	No	No	Yes	Yes	Yes	No	No	No	No	Yes
**Pgp substrate**	Yes	Yes	Yes	Yes	Yes	Yes	Yes	Yes	Yes	Yes	Yes	Yes	Yes	Yes
**CYP1A2 inhibitor**	Yes	Yes	Yes	No	No	Yes	Yes	Yes	Yes	No	Yes	No	No	Yes
**CYP2C19 inhibitor**	Yes	Yes	Yes	Yes	Yes	Yes	Yes	Yes	Yes	Yes	Yes	Yes	Yes	Yes
**CYP2C9 inhibitor**	Yes	Yes	Yes	Yes	Yes	Yes	No	No	No	Yes	Yes	Yes	Yes	Yes
**CYP2D6 inhibitor**	No	No	Yes	No	No	No	Yes	Yes	Yes	No	Yes	No	Yes	Yes
**CYP3A4 inhibitor**	Yes	Yes	Yes	No	No	Yes	Yes	Yes	Yes	No	Yes	No	No	Yes
**log Kp (cm/s)**	-5.65	-5.65	-5.8	-5.37	-5.52	-5.71	-5.99	-6.02	-6.02	-5.58	-5.87	-5.73	-5.67	-5.63
**Lipinski #violations**	0	0	0	0	0	0	0	0	0	1	0	1	0	0
**Ghose #violations**	0	0	0	2	2	0	0	0	0	3	0	3	2	0
**Veber #violations**	0	0	0	0	0	0	0	0	0	0	0	0	0	0
**Egan #violations**	0	0	0	0	0	0	0	0	0	1	0	1	0	0
**Muegge #violations**	0	0	0	1	1	0	0	0	0	1	0	1	0	0
**Bioavailability Score**	0.55	0.55	0.55	0.55	0.55	0.55	0.55	0.55	0.55	0.55	0.55	0.55	0.55	0.55
**PAINS #alerts**	0	0	0	0	0	0	0	0	0	0	0	0	0	0
**Brenk #alerts**	0	0	0	0	0	0	0	0	0	0	0	0	0	0

**Leadlikeness #violations**	1	1	1	2	2	2	0	0	0	2	2	2	2	1
